# Taxonomic refinement of *Bacillus thuringiensis*

**DOI:** 10.3389/fmicb.2025.1518307

**Published:** 2025-02-07

**Authors:** Nagham Shiekh Suliman, Reza Talaei-Hassanloui, Hamid Abachi, Sadegh Zarei, Ebrahim Osdaghi

**Affiliations:** ^1^Department of Plant Protection, College of Agriculture, University of Tehran, Karaj, Iran; ^2^Department of Plant Protection, College of Agriculture, Isfahan University of Technology, Isfahan, Iran

**Keywords:** *Bacillus*, biological control, phylotaxonomy, bacterial taxonomy, *Bacillus cereus*

## Abstract

*Bacillus thuringiensis* is the most important biological control agent against various agricultural pests. The bacterium taxonomically belongs to the *Bacillus cereus* group, which also contains human pathogenic species, e.g., *Bacillus anthracis*. Thus, precise identification and taxonomic delineation of candidate strains for agricultural usage is of high importance in terms of both public health and biosecurity. By October 2023, whole genome sequences (WGS) of 885 bacterial strains were labeled as *B. thuringiensis* in the NCBI GenBank database. This study investigates the taxonomic authenticity of those strains using DNA similarity indexes, i.e., average nucleotide identity (ANI) and digital DNA–DNA hybridization (dDDH). All strains were compared with the type strain of *B. thuringiensis* ATCC 10972^T^. WGS-based phylotaxonomic investigations showed that out of 885 strains 803 strains authentically belonged to *B. thuringiensis* while 82 strains were mislabeled as *B. thuringiensis* having dDDH and ANI values less than the acceptable threshold of 70 and 95% respectively, for prokaryotic species definition in comparison with the *B. thuringiensis* type strain. Among these 82 mislabeled strains, 39 strains need to be reclassified within the *B. cereus* group in the species *B. anthracis* (33 strains), *Bacillus toyonensis* (five strains), and *Bacillus mycoides* (one strain). Furthermore, four strains were identified as *Bacillus tropicus* while one strain belonged to each of the species *Bacillus licheniformis*, *Bacillus paranthracis*, and *Bacillus weidmannii.* The remaining 36 strains did not match with any known *Bacillus* species nor the species of other bacterial genera, thus they could be assigned to hypothetical new species. Results of the present study, on the one hand, pave the way of comprehensive taxonomic refinements within *B. thuringiensis* species. On the other hand, highlight the role of taxonomic investigations in targeting authentic *B. thuringiensis* strains for biological control purposes.

## Introduction

*Bacillus thuringiensis* is a Gram-positive, rod-shaped, spore-forming bacterium, found usually in soil, grain dust, water, and dead insect bodies (Lambert and Peferoen, 1992). This entomopathogenic species (causeing disease on insects) has been used as a successful biological insecticide for more than 80 years being a specific, safe, and effective tool for controlling a wide variety of insect pests (Nazarian et al., 2009). *Bacillus thuringiensis* belongs to *Bacillus cereus* group, which also contains seven other species, i.e., *Bacillus cereus sensu stricto*, *Bacillus anthracis*, *Bacillus weihenstephanensis*, *Bacillus cytotoxicus*, *Bacillus mycoides*, *Bacillus pseudomycoides*, and *Bacillus toyonensis* (Guinebretière et al., 2013; Jiménez et al., 2013). To distinguish various species of this group from one another, phylogenetic analysis based on the sequences of housekeeping genes and investigation of virulence plasmids can play an important role. However, as virulence plasmids may be transferred or lost in evolution, they are not suitable for the classification (Liu et al., 2015). Furthermore, sole reliance on insecticidal crystal protein genes (*cry*) for identification of *B. thuringiensis* has some limitations. Comparative genomics data indicates that *B. thuringiensis* is not the only species that possesses these genes (Meric et al., 2018; Baek et al., 2019; Castillo-Esparza et al., 2019). Until recently, *B. thuringiensis* strains that were commercialized as pesticides did not have an available complete genome sequence in the public database (Rang et al., 2015; Zhu et al., 2015). *Bacillus thuringiensis* is classified into more than 85 subspecies, which are distinguished by the antigenic characteristics of the flagellar H-antigen (Lecadet et al., 1999; Jakhar et al., 2017).

Classification, nomenclature, and phylogenetic characterization are the three interrelated parts of prokaryotic taxonomy that have historically been recognized (Garrity, 2005). Classification of the genus *Bacillus* has changed significantly over time because of advancements in molecular biology techniques (Maughan and Van der Auwera, 2011). Based on phenotypic similarity metrics, 16S rRNA sequences, rep-PCR (repetitive extragenic palindromic), and DNA–DNA hybridization (DDH) in addition to the presence or absence of virulent plasmids, the bacteria in this genus have historically been divided into several species (Rasko et al., 2005). DNA–DNA hybridization (DDH) has been used since 1960s to elucidate taxonomic relationships between bacteria. This method was widely used as it provides comprehensive comparisons between organisms. Wayne et al. (1987) suggested a 70% DDH value as the gold standard for defining prokaryotic species. For species circumscription, the new gold standards are digital whole genome comparisons using average nucleotide identity (ANI) or genome-to-genome-distance calculations (GGDCs) (Konstantinidis and Tiedje, 2005; Richter and Rosselló-Móra, 2009; Meier-Kolthoff et al., 2013). Next-generation sequencing technologies have led to a change in the approaches used in microbial taxonomy. According to Patil and McHardy (2013), digital DNA–DNA hybridization (dDDH) has replaced classical DDH. For instance, using the dDDH approach Liu et al. (2015) found that some strains previously classified as *B. cereus* or *B. thuringiensis* were *B. anthracis*. Furthermore, considering the evolutionary relationships of bacteria, multilocus sequence analysis (MLSA) is a valuable tool for classifying related strains (Gevers et al., 2005). Because it is based on the allelic differences between several conserved housekeeping genes, MLSA has become a strong tool for classifying bacterial strains (Clarke et al., 2002).

Altogether, correct and precise identification of *B. thuringiensis* within a well-defined taxonomic framework is a prerequisite for initiating biological application of the bacterium. Further, differentiation of *B. thuringiensis* strains from taxonomically closely related species, i.e., *B. cereus* and *B. anthracis* is crucial for biotechnology advancement, food health, and bio-pesticides industry. Hence, this study aims to investigate if all genome sequences deposited as *B. thuringiensis* in the NCBI GenBank database authentically belong to this species. To meet this goal, we have retrieved all publicly available whole genome resources labeled as *B. thuringiensis* in the NCBI GenBank database. The genome sequences were subjected to phylotaxonomic analyses using standard criteria in the taxonomy of prokaryotic species. Results showed that among 885 genome sequences named as *B. thuringiensis*, 82 strains (9.26%) did not belong to this species and were identified as members of either previously defied *Bacillus* species or hypothetical new species within the genus.

## Materials and methods

### Data collection

In October 2023, all publicly available genome sequences labeled as *B. thuringiensis* in the NCBI GenBank database were retrieved and subjected to phylotaxonomic analyses. Furthermore, the *gyrB* gene sequence in *B. thuringiensis* ATCC 10972^T^ was used to find putative mislabeled *B. thuringiensis* strains in the NCBI GenBank database with the BLAST engine. In total, 885 whole-genome sequences that were classified as *B. thuringiensis* were obtained from the NCBI GenBank database and subjected to the following analyses (Supplementary Table S1). The genome quality evaluation was conducted using the BUSCO software, a widely recognized tool for assessing genome assembly completeness by examining the presence of conserved single-copy orthologs (Seppey et al., 2019). The version employed in this study was v5.2.2, which offers robust and standardized metrics for genome quality assessment Manni et al. (2021).

### Phylogenetic analyses

All genome sequences were uploaded to the Galaxy Europe platform ^1^for phylogenetic analysis and comparative genomics. Prokka v1.14.6 was used to reannotate all genome sequences on the Galaxy Europe platform. The resulting gff3 files were then utilized to build a pan-genome using Roary v3.13.0 (Goecks et al., 2010; Page et al., 2015; Seemann, 2014; Stein, 2013). MEGA software v7.0 was used to visualize the resulting phylogenetic trees (Kumar et al., 2016). For each clade of the core genome-based phylogenetic tree, dDDH and ANI were calculated in comparison with the type strain of *B. thuringiensis* ATCC 10972^T^. Members of the clades that had a strain with ANI and dDDH less than 95 and 70% respectively, with *B. thuringiensis* ATCC 10972^T^ were selected for further analyses.

In order to elucidate if the results of core genome-based phylotaxonomic analyses were in congruence with the routine MLSA, all 885 whole-genome sequences were subjected to the five-gene-based phylogenetic analyses as recommended for *Bacillus* spp. (Guinebretière et al., 2013). Hence, sequences of the five housekeeping genes *atpD*, *dnaK*, *gyrB*, *rpoB*, and *rpoD* were extracted from the whole genome sequences of all strains using the BLAST engine of NCBI. The sequences were aligned using MAFFT online service v7.490 ^2^ (Katoh et al., 2019) and subjected to phylogenetic analyses either alone or in concatenation of five housekeeping genes. The phylogenetic trees were constructed and visualized via MEGA software v7.0 using the recommended procedure.

### Taxonomic refinements

Preliminary phylogenetic analyses showed that out of 885 whole-genome sequences retrieved from the NCBI GenBank with *B. thuringiensis* label, 82 strains clustered separately from the core members of the species. To determine the taxonomic relationships of these 82 strains that were possibly incorrectly classified as *B. thuringiensis*, and define a taxonomic status for a particular taxon, the ANI and dDDH indices were calculated for all these strains. The latter analyses showed that the 82 strains had dDDH and ANI less than the accepted threshold for the definition of prokaryotic species with the type strain of *B. thuringiensis* ATCC 10972^T^ (Kim et al., 2014). Thus, for these 82 strains, ANI was calculated by comparing them with each other and with type strains of *Bacillus* spp. using ANIm (Pritchard et al., 2016). dDDH was also calculated using the Genome-to-Genome Distance Calculator v.3.1 (Meier-Kolthoff et al., 2022).

According to the list of prokaryotic names with standing in nomenclature (LPSN)^3^, by November 2023, 110 *Bacillus* spp. species were validly published in the literature. Out of these 110 species, whole genome sequences of 97 species were publicly available as shown in Supplementary Table S2. Thus, all the latter 97 strains were included in the analyses. For whole-genome sequence-based classification of the bacterial strains, the genome assemblies were used as input for the pyANI v0.2.11 Python pipeline (Pritchard et al., 2016), with the ANIm parameter to calculate pairwise distances using MUMmer (nucmer). The pipeline generates a distance matrix and a double hierarchical clustered heatmap, in which a red color indicates ANI percent identities above 95%. In the result of pyANI, if one strain in comparison with a type strain had ANI and dDDH values more than 95 and 70% respectively, it was considered to belong to the species of the corresponding type strain.

Type Strain Genome Server (TYGS)^4^ by comparing the user genomes with its collection of type strains consisting of currently 21,334 microbial type strain genomes helps to understand taxonomic status of the query strain. The results are presented in the form of a 16S rRNA-based phylogenetic tree and whole genome-based phylogeny (Meier-Kolthoff and Göker, 2019). To determine the taxonomic status of the strains with low similarity to the *B. thuringiensis* type strain, and to verify the validity of the results reached by the WGS-based phylogenetic tree, MLSA-based phylogenetic tree, and pyANI, the genomes of 82 suspected strains were uploaded to the TYGS website and the resulting data was presented in the form of phylogenetic trees.

## Results

By October 2023, the NCBI GenBank database contained 885 whole-genome sequences labeled as *B. thuringiensis* (Supplementary Table S1). Using ANI, dDDH, core genome-based phylogeny, and MLSA taxonomic authenticity of those 885 strains was investigated. Supplementary Table S1 highlights the strains with lower genome quality. These strains are also indicated within the MLSA-based phylogenetic tree presented in Supplementary Figure S1. Notably, four strains, i.e., SRR5713945-bin, XL6, F14-1, and s1783 are absent from this tree. Their exclusion is due to their failure to meet the minimum genomic criteria for constructing the phylogenetic tree, as they lack one or more of the five housekeeping genes *atpD*, *dnaK*, *gyrB*, *rpoB*, and *rpoD* used in MLSA-based phylogenetic analysis. This limitation underscores the importance of genome quality in ensuring the reliability and comprehensiveness of phylogenetic and comparative analyses. The strains missing from the MLSA phylogenetic tree further exemplify how incomplete genomic assemblies can hinder accurate evolutionary interpretations.

Out of 885 strains, 803 strains were confirmed to belong to *B. thuringiensis*, while 82 strains were incorrectly labeled as *B. thuringiensis* considering the above-mentioned criteria. The latter 82 strains had low DNA similarity indices with the *B. thuringiensis* type strain (Table 1). Out of 82 strains incorrectly classified as *B. thuringiensis*, 39 strains should be transferred to other species belonging to the *B. cereus* group including *B. anthracis*, *B. toyonensis*, and *B. mycoides*. Taxonomic status of 46 strains was clarified as follows: 33 strains belonged to *B. anthracis* (Supplementary Table S3). Five strains belonged to *B. toyonensis* (Table 2), and four strains were identified as *B. tropicus* (Table 3). Four strains were related to different species as follows: Et10/1 belonged to *Bacillus licheniformis* with 99.81 and 97.60% ANI and dDDH, respectively, BGSC 4BM1 belonged to *B. mycoides* with 97.37 and 74.80% ANI and dDDH, respectively, 4I3 belonged to *Bacillus paranthracis* with 97.43 and 77.30% ANI and dDDH respectively, and BGSC 4BV1 belonged to *Bacillus weidmannii* with 96.95 and 72.20% ANI and dDDH, respectively.

**Table 1:** The list of 82 strains mislabeled as *Bacillus thuringiensis* with ANI and DDH less than 95 and 70%, respectively, compared to type strain of the species.

Strain	dDDH	ANI	Isolation source	Accession number	Origin	Year	Reference	Identified as
4B3	44.00	91.61	Unknown	WJDN01000000	Unknown	Unknown	Not specified	*Bacillus anthracis*
4I3	44.30	91.72	Unknown	WJCV01000000	Unknown	Unknown	Not specified	*Bacillus paranthracis*
4 W2	44.30	91.66	Unknown	WJCD00000000	Unknown	Unknown	Not specified	*Bacillus anthracis*
4XX2	43.70	91.53	Unknown	WJCB00000000	Unknown	Unknown	Not specified	*Bacillus anthracis*
4XX1	43.60	91.53	Unknown	NZ_NFEI00000000	Unknown	Unknown	Not specified	*Bacillus anthracis*
4XX3	43.90	91.57	Unknown	NZ_NFEI00000000	Unknown	Unknown	Not specified	*Bacillus anthracis*
97–27	44.30	91.73	Unknown	AE017355	Unknown	Unknown	Han et al. (2006)	*Bacillus anthracis*
Al Hakam	44.20	91.69	Unknown	CP000485	Unknown	Unknown	Challacombe et al. (2007)	*Bacillus anthracis*
BGSC 4 AC1	43.80	91.60	Soil	NFCF01000000	Mexico	1988	Not specified	*Bacillus anthracis*
BGSC 4AJ1	43.80	91.57	Unknown	CM000752	Unknown	Unknown	Not specified	*Bacillus anthracis*
BGSC 4AL1	42.60	91.28	Unknown	NFCL01000000	South Korea	1994	Not specified	Unidentified
BGSC 4AS1	43.80	91.63	Black pepper power	MOOJ01000000	Brazil	Unknown	Not specified	*Bacillus anthracis*
BGSC 4AW1	44.20	91.65	Unknown	CM000754	Unknown	Unknown	Not specified	*Bacillus anthracis*
XL6	45.10	91.90	Soil	CP013000	China	2006	Not specified	*Bacillus tropicus*
SS2	45.60	92.02	Soil	JAOWLZ010000000	Nigeria	2017	Not specified	Unidentified
SaN0-19	44.10	91.63	Sediment	JAIVKP010000000	China	2021	Not specified	Unidentified
s1783	25.30	84.34	Clay soil	JACYOE000000000	Brazil	2015	Not specified	Unidentified
NRRL B23152	44.90	91.83	Isoptera	PGDW00000000	United Kingdom	2013	Not specified	*Bacillus tropicus*
MC28	44.50	91.79	Unknown	CP003687	Unknown	Unknown	Not specified	*Bacillus toyonensis*
Lr3/2	43.80	91.60	Water	JYCH01000001	Chile	2011	Not specified	Unidentified
LP-2-YM	43.50	91.59	Unknown	SMDG01000000	USA	Unknown	Not specified	Unidentified
LP-1-YM	43.50	91.58	Unknown	SMDF01000000	USA	Unknown	Not specified	Unidentified
LM1212	45.10	91.98	Cadaver of an Oryctes gigas larva	AYPV01000000	Madagascar	Unknown	Liu et al. (2014)	Unidentified
KF1	45.20	92.02	Soil	CP085409	China	2016	Not specified	*Bacillus basilensis*
IEBC_T61001	43.50	91.56	Unknown	FMBI01000000	Unknown	Unknown	Not specified	Unidentified
HSY204	44.20	91.67	Soil	JAHXRX010000000	China	2016	Wu et al. (2021)	Unidentified
HD1011	44.30	91.72	Unknown	CP009335	India	1915	Johnson et al. (2015)	*Bacillus anthracis*
HD682	44.20	91.69	Unknown	CP009720	Unknown	Unknown	Johnson et al. (2015)	*Bacillus anthracis*
HD571	44.20	91.69	Unknown	CP009600	Unknown	Unknown	Johnson et al. (2015)	*Bacillus anthracis*
H3	44.80	91.87	Soil	CP052061	Lebanon	2010	Not specified	*Bacillus toyonensis*
GOE7	44.30	91.71	Tomato rhizosphere	LXLL01000000	Germany	2014	Not specified	*Bacillus toyonensis*
GOE5	44.30	91.74	tomato rhizosphere	LXLJ01000000	Germany	2014	Not specified	*Bacillus toyonensis*
G25-53	45.50	91.74	Maybe soil	LDFU00000000	USA	2011	Not specified	*Bacillus anthracis*
G25-52	43.60	91.59	Maybe soil	LDFT00000000	USA	2011	Not specified	*Bacillus anthracis*
G25-5	45.10	91.90	soil	LDJQ00000000	USA	2011	Not specified	Unidentified
G25-42	43.70	91.65	Soil	LDER00000000	USA	2012	Not specified	*Bacillus anthracis*
FDAARGOS_795	44.30	91.71	Unknown	CP053980	USA	Unknown	Not specified	*Bacillus anthracis*
FDAARGOS_794	44.20	91.68	Unknown	CP053934	USA	Unknown	Not specified	*Bacillus toyonensis*
FDAARGOS_793	44.20	91.69	Unknown	CP053981	USA	Unknown	Not specified	*Bacillus anthracis*
FDAARGOS_792	44.30	91.73	Unknown	CP053938	USA	Unknown	Not specified	*Bacillus anthracis*
FDAARGOS_791	45,20	91.92	Unknown	CP054568	USA	Unknown	Not specified	*Bacillus anthracis*
Et10/1	43,70	91.60	Water	JYCI01000000	Chile	2011	Not specified	*Bacillus lichemiformis*
DPC6431	43.60	91.59	*Homo sapiens*	SCLP01000000	Ireland	2008	Not specified	*Bacillus anthracis*
DE0537	43.80	91.61	Environmental	VDPB01000000	USA	2018	Not specified	*Bacillus anthracis*
DE0555	43.80	91.56	Environmental	VDTR01000000	USA	2018	Not specified	Unidentified
DE0472	42.20	91.13	Environmental	VDRD01000000	USA	2018	Not specified	Unidentified
DE0343	42.20	91.12	Environmental	VEAC01000000	USA	2018	Not specified	Unidentified
DE0163	43.90	91.53	Environmental	VEES01000000	USA	2018	Not specified	*Bacillus anthracis*
DE0141	42.30	91.17	Environmental	VEFL01000000	USA	2018	Not specified	Unidentified
CTC	45.80	92.11	Invertebrates	CP013274	China	1999	Not specified	Unidentified
Bto-UNVM_94	44.00	91.65	soil	QGLX01000000	Argentina	2015	Sauka et al. (2022)	*Bacillus anthracis*
Bt Gxmzu777-1	38.20	89.94	Soil	CP097257	China	2022	Not specified	Unidentified
BM-BT15426	44.80	91.87	Unknown	CP020723	China	2015	Liu et al. (2017a)	*Bacillus anthracis*
KB1	45.10	92.02	*Arabidopsis thaliana*	LSNJ01000000	South Korea	2012	Jeong et al. (2016)	*Bacillus tropicus*
BGSC 4Y1	45.10	91.96	Unknown	CM000746	Unknown	Unknown	Not specified	*Bacillus tropicus*
BGSC 4 CE1	44.00	91.64	Soil	NFDQ01000000	Portugal	Unknown	not Specified	*Bacillus anthracis*
BGSC 4CC1	44.10	91.63	Unknown	CM000757	Unknown	Unknown	Not specified	*Bacillus anthracis*
BGSC 4BY1	43.70	91.60	Scotch pine	MOOQ01000000	Denmark	unknown	Not specified	Unidentified
BGSC 4BX1	43.80	91.60	Sandy soil	NFDL01000000	China	Unknown	Not specified	*Bacillus anthracis*
BGSC 4BL1	44.80	91.85	Unknown	NFDF01000000	Argentina	Unknown	Not specified	Unidentified
BGSC 4 BC1	42.50	91.26	Soil	NFCZ01000000	China	Unknown	Not specified	Unidentified
BGSC 4BJ1	43.70	91.60	soil	NFDD01000000	Poland	unknown	not specified	Unidentified
BGSC 4BV1	43.60	91.60	Unknown	MOOO01000000	Argentina	Unknown	Not specified	*Bacillus wiedmannii*
BGSC 4BM1	38.20	90.00	Soil	NFDG01000000	Spain	Unknown	Not specified	*Bacillus mycoides*
BGSC 4BB1	42.60	91.28	Unknown	NFCY01000000	South Korea	Unknown	Not specified	Unidentified
BGSC 4BA1	44.20	91.66	Unknown	CM000755	Unknown	Unknown	Not specified	*Bacillus anthracis*
BGSC 4AY1	43.60	91.53	Black pepper power	NFCU01000000	Brazil	Unknown	Not specified	*Bacillus anthracis*
45 L	44.00	91.60	Leaf	JAQOOH000000000	Bangladesh	Bangladesh	Not specified	*Bacillus anthracis*
YBT-020	44.30	91.74	Unknown	CP002508	Unknown	Unknown	Zhu et al. (2011)	Unidentified
BGSC 4AH1	44.50	91.80	Unknown	MOOF01000000	South Korea	1994	Not specified	Unidentified
08_128	42.60	91.51	Food	DAOIWC000000000	France	2008	Souvorov et al. (2018)	Unidentified
261–1	45.80	92.02	Soil	NHNP01000000	China	2003	Not specified	Unidentified
AFS065631	45.10	91.93	Plant core	NVCL01000000	USA	2014	Not specified	Unidentified
BGSC 4BG1	42.70	91.30	Ivy leaves	NFDB01000000	Denmark	Unknown	Not specified	Unidentified
BGSC 4BH1	45.00	91.90	Rice paddy	NFDC01000000	Thailand	Unknown	Not specified	Unidentified
BGSC 4CD1	44.80	91.91	Soil	NFDP01000000	Portugal	Unknown	Not specified	Unidentified
DE0326	44.60	91.78	Environmental	VEAN01000000	USA	2018	Not specified	Unidentified
NRRL B-23139	43.70	91.59	Soil	CP035727	Russia	2017	Not specified	Unidentified
patient-SAMN36761919	43.90	91.60	Human blood culture	DAPQOF000000000	USA	2008	Souvorov et al. (2018)	*Bacillus anthracis*
s1930	42.80	91.44	Clay soil	JACYOF000000000	Brazil	2002	not specified	Unidentified
tcg1-2	44.10	91.73	Soil	DAOJID000000000	Japan	2011	Souvorov et al. (2018)	Unidentified
tky2-1	43.40	91.55	Soil	DAOJIE000000000	Japan	2011	Souvorov et al. (2018)	Unidentified

**Table 2:** Digital DNA–DNA hybridization (dDDH; upper diagonal) and average nucleotide identity (ANIm; lower diagonal) values generated from the DNA sequence similarity comparisons among different mislabeled *Bacillus thuringiensis* strains and the *Bacillus toyonensis* type strain.

	Strain	Taxon	1	2	3	4	5	6	7
1	MC28	*B. thuringiensis*		94.60	86.90	87.30	86.70	44.60	88.40
2	H3	*B. thuringiensis*	99.47		86.60	86.80	86.10	44.90	87.60
3	GOE7	*B. thuringiensis*	98.65	98.60		97.90	89.50	44.40	91.60
4	GOE5	*B. thuringiensis*	98.68	98.63	99.78		90.30	44.40	91.90
5	Bto-UNVM_94	*B. thuringiensis*	98.61	98.58	98.90	99.00		44.00	94.60
6	ATCC 10792^T^	*B. thuringiensis*	91.79	91.87	91.71	91.74	91.65		45.20
7	BCT 7112^T^	*B. toyonensis*	98.75	98.71	99.10	99.11	99.42	91.91	

T: type strain.

**Table 3:** Digital DNA–DNA hybridization (dDDH; upper diagonal) and average nucleotide identity (ANIm; lower diagonal) values generated from the DNA sequence similarity comparisons among different mislabeled *Bacillus thuringiensis* strains and type strain of *Bacillus tropicus.*

	Strain	Taxon	1	2	3	4	5	6
1	NRRL B23152	*B. thuringiensis*		71.90	71.10	71.30	44.70	70.70
2	BGSC 4Y1	*B. thuringiensis*	96.91		99.90	94.70	45.10	72.80
3	XL6	*B. thuringiensis*	96.81	99.99		93.90	45.10	71.80
4	KB1	*B. thuringiensis*	96.66	99.31	99.24		45.10	72.10
5	ATCC 10792^T^	*B. thuringiensis*	91.79	91.96	91.90	91.64		45.00
6	N24^T^	*B. tropicus*	96.71	96.95	96.81	96.81	91.87	

T: type strain.

**Figure 1:**
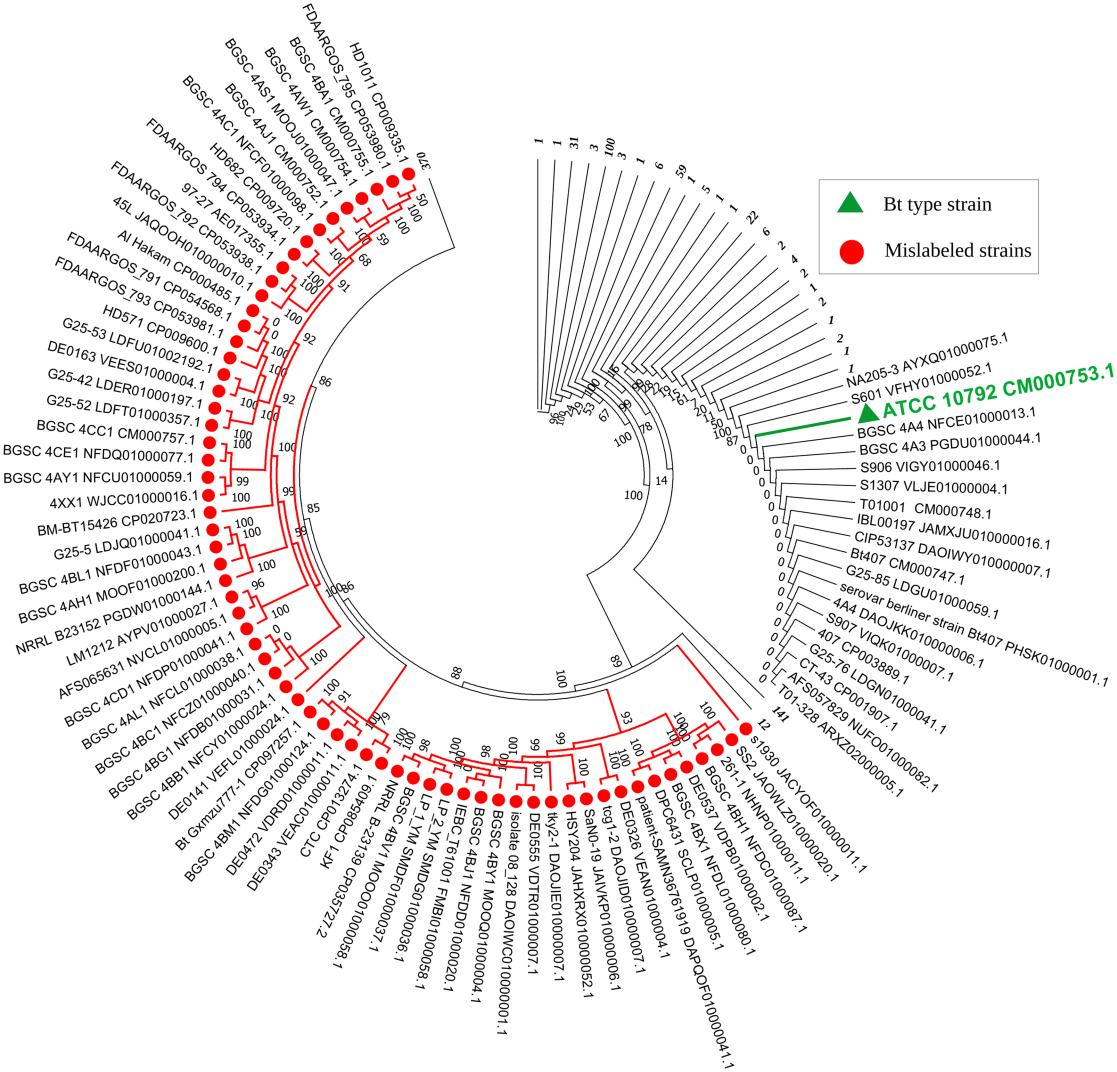
Circular representation of the MLSA-based phylogenetic tree of *Bacillus thuringiensis* strains retrieved from the NCBI database. The number of strains in each compressed branch is shown in italics and bold in front of the corresponding branch. A comprehensive view of the collapsed strains can be found in Supplementary Figure S1. The 82 strains that did not belong to *Bacillus thuringiensis* are shown in red, while the type strain of the species is shown in green.

The remaining 36 strains did not match with any known *Bacillus* species nor the species of other bacterial genera, thus they could be assigned to hypothetical new species (Table 1). In the resulting core genome- and MLSA-based phylogenetic trees, all 82 strains that did not belong to *B. thuringiensis* were clustered together, demonstrating their distinct phylogenetic status (Supplementary Figure S1; Figure 1). On the other hand, BLAST-based explorations showed that three strains that were incorrectly labeled as *B. toyonensis* in the NCBI GenBank should belong to *B. thuringiensis* (Table 4).

**Table 4:** Digital DNA–DNA hybridization (dDDH; upper diagonal) and average nucleotide identity (ANIm; lower diagonal) values generated from the DNA sequence similarity comparisons among different mislabeled *Bacillus toyonensis* strains and type strain of that species.

	Strain	Taxon		1	2	3	4
1	TYU3	*B. toyonensis*		99.80	69.00	45.60	82.60
2	TYU4	*B. toyonensis*	99.88		69.10	45.60	82.60
3	JJ1873	*B. toyonensis*	96.37	96.38		45.60	68.70
5	BCT 7112^T^	*B. toyonensis*	91.74	91.63	91.70		45.20
6	ATCC 10792^T^	*B. thuringiensis*	98.12	98.12	96.29	91.61	

T: type strain.

**Figure 2:**
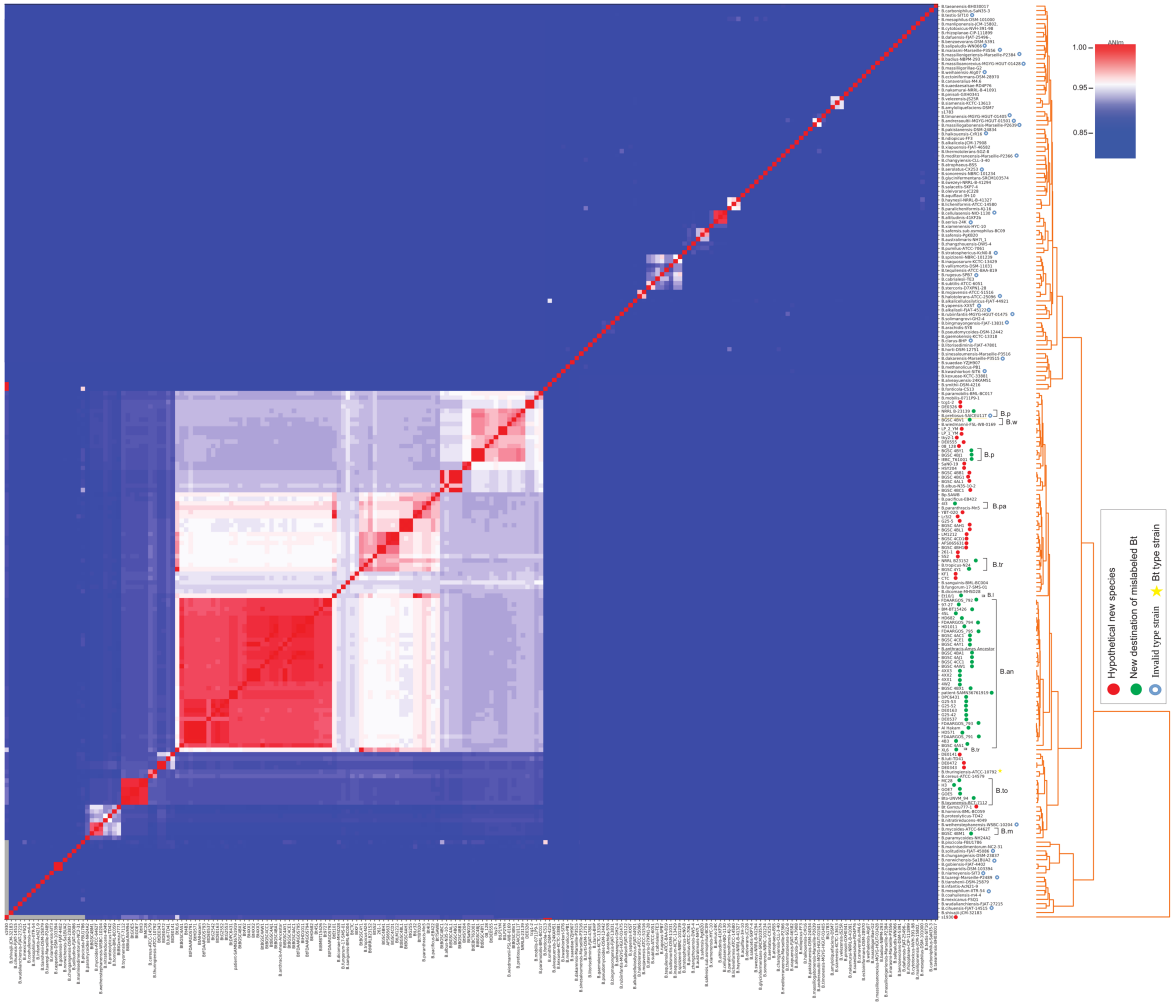
Average nucleotide identity-based pyANI matrix demonstrating nucleotide-level genomic similarity between the 82 mislabeled *Bacillus thuringiensis* strains and type strains of all validly described *Bacillus* species (Pritchard et al., 2016). B.p, *Bacillus pretiosus*; B.w, *Bacillus wiedmannii*; B.pa, *Bacillus paranthracis*; B.tr, *Bacillus tropicus*; B.l, *Bacillus licheniformis*; B.an, *Bacillus anthracis*; B.to, *Bacillus toyonensis*; B.m, *Bacillus mycoides*.

All 82 mislabeled *B. thuringiensis* strains, i.e., 46 strains that were assigned to a valid species and 36 strains with undetermined taxonomic status were subjected to a taxonomic investigation in TYGS. Regarding the former 46 strains, the results of TYGS were in congruence with the pyANI data (Figure 2; Supplementary Figure S2). As for the 36 strains that do not belong to any species of the genus *Bacillus*, the results of TYGS were similar to what was described above (Figure 2; Supplementary Figure S3), except for the strain KF1 where TYGS results showed that this strain belongs to *B. basilensis*. However, WGS of the type strain of *B. basilensis* is not publicly available, thus, we could not confirm the TYGS results using another WGS-based approach. Since the TYGS platform contains a very large number of type strains, including type strain of *B. basilensis*, it was possible to determine the taxonomic status of the strain based on the 16S rDNA data.

Out of 36 mislabeled strains that did not belong to any species, the strains LP_2_YM and LP_1_YM are members of the same species, while the strains SaN0-19 and HSY204 belong to the same species. The strains BGSC 4AL1, BGSC 4BB1, BGSC 4 BC1, and BGSC 4BG1 were members of the same species, the strains DE0472 and DE0343 are classified within the same species, the strains LM1212, BGSC 4CD1, AFS065631, BGSC 4BH1, and 261–1 fall into the same species, while the strains G25-5, BGSC 4BL1, and BGSC 4AH1 belong to the same species. The strains tcg1-2 and DE0326 were members of the same species, and the strains YBT-020 and Lr3/2 belonged to the same species. Strains BGSC 4BY1, IEBC_T61001, BGSC 4BJ1, and NRRL B-23139 were confirmed to belong to *Bacillus pretiosus*, as they showed dDDH values >70% and ANI values >95% with the latter species. These results support the classification of these strains within the species *B. pretiosus*. Additionally, the type strain of *B. pretiosus* SAICEU11^T^ showed no significant genomic similarity with the other type strains in the genus *Bacillus* and this species continues to be listed as a species with an invalid name. Similarly, the strain s1783 is assigned to *Lysinibacillus pinottii*; however, this species is considered to have an invalid name. In conclusion, although genomic similarities clearly indicated the assignment of the mentioned examined strains to the species *B. pretiosus* and *L. pinottii*, these two species were considered invalid at the time of this study. This underscores the necessity for more comprehensive taxonomic evaluations and validation of their nomenclature. It is concluded that the number of possible new species to which these 36 strains belong is 18 novel species. The analysis conducted in TYGS confirmed the accuracy of the WGS-based results as shown in Supplementary Figures S2, S3.

## Discussion

Classification of the *B. cereus* group has always been a controversial issue considering the high possibility of error. This is due in part to the fact that taxonomy of species belonging to this group has been conducted based on phenotypic characteristics (e.g., virulence repertories) while corresponding genes of those phenotypic characteristics are located in most cases on the plasmid, which can be transmitted between close species via conjugation process. In this study, we used phylogenomics and comparative genomics on all available whole genome resources of *B. thuringiensis* to refine taxonomy of the species. Our results showed that out of 885 strains deposited in the NCBI GenBank database, 803 are taxonomically genuine belonging to the core clade of the species regardless of their sources, biological characteristics, and pathogenicity status on the host of isolation. However, 82 strains do not belong to the species *B. thuringiensis* because of the low ANI and dDDH similarities between the type strain of the species and those 82 WGS. Among the mislabeled 82 strains, 39 strains belonged to the *B. cereus* group including *B. anthracis*, *B. toyonensis*, and *B. mycoides*. In addition to these three taxa, mislabeled strains of *B. thuringiensis* were scattered within the genus belonging to several hypothetical novel species, which need formal descriptions.

Although the species belonging to the *B. cereus* group are phylogenetically closely related, they exhibit notable differences in biological characteristics and phenotypic features. This group includes two species *B. anthracis* and *B. cereus* which are recognized as human pathogens, along with the well-known *B. thuringiensis*, widely used as a biological control agent. These distinctions highlight the importance of accurate classification within this group, particularly given its relevance to public health, medicine, and agriculture. Liu et al. (2015) demonstrated the utility of whole-genome sequence-based Genome BLAST Distance Phylogeny (GBDP) approach to determine taxonomic affiliations of 224 strains within the *B. cereus* group, identifying 11 known and 19–20 putative novel species. Their findings highlighted the pressing need to re-evaluate the classification and biosafety of strains within this group. Moreover, their study revealed the potential for misclassifications, where *B. anthracis* was incorrectly identified as *B. cereus* or *B. thuringiensis*. Extending this line of inquiry, our study uncovers further misclassifications, including strains labeled as *B. thuringiensis* that are, in fact, *B. anthracis*, the causative agent of anthrax. This misidentification raises serious biosafety concerns, particularly when such strains are employed in biological control programs under the assumption that they are harmless *B. thuringiensis* strains. These findings underscore the urgent need for rigorous taxonomic and biosafety reassessment to mitigate potential risks to public health and environmental safety.

By October 2023, 885 bacterial strains were labeled as *B. thuringiensis* in the NCBI database. After downloading the genomes and phylotaxonomic analyses, it was concluded that out of 885 strains 803 strains were labeled with the correct name. However, 82 strains did not belong to the species *B. thuringiensis* because of low ANI and dDDH similarities between the type strain of the species and those strains. To deal with this taxonomic problem, for the strains mentioned in Supplementary Table S3, we suggest changing the classification from the species *B. thuringiensis* to the species *B. anthracis*. The ANI and dDDH calculations showed that the latter strains had ANI less than 92% and dDDH less than 45% with the type strain of *B. thuringiensis*, while those strains had more than 97 and 77% similarities in ANI and dDDH values with *B. anthracis* type strain, respectively (Supplementary Table S3).

Among Gram-positive bacteria, *Bacillus thuringiensis* is well known for its beneficial features in agriculture. However, other members of Gram-positive bacteria are economically important plant pathogens which are subjected to strict quarantine rules and inspections (Osdaghi et al., 2020a, 2022; Haghverdi et al., 2024). Hence, precise identification and taxonomic delineation of the latter bacteria is also of high importance in terms of plant health and international food trade. For instance, it has recently been noted that plant pathogenic members of *Clavibacter* sp. and *Curtobacterium* sp. needed to be taxonomically refined to meet the current criteria in bacterial taxonomy (Osdaghi et al., 2020b, 2024). This would ease decision making in plant inspections and quarantine ports. Fundamentals of taxonomic studies are similar in both Gram-negative and Gram-positive bacteria. However, sight modifications and adjustments might be proposed for certain groups of bacteria to increase the preciseness and accuracy of the analyses. The 95% ANI threshold was adopted in this study; since, the investigated strains were not only compared to type strain of *B. thuringiensis* but also with all type strains of species within the *Bacillus* genus. The threshold of 96.2% ANI proposed by Liu et al. (2017b) for the *B. cereus* group was not employed because the strains in our study were compared not only with the type strain of *B. thuringiensis*, which belongs to the *B. cereus* group but also with type strains from other species within the *Bacillus* genus that do not belong to the *B. cereus* group. Furthermore, ANI was not used as a sole criteria in this study. Instead, it was combined with dDDH, MLSA-based phylogeny, and core genome-based phylogeny criteria for a more comprehensive species definition. Thus, a strain was classified as belonging to a specific species when both ANI and dDDH exceeded 95 and 70%, respectively, compared to the type strain of that species. This integrated method ensures a more robust classification. Additionally, our observations indicated that for strains within species related to the *Bacillus* genus, when the dDDH value exceeds 70%, the ANI value also tends to exceed 96.6% (data not shown). Therefore, in practice,an ANI threshold of 96.6% was effectively used in these cases, which is close to the threshold suggested by Liu et al. (2017b) for the *B. cereus* group.

However, the 95% ANI threshold is a widely accepted and reliable metric for bacterial species classification, supported by extensive studies validating its efficacy in accurately delineating species boundaries. For instance, Jain et al. (2018) conducted a comprehensive analysis of 90,000 prokaryotic genomes, demonstrating that 99.8% of genome pairs exhibited either >95% ANI within the same species or < 83% ANI across different species, highlighting a clear genetic discontinuity. This robust threshold was consistent across diverse datasets and unaffected by sequencing biases or the predominance of commonly studied species, confirming its universality and applicability. Similarly, Kim et al. (2014) observed a distinct ANI distribution between intra- and interspecies relationships around the 95–96% mark, further reinforcing the validity of the 95% threshold. Moreover, Chan et al. (2012) propose that a combination of core genome phylogenetic analysis and 95% pairwise ANI is an appropriate method for defining bacterial species, providing additional evidence for ANI as a reliable metric. These findings collectively underscore the robustness and utility of the 95% ANI threshold as a foundational criterion for bacterial taxonomy in the genomic era. Even in certain studies, recommendations have been made to lower the ANI threshold below 95% for delineating bacterial species, reflecting the variability in genetic relatedness across different bacterial groups. For instance, Konstantinidis and Tiedje (2005) demonstrated that an ANI of 94% corresponds to the established 70% DNA–DNA reassociation benchmark traditionally used for defining bacterial species, suggesting that a slightly lower threshold can still accurately capture genetic relationships. Similarly, Carroll et al. (2020) investigated the *B. cereus* group and found that the conventional 95% ANI threshold led to overlapping genomospecies clusters, with many genomes belonging to multiple clusters. They proposed a new threshold of approximately 92.5% ANI, which better aligns with natural genome similarity gaps within this group, resulting in more distinct and minimally overlapping genomospecies clusters. Moreover, the study introduced a taxonomic nomenclature framework that integrates genomic species definitions with clinically and industrially significant phenotypes, emphasizing the need for adaptable ANI thresholds to reflect specific genomic and phenotypic contexts.

Richter and Rosselló-Móra (2009) proposed ANI as a superior alternative to traditional DNA–DNA hybridization (DDH), recommending a threshold of 95–96% for species definition. Our study aligns with this approach, employing ANI and dDDH thresholds to evaluate the taxonomic status of the studied *B. thuringiensis* strains. Similarly, Konstantinidis and Tiedje (2005) validated the use of ANI coupled with DDH values, demonstrating that an ANI threshold of 94% corresponds to the traditional 70% DNA reassociation benchmark. While their findings emphasize the complementary use of ANI and DDH, our study extends this framework by incorporating phylogenetic methods, including MLSA and core genome phylogenetic trees. These methods not only confirmed the divergence of the misclassified strains but also added resolution to taxonomic relationships within the *B. cereus* group. Chan et al. (2012) recommended combining ANI with core genome phylogenetic analysis for precise species delineation, which strongly aligns with our methodology. In our study, the integration of core genome analyses with ANI and dDDH enhanced the accuracy of reclassification, demonstrating the necessity of multiple complementary approaches in resolving complex taxonomic challenges. Conversely, Palmer et al. (2020) emphasized the variability in genetic diversity across taxa, advocating for the use of adaptive thresholds and supplementary methods. Our findings align with this perspective, as phylogenetic analyses of misclassified strains consistently revealed distinct clustering patterns in both MLSA- and core genome-based trees. This highlights the importance of employing diverse tools to achieve a comprehensive and accurate species classification. Furthermore, Goris et al. (2007) emphasized the strong correlation between ANI (95%) and DDH (70%) thresholds, advocating their combined application for bacterial species classification. Our results are consistent with this integrative approach, as misclassified strains, including those reclassified as *B. anthracis*, *B. toyonensis*, and other species, exhibited ANI and dDDH values below these established thresholds. In summary, while previous studies primarily focused on ANI and DDH, This study highlights the value of combining ANI, DDH, and phylogenetic analyses to improve bacterial taxonomy, particularly within genetically diverse groups like the *B. cereus* complex.

For the strains MC28, H3, GOE7, GOE5, Bto-UNVM_94, we suggest changing their classification from *B. thuringiensis* to *B. toyonensis* because of their low ANI/dDDH similarity with type strain of the former species (ANI less than 92% and dDDH less than 45%). Whereas they show high similarity (ANI more than 98% and dDDH more than 87%) with the *B. toyonensis* type strain, as shown in Table 2. We propose reclassification of many other strains mislabeled as *B. thuringiensis* as follows: the strains NRRL B23152, BGSC 4Y1, KB1 and XL6 to *B. tropicus* (Table 3), the strain Et10/1 to *B. licheniformis*, BGSC 4BM1 to *B. mycoides*, 4I3 to *B. paranthracis*, BGSC 4BV1 to *B. weidmannii*, and KF1 to *B. basilensis*. In all cases, ANI and dDDH values for those strains in comparison with the *B. thuringiensis* type strain were below the accepted threshold for prokaryotic species description (ANI < 95 and dDDH<70).

On the other hand, there were 35 strains, when compared to all type strains of the genus *Bacillus*, showed a similarity below the acceptable threshold for prokaryotic species definition (ANI < 95, dDDH<70). According to Kumar et al. (2016), when strains did not show similarity higher than the acceptable limit for species definition with all type strains of a particular genus, those strains are likely to be new species. Therefore, we suggest reviewing the classification of the strains mentioned in Table 1. The four strains BGSC 4BY1, IEBC_T61001, BGSC 4BJ1, and NRRL B-23139 were confirmed to relate to *Bacillus pretiosus*, Furthermore, the type strain of *B. pretiosus* SAICEU11^T^ was found to have no significant genomic similarity with the other type strains in the genus *Bacillus*. The species is still listed as having an invalid name, while our findings indicate that its validity as a species should be acknowledged based on genomic evidence, and we propose to consider it an independent species with a reliable name (Figure 2; Supplementary Figure S3).

Through our study and detailed comparative analyses, we concluded that there were no instances where a strain exhibited a dDDH value greater than 70% while simultaneously having an ANI value lower than 95% when compared to the type strain of *B. thuringiensis* or other type strains within the genus *Bacillus*. For all species within the genus *Bacillus*, a dDDH value exceeding 70% typically requires an ANI of 96.6% or higher. This specific finding is a unique outcome of our study and reflects the relationship between these metrics based on our analyzed cases. Conversely, we identified instances where strains had ANI values higher than 95% but dDDH values below 70%. However, these cases were disregarded as they did not simultaneously meet both criteria. This observation was not limited to comparisons involving the type strain of *B. thuringiensis* alone but was also consistent when comparing misclassified strains with the type strains of the other species within the genus *Bacillus*. For example, strain Et10/1 exhibited an ANI value of 95.02% and a dDDH value of 59.10% when compared to the type strain of *B. anthracis*. Thus, it was not classified as belonging to *B. anthracis*. Further comparisons to determine the species to which it belongs revealed that it is a member of *B. licheniformis*, with an ANI value of 99.81% and a dDDH value of 97.60%. This example highlights the utility of comprehensive genomic analyses combined with standard criteria in achieving precise taxonomic classifications across species within this genus.

The question that arises here is why these 82 strains were classified as *B. thuringiensis*, despite their chromosomal similarity to the type strain of the species being minimal and insufficient to consider them part of the species. The main reason for this misclassification is the reliance on the ability of these strains to produce crystal protein, which is commonly used as a Bio-insecticide against various insect pests, as a defining feature of *B. thuringiensis* strains (Bravo et al., 2013). It has been shown that the gene responsible for encoding crystal protein is, in most cases, located on a plasmid rather than the chromosome. Plasmids are susceptible to transfer between closely related bacteria through a process known as conjugation (González et al., 1982). This has been observed in several strains such as HSY204, Bt Gxmzu777-1, and YBT020. For instance, in the strain HSY204 which shows low similarity (ANI = 91.6, dDDH = 44.2) to *B. thuringiensis* type strain, the bacterium contains four plasmids, with the PB plasmid harboring the gene encoding the insecticidal delta-endotoxin Cry8Ea1. Whereas, on the pC plasmid, there is a gene encoding the insecticidal crystal protein Cry1Ac. When this strain was first isolated, it was described as a bio-insecticide effective against *Aedes aegypti* larvae (Wu et al., 2021).

The strain Bt Gxmzu777-1 has been identified as a bacterium belonging to *B. thuringiensis* despite the very low similarity (ANI = 89.9, dDDH = 38.2) of this strain to type strain of *B. thuringiensis* (data not shown). This might be due in part to the fact that this strain possesses four plasmids, where two of these plasmids, i.e., pa (262,245 bp) and pd. (259,656 bp) containing the gene encoding the insecticidal delta-endotoxin Cry8Ea1 family protein. The strain YBT-020 possesses two plasmids, and on plasmid pBMB26 (187,880 pb), there are genes encoding the pesticidal crystal proteins *cry*4Ba and *cry*4Aa, while on plasmid PBMB28 there is gene *cry*4Aa. In addition, it is likely that the misclassification of the three strains (TYU3, TYU4, JJ1873) as members of the *B. toyonensis* species despite their phylogenetic relation to *B. thuringiensis*, stems from the absence of crystalline protein production in these strains (data not shown).

All this evidence emphasizes that the fundamental principle when working on the classification of *B. thuringiensis* strains is the production of crystal proteins known for their insecticidal activity. This may lead to inaccurate classification and errors in identification. Therefore, it is essential to strive for the use of more reliable and precise standards, such as those based on chromosomal comparison (calculating dDDH and ANI) and relying on traits encoded by genes present on the chromosome to differentiate between strains, which are less prone to loss or transfer between bacterial species. The important result that was reached through this study is the necessity of searching for modern and approved methods to classify species belonging to the genus *Bacillus*, and in particular, species belonging to the *B. cereus* group, which are very similar genetically while very different in terms of phenotypic traits. One of the reliable methods is to use a biomarker specific to the species to be classified. For example, in the case of *B. thuringiensis*, the gene coding for the transcriptional regulator of insecticidal toxin (*xre*), xenobiotic response element is commonly observed in strains that are classified as *B. thuringiensis* and was used as a biomarker by Wei et al. (2019) to distinguish the mentioned species from its close species in the *B. cereus* group. Out of the 33 mislabeled *B. thuringiensis* strains, which should be classified as *B. anthracis*, some strains do not contain *xre* gene like FDAARGOS_792, 97–27, BM-BT15426, HD682, FDAARGOS_794, HD1011, FDAARGOS_795, 4XX3, 4XX2, 4XX1, 4 W2, BGSC 4BX1, Patient-SAMN36761919, FDAARGOS_793, Al Hakam, HD571, FDAARGOS_791, and 4B3. Since the strains belonging to *B. thuringiensis* contain the *xre* gene in their genomes and the mentioned *B. anthracis* strains do not have that gene, this supports our hypothesis that these strains do not belong to *B. thuringiensis* species (data not shown). Another example is PLCR-regulated proteins. In *B. thuringiensis* PLCR is a pleiotropic regulator of extracellular virulence factor gene expression (Lereclus and Agaisse, 2000). None of the 33 strains in this study that must be reclassified as *B. anthracis* has this protein. This further confirms the validity of the hypothesis that these strains not only do not belong to *B. thuringiensis* but also belong to the *B. anthracis* species, because the characteristic of the *B. anthracis* strains is that they do not contain PLCR-regulated proteins PRP2 and PLCR gene is truncated (Lereclus et al., 2000). Instead, the essential virulence factors such as capsule formation and anthrax toxin are regulated by regulatory genes, *acpA* and *atxA* located on plasmids pXO2 and pXO2, respectively, (Bourgogne et al., 2003).

In conclusion, the outcomes of this study are anticipated to enhance the safety and precision of biological control applications of *B. thuringiensis* strains by ensuring accurate identification and taxonomic delineation. Given the coexistence of *B. thuringiensis* with human pathogenic species like *B. cereus* and *B. anthracis*, the latter possessing a significant biosecurity threat, the refined classification approach outlined here will facilitate reliable selection of strains suitable for biological control. Finally, MLSA-based taxonomic framework drawn in this study would facilitate identification of suspected *B. thuringiensis* strains in the coming works. These findings underscore the importance of integrating genomic tools for the precise classification of strains to minimize risks to the environment and human health while optimizing the use of microbial agents in agriculture.

## Data Availability

The original contributions presented in the study are included in the article/Supplementary material, further inquiries can be directed to the corresponding author.

## Footnotes

^1^ https://usegalaxy.eu/

^2^ https://mafft.cbrc.jp/alignment/server/

^3^ https://lpsn.dsmz.de/

^4^ https://tygs.dsmz.de/

## Supplementary material

SUPPLEMENTARY FIGURE S1 MLSA-based phylogenetic tree using the sequences of five housekeeping genes *gyrB*, *atpD*, *dnaK*, *rpoB*, and *rpoD* for all *Bacillus thuringiensis* strains retrieved from the NCBI database in this study.

SUPPLEMENTARY FIGURE S2 Phylogenetic tree inferred with FastME 2.1.6.1 (Lefort et al., 2015) of 46 mislabeled *Bacillus thuringiensis* strains belonging to different species of the genus *Bacillus* from GBDP distances calculated from genome sequences. The branch lengths are scaled in terms of GBDP distance formula d5. The numbers above branches are GBDP pseudo-bootstrap support values >60% from 100 replications, with an average branch support of 38.4%. The tree was rooted at the midpoint (Farris, 1972).

SUPPLEMENTARY FIGURE S3 Phylogenetic tree inferred with FastME 2.1.6.1 (Lefort et al., 2015) of the 35 mislabeled *Bacillus thuringiensis* strains not belonging to any species generated from GBDP distances calculated of genome sequences. The branch lengths are scaled in terms of GBDP distance formula d5. The numbers above branches are GBDP pseudo-bootstrap support values >60% from 100 replications, with an average branch support of 49.7%. The tree was rooted at the midpoint (Farris, 1972).

SUPPLEMENTARY TABLE S1 List of 885 strains labeled as *Bacillus thuringiensis* retrieved from the NCBI GenBank database.

SUPPLEMENTARY TABLE S2 List of type strains of all validly described *Bacillus* species used in the study according to the LPSN database https://lpsn.dsmz.de/.

SUPPLEMENTARY TABLE S3 Digital DNA–DNA hybridization (dDDH; upper diagonal) and average nucleotide identity (ANIm; lower diagonal) values generated from the DNA sequence similarity comparisons among different mislabeled *Bacillus thuringiensis* strains and the *Bacillus anthracis* type strain.
