# Supplementary figures and images for: Taxonomic refinement of Bacillus thuringiensis
Source: Front Microbiol. 2025 Feb 7;16:1518307. doi: 10.3389/fmicb.2025.1518307 (PMC11843730; doi:10.3389/fmicb.2025.1518307)

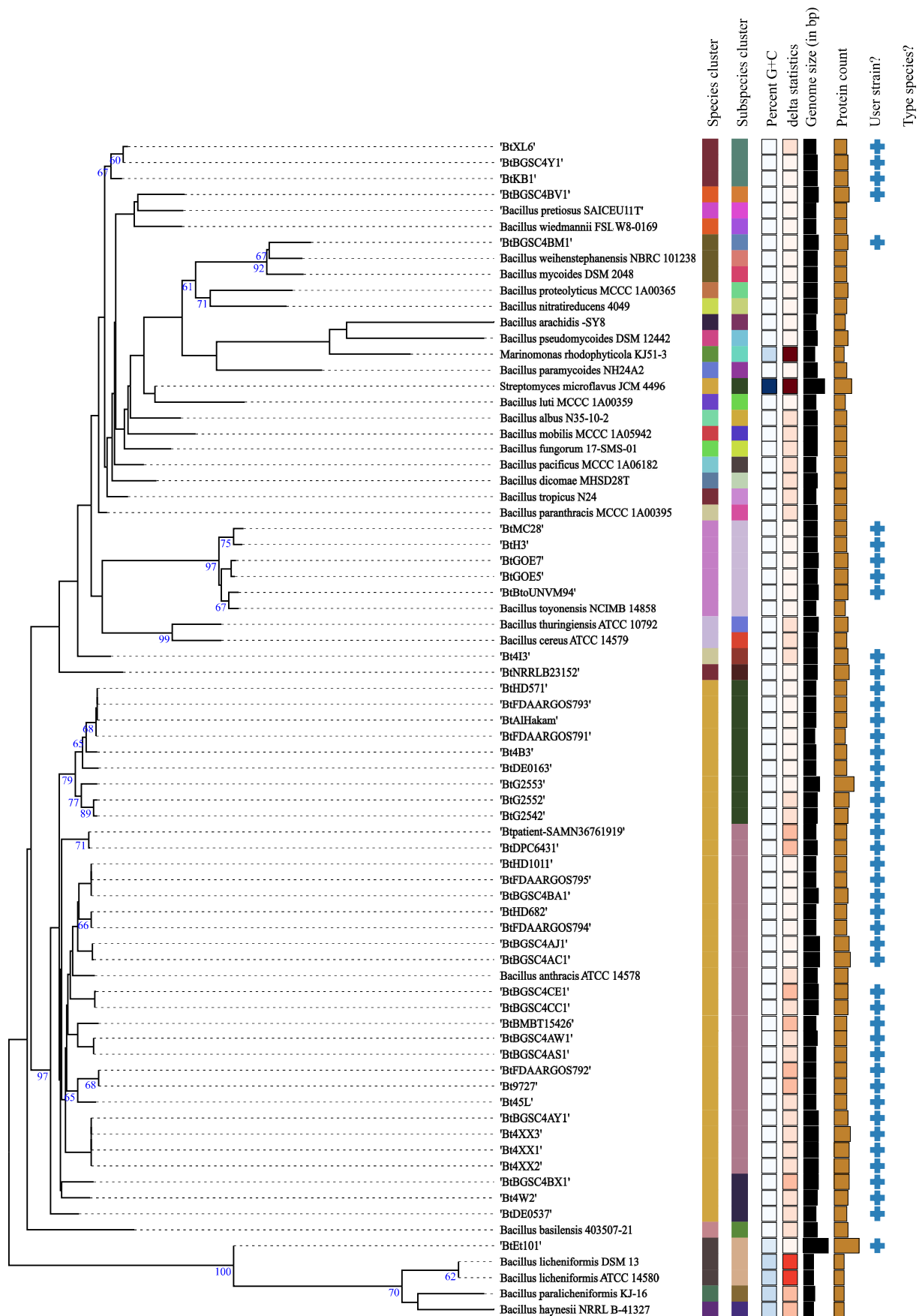

Supplement: SUPPLEMENTARY FIGURE S2 — Phylogenetic tree inferred with FastME 2.1.6.1 (Lefort et al., 2015) of 46 mislabeled Bacillus thuringiensis strains belonging to different species of the genus Bacillus from GBDP distances calculated from genome sequences. The branch lengths are scaled in terms of GBDP distance formula d5. The numbers above branches are GBDP pseudo-bootstrap support values >60% from 100 replications, with an average branch support of 38.4%. The tree was rooted at the midpoint (Farris, 1972). [file Data_Sheet_2.pdf]

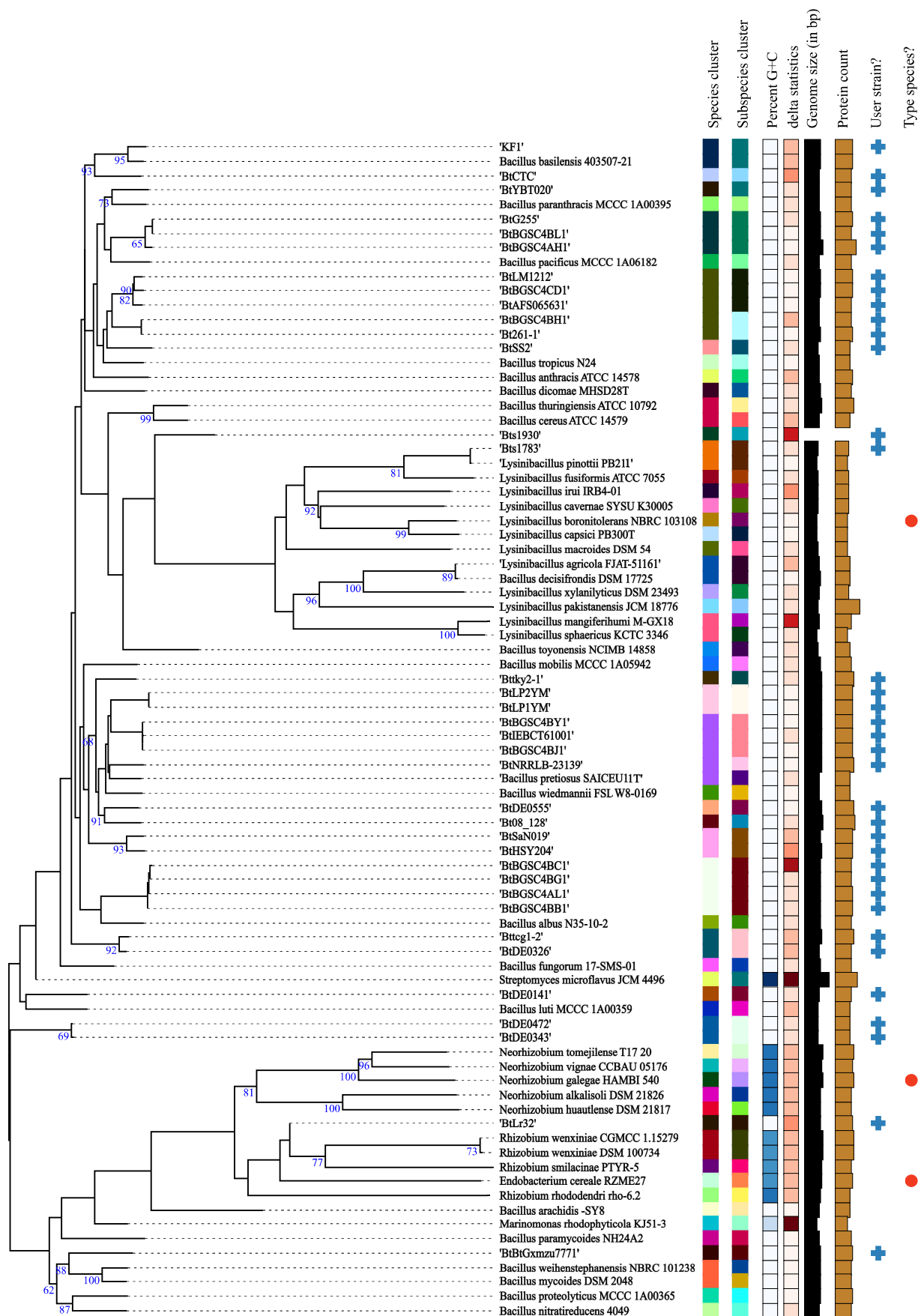

Supplement: SUPPLEMENTARY FIGURE S3 — Phylogenetic tree inferred with FastME 2.1.6.1 (Lefort et al., 2015) of the 35 mislabeled Bacillus thuringiensis strains not belonging to any species generated from GBDP distances calculated of genome sequences. The branch lengths are scaled in terms of GBDP distance formula d5. The numbers above branches are GBDP pseudo-bootstrap support values >60% from 100 replications, with an average branch support of 49.7%. The tree was rooted at the midpoint (Farris, 1972). [file Data_Sheet_3.pdf]
