# Supplementary material for: Taxonomic refinement of Bacillus thuringiensis
Source: Front Microbiol. 2025 Feb 7;16:1518307. doi: 10.3389/fmicb.2025.1518307 (PMC11843730; doi:10.3389/fmicb.2025.1518307)
Supplement: SUPPLEMENTARY TABLE S1 — List of 885 strains labeled as Bacillus thuringiensis retrieved from the NCBI GenBank database. [file Table_1.docx]

Table S1: The studied *Bacillus thuringiensis* ‎ strains, their geographic origin, isolation source, year of isolation, and references.

|  | **Taxon** | **strain** | **Host of isolation or**  **isolation_source** | **GenBank accession number** | **Geographic origin** | **Year of isolation** | **References** |
| --- | --- | --- | --- | --- | --- | --- | --- |
|  | *B. thuringiensis* | 1126_1 | soil | NHPK02000044.1 | China | 2017 | Shu (2017) |
|  | *B. thuringiensis* | 1230 | unknown | VIWB01000023.1 | USA | unknown | Schachtman et al. (2019) |
|  | *B. thuringiensis* | 14 | unknown | NKBB01000014.1 | China | 2016 | Cui (2017) |
|  | *B. thuringiensis* | 147 | soil | LFXM01000033.1 | Brazil | 2014 | Bertucci arbosa et al. 2015 |
|  | *B. thuringiensis* | 18_216 | pesticide | JAJBJH010000045.1 | France | 2018 | Biggel (2021) |
|  | *B. thuringiensis* | 18_218 | pesticide | JAJBJF010000041.1 | France | 2018 | Biggel (2021) |
|  | *B. thuringiensis* | 18_421 | pesticide | JAJBJG010000037.1 | France | 2018 | Biggel (2021) |
|  | *B. thuringiensis* | 18_483 | pesticide | JAJBJJ010000029.1 | France | 2018 | Biggel (2021) |
|  | *B. thuringiensis* | 18_484 | pesticide | JAJBJK010000030.1 | France | 2018 | Biggel (2021) |
|  | *B. thuringiensis* | 18_614 | pesticide | JAJBJI010000043.1 | France | 2018 | Biggel (2021) |
|  | *B. thuringiensis* | 20-1 | unknown | NKAW01000034.1 | China | 2016 | Cui (2017) |
|  | *B. thuringiensis* | 21-1 | unknown | NKBA01000013.1 | China | 2016 | Cui (2017) |
|  | *B. thuringiensis* | 25-6 | unknown | NKAZ01000084.1 | China | 2016 | Cui (2017) |
|  | *B. thuringiensis* | 26-1 | unknown | NKAV01000115.1 | China | 2016 | Cui (2017) |
|  | *B. thuringiensis* | 261-1 | soil | NHNP01000011.1 | China | 2003 | Shu (2017) |
|  | *B. thuringiensis* | 26-4 | unknown | NKAU01000014.1 | China | 2016 | Cui (2017) |
|  | *B. thuringiensis* | 27 | Planococcus citri | JWJY01000040.1 | Egypt | 2010 | Rusconi et al. (2014) |
|  | *B. thuringiensis* | 27-3 | unknown | NKAT01000048.1 | China | 2016 | Cui (2017) |
|  | *B. thuringiensis* | 30-1 | unknown | NKAS01000018.1 | China | 2016 | Cui (2017) |
|  | *B. thuringiensis* | 30-4 | unknown | NKAR01000035.1 | China | 2016 | Cui (2017) |
|  | *B. thuringiensis* | 407 | unknown | CP003889.1 | unknown | unknown | Sheppard et al. (2013) |
|  | *B. thuringiensis* | 45L | leaf | JAQOOH010000010.1 | Bangladesh | Bangladesh | Hasan et al. (2023) |
|  | *B. thuringiensis* | 4A4 | unknown | DAOJKK010000006.1 | unknown | unknown | Souvorov et al. (2018) |
|  | *B. thuringiensis* | 4AA1 | Tribolium castaneum | CP015310.1 | unknown | unknown | Hollensteiner et al. (2016) |
|  | *B. thuringiensis* | 4B3 | unknown | WJDN01000001.1 | unknown | unknown | Shu (2019) |
|  | *B. thuringiensis* | 4F5 | BGSC database | CP106947 | China | 2021 | Zhang et al. (2022) |
|  | *B. thuringiensis* | 4I3 | unknown | WJCV01000001.1 | unknown | unknown | Shu (2019) |
|  | *B. thuringiensis* | 4Q7 | soil | JEOC01000018.1 | Israel | 1976 | Jeong and Choi (2014) |
|  | *B. thuringiensis* | 4W2 | unknown | WJCD01000001 | unknown | unknown | Shu (2019) |
|  | *B. thuringiensis* | 4XX1 | unknown | WJCC01000016.1 | unknown | unknown | Shu (2019) |
|  | *B. thuringiensis* | 4XX2 | unknown | WJCB01000001.1 | unknown | unknown | Shu (2019) |
|  | *B. thuringiensis* | 4XX3 | unknown | WJCA01000001 | unknown | unknown | Shu (2019) |
|  | *B. thuringiensis* | 7.1o | Bombyx mori | PGEH01000346.1 | United Kingdom | 2013 | Pascoe et al. (2017) |
|  | *B. thuringiensis* | 78-2 | soil | NHNO01000002.1 | China | 2003 | Shu (2017) |
|  | *B. thuringiensis* | 871Blist1 | surface of a grape leaf | JADCKT010000008.1 | Russia | 2019 | Bryanskaya et al. (2020) |
|  | *B. thuringiensis* | AATZO | Homo sapiens | JAJHQA010000028.1 | USA | 2013 | Segre and Mullikin (2021) |
|  | *B. thuringiensis* | ABTS_1857 | pesticide | DAOIXK010000039.1 | France | 2018 | Souvorov et al. (2018) |
|  | *B. thuringiensis* | ABTS_1857 | pesticide | DAOJAR010000038.1 | France | 2018 | Souvorov et al. (2018) |
|  | *B. thuringiensis* | ABTS-1857 | Biopesticide XenTari | CP083156 | unknown | 2017 | Biggel (2021) |
|  | *B. thuringiensis* | ABTS-351 | Biopesticide DiPel D | CP083101.1 | unknown | 2017 | Biggel (2021) |
|  | *B. thuringiensis* | AFS000517 | Soybean | NTZW01000012.1 | USA | 2014 | Bleich et al. (2017) |
|  | *B. thuringiensis* | AFS001991 | corn | NTZK01000023.1 | USA | 2014 | Bleich et al. (2017) |
|  | *B. thuringiensis* | AFS002494 | corn | NTZD01000048.1 | USA | 2014 | Bleich et al. (2017) |
|  | *B. thuringiensis* | AFS002866 | Soybean | NTYY01000051.1 | USA | 2014 | Bleich et al. (2017) |
|  | *B. thuringiensis* | AFS002920 | Soybean | NTYX01000005.1 | USA | 2014 | Bleich et al. (2017) |
|  | *B. thuringiensis* | AFS003097 | plant | NTYV01000018.1 | USA | 2014 | Bleich et al. (2017) |
|  | *B. thuringiensis* | AFS004356 | corn | NTYO01000031.1 | USA | 2014 | Bleich et al. (2017) |
|  | *B. thuringiensis* | AFS005011 | Weed | NTYI01000076.1 | USA | 2014 | Bleich et al. (2017) |
|  | *B. thuringiensis* | AFS005023 | plant | NTYH01000010.1 | USA | 2014 | Bleich et al. (2017) |
|  | *B. thuringiensis* | AFS005083 | leaf | NTYG01000010.1 | USA | 2014 | Bleich et al. (2017) |
|  | *B. thuringiensis* | AFS005140 | corn | NTYF01000015.1 | USA | 2014 | Bleich et al. (2017) |
|  | *B. thuringiensis* | AFS006689 | Soybean | NTXT01000049.1 | USA | 2014 | Bleich et al. (2017) |
|  | *B. thuringiensis* | AFS006923 | soil | NTXR01000001.1 | USA | 2014 | Bleich et al. (2017) |
|  | *B. thuringiensis* | AFS007186 | corn | NTXO01000049.1 | USA | 2014 | Bleich et al. (2017) |
|  | *B. thuringiensis* | AFS007900 | Soybean | NTXF01000060.1 | USA | 2014 | Bleich et al. (2017) |
|  | *B. thuringiensis* | AFS009218 | corn | NTWT01000031.1 | USA | 2014 | Bleich et al. (2017) |
|  | *B. thuringiensis* | AFS009253 | tree leaf | NTWS01000051.1 | USA | 2014 | Bleich et al. (2017) |
|  | *B. thuringiensis* | AFS009644 | tree leaf | NTWN01000018.1 | USA | 2014 | Bleich et al. (2017) |
|  | *B. thuringiensis* | AFS009835 | soil | NTWJ01000018.1 | USA | 2014 | Bleich et al. (2017) |
|  | *B. thuringiensis* | AFS010823 | Soybean | NTWD01000005.1 | USA | 2014 | Bleich et al. (2017) |
|  | *B. thuringiensis* | AFS011084 | Soybean | NTVZ01000012.1 | USA | 2014 | Bleich et al. (2017) |
|  | *B. thuringiensis* | AFS011659 | plant core | NTVW01000045.1 | USA | 2014 | Bleich et al. (2017) |
|  | *B. thuringiensis* | AFS011673 | Soybean | NTVV01000038.1 | USA | 2013 | Bleich et al. (2017) |
|  | *B. thuringiensis* | AFS011848 | Soybean | NTVU01000015.1 | USA | 2013 | Bleich et al. (2017) |
|  | *B. thuringiensis* | AFS011939 | Soybean | NTVS01000012.1 | USA | 2013 | Bleich et al. (2017) |
|  | *B. thuringiensis* | AFS012085 | corn | NTVQ01000082.1 | USA | 2013 | Bleich et al. (2017) |
|  | *B. thuringiensis* | AFS012232 | Weed | NTVP01000069.1 | USA | 2013 | Bleich et al. (2017) |
|  | *B. thuringiensis* | AFS013213 | corn | NTVJ01000039.1 | USA | 2013 | Bleich et al. (2017) |
|  | *B. thuringiensis* | AFS014145 | Soybean | NTVC01000064.1 | USA | 2013 | Bleich et al. (2017) |
|  | *B. thuringiensis* | AFS014334 | Soybean | NTVB01000067.1 | USA | 2014 | Bleich et al. (2017) |
|  | *B. thuringiensis* | AFS015413 | corn | NTUS01000040.1 | USA | 2014 | Bleich et al. (2017) |
|  | *B. thuringiensis* | AFS015432 | soil | NTUR01000023.1 | USA | 2014 | Bleich et al. (2017) |
|  | *B. thuringiensis* | AFS016611 | Soybean | NTUJ01000002.1 | USA | 2014 | Bleich et al. (2017) |
|  | *B. thuringiensis* | AFS016894 | soil | NTUH01000055.1 | USA | 2014 | Bleich et al. (2017) |
|  | *B. thuringiensis* | AFS016919 | plant core | NTUF01000021.1 | USA | 2014 | Bleich et al. (2017) |
|  | *B. thuringiensis* | AFS017371 | unknown | NTTX01000030.1 | USA | 2014 | Bleich et al. (2017) |
|  | *B. thuringiensis* | AFS018179 | Corn | NTTR01000050.1 | USA | 2013 | Bleich et al. (2017) |
|  | *B. thuringiensis* | AFS018331 | plant core | NTTO01000075.1 | USA | 2014 | Bleich et al. (2017) |
|  | *B. thuringiensis* | AFS018409 | Oats | NUCJ01000147.1 | USA | 2014 | Bleich et al. (2017) |
|  | *B. thuringiensis* | AFS018617 | soil | NTTJ01000025.1 | USA | 2014 | Bleich et al. (2017) |
|  | *B. thuringiensis* | AFS019518 | Soybean | NTTG01000047.1 | USA | 2014 | Bleich et al. (2017) |
|  | *B. thuringiensis* | AFS019545 | plant core | NTTF01000029.1 | USA | 2014 | Bleich et al. (2017) |
|  | *B. thuringiensis* | AFS019758 | Soybean | NTTD01000046.1 | USA | 2014 | Bleich et al. (2017) |
|  | *B. thuringiensis* | AFS020128 | Soybean | NTSP01000037.1 | USA | 2013 | Bleich et al. (2017) |
|  | *B. thuringiensis* | AFS020602 | soil | NTSL01000018.1 | USA | 2014 | Bleich et al. (2017) |
|  | *B. thuringiensis* | AFS020608 | Corn | NTSK01000053.1 | USA | 2014 | Bleich et al. (2017) |
|  | *B. thuringiensis* | AFS021634 | Soybean | NTSF01000044.1 | USA | 2014 | Bleich et al. (2017) |
|  | *B. thuringiensis* | AFS021667 | Corn | NTSE01000069.1 | USA | 2014 | Bleich et al. (2017) |
|  | *B. thuringiensis* | AFS022028 | Soybean | NTSD01000030.1 | USA | 2014 | Bleich et al. (2017) |
|  | *B. thuringiensis* | AFS022064 | Corn | NTSC01000018.1 | USA | 2014 | Bleich et al. (2017) |
|  | *B. thuringiensis* | AFS022086 | soil | NTSB01000030.1 | USA | 2014 | Bleich et al. (2017) |
|  | *B. thuringiensis* | AFS022504 | Corn | NTRU01000048.1 | USA | 2014 | Bleich et al. (2017) |
|  | *B. thuringiensis* | AFS022658 | Soybean | NTRS01000060.1 | USA | 2014 | Bleich et al. (2017) |
|  | *B. thuringiensis* | AFS023096 | Soybean | NTRP01000099.1 | USA | 2014 | Bleich et al. (2017) |
|  | *B. thuringiensis* | AFS023418 | Soybean | NTRM01000008.1 | USA | 2014 | Bleich et al. (2017) |
|  | *B. thuringiensis* | AFS023506 | Soybean | NTRJ01000031.1 | USA | 2014 | Bleich et al. (2017) |
|  | *B. thuringiensis* | AFS023641 | plant core | NTRI01000007.1 | USA | 2014 | Bleich et al. (2017) |
|  | *B. thuringiensis* | AFS024266 | plant core | NTRD01000044.1 | USA | 2014 | Bleich et al. (2017) |
|  | *B. thuringiensis* | AFS024615 | plant core | NTRA01000020.1 | USA | 2014 | Bleich et al. (2017) |
|  | *B. thuringiensis* | AFS025510 | Soil | NTQO01000078.1 | USA | 2014 | Bleich et al. (2017) |
|  | *B. thuringiensis* | AFS025854 | Soybean | NTQM01000003.1 | USA | 2014 | Bleich et al. (2017) |
|  | *B. thuringiensis* | AFS026252 | corn | NTQK01000221.1 | USA | 2014 | Bleich et al. (2017) |
|  | *B. thuringiensis* | AFS026926 | soil | NTQF01000053.1 | USA | 2014 | Bleich et al. (2017) |
|  | *B. thuringiensis* | AFS027178 | Soybean | NTQE01000058.1 | USA | 2014 | Bleich et al. (2017) |
|  | *B. thuringiensis* | AFS027250 | corn | NTQD01000045.1 | USA | 2014 | Bleich et al. (2017) |
|  | *B. thuringiensis* | AFS029475 | Corn | NTSS01000028.1 | USA | 2014 | Bleich et al. (2017) |
|  | *B. thuringiensis* | AFS029508 | Soybean | NTSR01000038.1 | USA | 2014 | Bleich et al. (2017) |
|  | *B. thuringiensis* | AFS029767 | Soybean | NUPW01000018.1 | USA | 2014 | Bleich et al. (2017) |
|  | *B. thuringiensis* | AFS029963 | plant core | NUPT01000021.1 | USA | 2014 | Bleich et al. (2017) |
|  | *B. thuringiensis* | AFS030095 | Soybean | NUPO01000042.1 | USA | 2014 | Bleich et al. (2017) |
|  | *B. thuringiensis* | AFS030179 | corn | NUPM01000010.1 | USA | 2014 | Bleich et al. (2017) |
|  | *B. thuringiensis* | AFS030866 | Soybean | NUPG01000027.1 | USA | 2014 | Bleich et al. (2017) |
|  | *B. thuringiensis* | AFS030961 | Soybean | NUPE01000032.1 | USA | 2014 | Bleich et al. (2017) |
|  | *B. thuringiensis* | AFS031210 | plant core | NUPB01000108.1 | USA | 2014 | Bleich et al. (2017) |
|  | *B. thuringiensis* | AFS031473 | Oats | NUUL01000119.1 | USA | 2014 | Bleich et al. (2017) |
|  | *B. thuringiensis* | AFS031631 | corn | NUOV01000040.1 | USA | 2013 | Bleich et al. (2017) |
|  | *B. thuringiensis* | AFS031651 | Beetle | NUOU01000006.1 | USA | 2013 | Bleich et al. (2017) |
|  | *B. thuringiensis* | AFS031814 | soil | NUOS01000035.1 | USA | 2014 | Bleich et al. (2017) |
|  | *B. thuringiensis* | AFS032723 | Soybean | NUOL01000015.1 | USA | 2014 | Bleich et al. (2017) |
|  | *B. thuringiensis* | AFS033597 | soil | NUOC01000058.1 | USA | 2014 | Bleich et al. (2017) |
|  | *B. thuringiensis* | AFS034084 | Wheat | NUTX01000060.1 | USA | 2014 | Bleich et al. (2017) |
|  | *B. thuringiensis* | AFS034126 | corn | NUOA01000006.1 | USA | 2014 | Bleich et al. (2017) |
|  | *B. thuringiensis* | AFS034546 | Soybean | NUNW01000065.1 | USA | 2014 | Bleich et al. (2017) |
|  | *B. thuringiensis* | AFS034600 | plant core | NUNV01000009.1 | USA | 2014 | Bleich et al. (2017) |
|  | *B. thuringiensis* | AFS034708 | plant core | NUNU01000038.1 | USA | 2014 | Bleich et al. (2017) |
|  | *B. thuringiensis* | AFS034818 | plant core | NUNR01000016.1 | USA | 2014 | Bleich et al. (2017) |
|  | *B. thuringiensis* | AFS036336 | plant core | NUNL01000008.1 | USA | 2014 | Bleich et al. (2017) |
|  | *B. thuringiensis* | AFS036350 | corn | NUNK01000050.1 | USA | 2014 | Bleich et al. (2017) |
|  | *B. thuringiensis* | AFS036383 | Soybean | NUNI01000020.1 | USA | 2014 | Bleich et al. (2017) |
|  | *B. thuringiensis* | AFS036437 | Mushroom | NUNG01000025.1 | USA | 2013 | Bleich et al. (2017) |
|  | *B. thuringiensis* | AFS038158 | Soybean | NUMX01000004.1 | USA | 2013 | Bleich et al. (2017) |
|  | *B. thuringiensis* | AFS039774 | Soybean | NUMM01000053.1 | USA | 2014 | Bleich et al. (2017) |
|  | *B. thuringiensis* | AFS039999 | corn | NUMJ01000026.1 | USA | 2014 | Bleich et al. (2017) |
|  | *B. thuringiensis* | AFS040001 | corn | NUMI01000015.1 | USA | 2014 | Bleich et al. (2017) |
|  | *B. thuringiensis* | AFS040286 | corn | NUMD01000020.1 | USA | 2014 | Bleich et al. (2017) |
|  | *B. thuringiensis* | AFS040392 | grass | NUMA01000038.1 | USA | 2013 | Bleich et al. (2017) |
|  | *B. thuringiensis* | AFS040438 | corn | NULZ01000007.1 | USA | 2014 | Bleich et al. (2017) |
|  | *B. thuringiensis* | AFS040661 | corn | NULW01000006.1 | USA | 2014 | Bleich et al. (2017) |
|  | *B. thuringiensis* | AFS040987 | Soybean | NULS01000001.1 | USA | 2014 | Bleich et al. (2017) |
|  | *B. thuringiensis* | AFS041525 | plant core | NULM01000058.1 | USA | 2014 | Bleich et al. (2017) |
|  | *B. thuringiensis* | AFS042804 | Soybean | NULA01000146.1 | USA | 2014 | Bleich et al. (2017) |
|  | *B. thuringiensis* | AFS042804 | Soybean | NULJ01000038.1 | USA | 2014 | Bleich et al. (2017) |
|  | *B. thuringiensis* | AFS043063 | Soybean | NUKV01000015.1 | USA | 2014 | Bleich et al. (2017) |
|  | *B. thuringiensis* | AFS043211 | plant core | NUKT01000003.1 | USA | 2014 | Bleich et al. (2017) |
|  | *B. thuringiensis* | AFS043402 | corn | NUKR01000033.1 | USA | 2014 | Bleich et al. (2017) |
|  | *B. thuringiensis* | AFS043499 | Soybean | NUKP01000010.1 | USA | 2013 | Bleich et al. (2017) |
|  | *B. thuringiensis* | AFS043555 | soil | NUKN01000077.1 | USA | 2014 | Bleich et al. (2017) |
|  | *B. thuringiensis* | AFS043930 | Oats | NUSR01000163.1 | USA | 2014 | Bleich et al. (2017) |
|  | *B. thuringiensis* | AFS045516 | corn | NUJX01000054.1 | USA | 2014 | Bleich et al. (2017) |
|  | *B. thuringiensis* | AFS045572 | plant core | NUJW01000003.1 | USA | 2014 | Bleich et al. (2017) |
|  | *B. thuringiensis* | AFS045814 | plant | NUJV01000015.1 | USA | 2014 | Bleich et al. (2017) |
|  | *B. thuringiensis* | AFS045876 | plant core | NUJT01000025.1 | USA | 2014 | Bleich et al. (2017) |
|  | *B. thuringiensis* | AFS046081 | Soybean | NUJR01000024.1 | USA | 2014 | Bleich et al. (2017) |
|  | *B. thuringiensis* | AFS046545 | corn | NUJM01000034.1 | USA | 2014 | Bleich et al. (2017) |
|  | *B. thuringiensis* | AFS047495 | corn | NUJG01000041.1 | USA | 2014 | Bleich et al. (2017) |
|  | *B. thuringiensis* | AFS047607 | Soybean | NUJF01000012.1 | USA | 2014 | Bleich et al. (2017) |
|  | *B. thuringiensis* | AFS047635 | Soybean | NUJE01000008.1 | USA | 2014 | Bleich et al. (2017) |
|  | *B. thuringiensis* | AFS048014 | soybean | NUJC01000087.1 | USA | 2014 | Bleich et al. (2017) |
|  | *B. thuringiensis* | AFS048765 | soybean | NUIV01000035.1 | USA | 2014 | Bleich et al. (2017) |
|  | *B. thuringiensis* | AFS049152 | plant core | NUIP01000043.1 | USA | 2014 | Bleich et al. (2017) |
|  | *B. thuringiensis* | AFS049152 | plant core | NUIU01000027.1 | USA | 2014 | Bleich et al. (2017) |
|  | *B. thuringiensis* | AFS049207 | excrement | NUIO01000006.1 | USA | 2014 | Bleich et al. (2017) |
|  | *B. thuringiensis* | AFS050081 | soybean | NUIK01000005.1 | USA | 2014 | Bleich et al. (2017) |
|  | *B. thuringiensis* | AFS050333 | soybean | NUIJ01000034.1 | USA | 2014 | Bleich et al. (2017) |
|  | *B. thuringiensis* | AFS050550 | soybean | NUII01000004.1 | USA | 2014 | Bleich et al. (2017) |
|  | *B. thuringiensis* | AFS052034 | host | NUHZ01000041.1 | USA | 2014 | Bleich et al. (2017) |
|  | *B. thuringiensis* | AFS052460 | soil | NUHW01000065.1 | USA | 2014 | Bleich et al. (2017) |
|  | *B. thuringiensis* | AFS052676 | soybean | NUHU01000013.1 | USA | 2014 | Bleich et al. (2017) |
|  | *B. thuringiensis* | AFS052778 | soil | NUHT01000012.1 | USA | 2014 | Bleich et al. (2017) |
|  | *B. thuringiensis* | AFS052794 | soybean | NUHS01000042.1 | USA | 2014 | Bleich et al. (2017) |
|  | *B. thuringiensis* | AFS053253 | soybean | NUHN01000198.1 | USA | 2014 | Bleich et al. (2017) |
|  | *B. thuringiensis* | AFS053744 | soil | NUHG01000070.1 | USA | 2014 | Bleich et al. (2017) |
|  | *B. thuringiensis* | AFS053994 | soybean | NUHF01000015.1 | USA | 2014 | Bleich et al. (2017) |
|  | *B. thuringiensis* | AFS054059 | corn | NUHD01000027.1 | USA | 2014 | Bleich et al. (2017) |
|  | *B. thuringiensis* | AFS054136 | corn | NUHC01000026.1 | USA | 2013 | Bleich et al. (2017) |
|  | *B. thuringiensis* | AFS054343 | corn | NUGZ01000072.1 | USA | 2014 | Bleich et al. (2017) |
|  | *B. thuringiensis* | AFS054440 | soybean | NUGY01000096.1 | USA | 2014 | Bleich et al. (2017) |
|  | *B. thuringiensis* | AFS054835 | corn | NUGW01000048.1 | USA | 2014 | Bleich et al. (2017) |
|  | *B. thuringiensis* | AFS055002 | corn | NUGU01000020.1 | USA | 2014 | Bleich et al. (2017) |
|  | *B. thuringiensis* | AFS055282 | corn | NUGS01000031.1 | USA | 2014 | Bleich et al. (2017) |
|  | *B. thuringiensis* | AFS055421 | corn | NUGQ01000011.1 | USA | 2014 | Bleich et al. (2017) |
|  | *B. thuringiensis* | AFS055470 | Soybean | NUGP01000025.1 | USA | 2014 | Bleich et al. (2017) |
|  | *B. thuringiensis* | AFS055708 | plant core | NUGL01000022.1 | USA | 2014 | Bleich et al. (2017) |
|  | *B. thuringiensis* | AFS055854 | plant core | NUGK01000013.1 | USA | 2014 | Bleich et al. (2017) |
|  | *B. thuringiensis* | AFS056009 | unknown | NUGJ01000019.1 | USA | 2014 | Bleich et al. (2017) |
|  | *B. thuringiensis* | AFS056280 | plant core | NUGG01000013.1 | USA | 2014 | Bleich et al. (2017) |
|  | *B. thuringiensis* | AFS056597 | soil | NUGD01000006.1 | USA | 2014 | Bleich et al. (2017) |
|  | *B. thuringiensis* | AFS056615 | Soybean | NUGB01000024.1 | USA | 2014 | Bleich et al. (2017) |
|  | *B. thuringiensis* | AFS056670 | corn | NUGA01000124.1 | USA | 2013 | Bleich et al. (2017) |
|  | *B. thuringiensis* | AFS057244 | soil | NUFW01000008.1 | USA | 2014 | Bleich et al. (2017) |
|  | *B. thuringiensis* | AFS057784 | corn | NUFP01000003.1 | USA | 2014 | Bleich et al. (2017) |
|  | *B. thuringiensis* | AFS058004 | corn | NUFN01000025.1 | USA | 2013 | Bleich et al. (2017) |
|  | *B. thuringiensis* | AFS058004 | corn | NUFO01000082.1 | USA | 2013 | Bleich et al. (2017) |
|  | *B. thuringiensis* | AFS058113 | plant core | NUFK01000003.1 | USA | 2014 | Bleich et al. (2017) |
|  | *B. thuringiensis* | AFS058254 | Soybean | NUFJ01000018.1 | USA | 2014 | Bleich et al. (2017) |
|  | *B. thuringiensis* | AFS058532 | plant core | NVEO01000029.1 | USA | 2014 | Bleich et al. (2017) |
|  | *B. thuringiensis* | AFS058843 | plant core | NVEL01000034.1 | USA | 2014 | Bleich et al. (2017) |
|  | *B. thuringiensis* | AFS059039 | soil | NVEK01000022.1 | USA | 2014 | Bleich et al. (2017) |
|  | *B. thuringiensis* | AFS059242 | excrement | NVEH01000010.1 | USA | 2014 | Bleich et al. (2017) |
|  | *B. thuringiensis* | AFS059517 | corn | NVEF01000064.1 | USA | 2013 | Bleich et al. (2017) |
|  | *B. thuringiensis* | AFS059526 | corn | NVEE01000026.1 | USA | 2013 | Bleich et al. (2017) |
|  | *B. thuringiensis* | AFS059559 | soil | NVED01000003.1 | USA | 2014 | Bleich et al. (2017) |
|  | *B. thuringiensis* | AFS059859 | unknown | NVEA01000023.1 | USA | 2014 | Bleich et al. (2017) |
|  | *B. thuringiensis* | AFS059914 | corn | NVDY01000048.1 | USA | 2014 | Bleich et al. (2017) |
|  | *B. thuringiensis* | AFS059931 | soil | NVDX01000022.1 | USA | 2014 | Bleich et al. (2017) |
|  | *B. thuringiensis* | AFS060054 | plant core | NVDV01000049.1 | USA | 2014 | Bleich et al. (2017) |
|  | *B. thuringiensis* | AFS060060 | soybean | NVDU01000011.1 | USA | 2014 | Bleich et al. (2017) |
|  | *B. thuringiensis* | AFS061177 | soybean | NVDM01000103.1 | USA | 2014 | Bleich et al. (2017) |
|  | *B. thuringiensis* | AFS061455 | corn | NVDK01000007.1 | USA | 2014 | Bleich et al. (2017) |
|  | *B. thuringiensis* | AFS061653 | plant core | NVDI01000029.1 | USA | 2014 | Bleich et al. (2017) |
|  | *B. thuringiensis* | AFS061760 | corn | NVDH01000017.1 | USA | 2014 | Bleich et al. (2017) |
|  | *B. thuringiensis* | AFS061780 | Plant; Oats | NVJS01000046.1 | USA | 2014 | Bleich et al. (2017) |
|  | *B. thuringiensis* | AFS063640 | corn | NVCZ01000007.1 | USA | 2014 | Bleich et al. (2017) |
|  | *B. thuringiensis* | AFS063736 | soybean | NVCX01000068.1 | USA | 2014 | Bleich et al. (2017) |
|  | *B. thuringiensis* | AFS063894 | Mushroom | NVCV01000025.1 | USA | 2013 | Bleich et al. (2017) |
|  | *B. thuringiensis* | AFS064137 | soil | NVCU01000044.1 | USA | 2014 | Bleich et al. (2017) |
|  | *B. thuringiensis* | AFS064999 | soybean | NVCP01000128.1 | USA | 2014 | Bleich et al. (2017) |
|  | *B. thuringiensis* | AFS065400 | soil | NVCO01000103.1 | USA | 2014 | Bleich et al. (2017) |
|  | *B. thuringiensis* | AFS065631 | plant core | NVCL01000005.1 | USA | 2014 | Bleich et al. (2017) |
|  | *B. thuringiensis* | AFS065666 | soil | NVCK01000005.1 | USA | 2014 | Bleich et al. (2017) |
|  | *B. thuringiensis* | AFS065707 | soil | NVCJ01000026.1 | USA | 2014 | Bleich et al. (2017) |
|  | *B. thuringiensis* | AFS065753 | Earthworm | NVCI01000038.1 | USA | 2013 | Bleich et al. (2017) |
|  | *B. thuringiensis* | AFS065825 | soybean | NVCF01000009.1 | USA | 2014 | Bleich et al. (2017) |
|  | *B. thuringiensis* | AFS066175 | plant core | NVBZ01000053.1 | USA | 2014 | Bleich et al. (2017) |
|  | *B. thuringiensis* | AFS066485 | Mushroom | NVBW01000014.1 | USA | 2013 | Bleich et al. (2017) |
|  | *B. thuringiensis* | AFS066485 | Mushroom | NVBX01000045.1 | USA | 2013 | Bleich et al. (2017) |
|  | *B. thuringiensis* | AFS066671 | corn | NVBU01000010.1 | USA | 2014 | Bleich et al. (2017) |
|  | *B. thuringiensis* | AFS066710 | corn | NVBT01000053.1 | USA | 2014 | Bleich et al. (2017) |
|  | *B. thuringiensis* | AFS066836 | soil | NVBS01000011.1 | USA | 2014 | Bleich et al. (2017) |
|  | *B. thuringiensis* | AFS067605 | Mushroom | NVBL01000026.1 | USA | 2013 | Bleich et al. (2017) |
|  | *B. thuringiensis* | AFS067634 | corn | NVBJ01000030.1 | USA | 2014 | Bleich et al. (2017) |
|  | *B. thuringiensis* | AFS067808 | plant core | NVBH01000007.1 | USA | 2014 | Bleich et al. (2017) |
|  | *B. thuringiensis* | AFS070359 | corn | NVAT01000028.1 | USA | 2014 | Bleich et al. (2017) |
|  | *B. thuringiensis* | AFS071454 | soybean | NVCC01000011.1 | USA | 2014 | Bleich et al. (2017) |
|  | *B. thuringiensis* | AFS071638 | soybean | NVCA01000050.1 | USA | 2013 | Bleich et al. (2017) |
|  | *B. thuringiensis* | AFS071939 | weed | NVAE01000005.1 | USA | 2014 | Bleich et al. (2017) |
|  | *B. thuringiensis* | AFS073530 | Soybean | NUZR01000022.1 | USA | 2014 | Bleich et al. (2017) |
|  | *B. thuringiensis* | AFS075082 | Soybean | NUZF01000005.1 | USA | 2014 | Bleich et al. (2017) |
|  | *B. thuringiensis* | AFS075396 | Soybean | NUZE01000001.1 | USA | 2014 | Bleich et al. (2017) |
|  | *B. thuringiensis* | AFS075408 | soil | NVGY01000116.1 | USA | 2013 | Bleich et al. (2017) |
|  | *B. thuringiensis* | AFS075487 | Core; Other Plant | NVGW01000026.1 | USA | 2014 | Bleich et al. (2017) |
|  | *B. thuringiensis* | AFS075683 | Earthworm | NUZB01000041.1 | USA | 2013 | Bleich et al. (2017) |
|  | *B. thuringiensis* | AFS076260 | corn | NUYY01000052.1 | USA | 2014 | Bleich et al. (2017) |
|  | *B. thuringiensis* | AFS076262 | Soybean | NUYX01000004.1 | USA | 2014 | Bleich et al. (2017) |
|  | *B. thuringiensis* | AFS076597 | plant core | NUYS01000017.1 | USA | 2014 | Bleich et al. (2017) |
|  | *B. thuringiensis* | AFS076772 | plant core | NUYQ01000011.1 | USA | 2014 | Bleich et al. (2017) |
|  | *B. thuringiensis* | AFS076861 | tree leaf | NUYP01000038.1 | USA | 2014 | Bleich et al. (2017) |
|  | *B. thuringiensis* | AFS077661 | corn | NUYG01000013.1 | USA | 2014 | Bleich et al. (2017) |
|  | *B. thuringiensis* | AFS078332 | Weed | NUYC01000310.1 | USA | 2014 | Bleich et al. (2017) |
|  | *B. thuringiensis* | AFS079576 | plant core | NUXU01000022.1 | USA | 2014 | Bleich et al. (2017) |
|  | *B. thuringiensis* | AFS080155 | corn | NVGD01000001.1 | USA | 2014 | Bleich et al. (2017) |
|  | *B. thuringiensis* | AFS081470 | Weed | NUXE01000007.1 | USA | 2014 | Bleich et al. (2017) |
|  | *B. thuringiensis* | AFS081620 | Soybean | NUXA01000042.1 | USA | 2014 | Bleich et al. (2017) |
|  | *B. thuringiensis* | AFS082247 | corn | NUWX01000045.1 | USA | 2014 | Bleich et al. (2017) |
|  | *B. thuringiensis* | AFS082639 | plant core | NUWS01000035.1 | USA | 2014 | Bleich et al. (2017) |
|  | *B. thuringiensis* | AFS083288 | plant core | NUWL01000001.1 | USA | 2014 | Bleich et al. (2017) |
|  | *B. thuringiensis* | AFS084862 | Corn | NUWB01000018.1 | USA | 2014 | Bleich et al. (2017) |
|  | *B. thuringiensis* | AFS085011 | plant core | NUWA01000048.1 | USA | 2014 | Bleich et al. (2017) |
|  | *B. thuringiensis* | AFS085496 | plant core | NUVX01000019.1 | USA | 2014 | Bleich et al. (2017) |
|  | *B. thuringiensis* | AFS085829 | plant core | NUVS01000058.1 | USA | 2014 | Bleich et al. (2017) |
|  | *B. thuringiensis* | AFS086178 | unknown | NUVP01000049.1 | USA | 2014 | Bleich et al. (2017) |
|  | *B. thuringiensis* | AFS086961 | soil | NUVF01000030.1 | USA | 2014 | Bleich et al. (2017) |
|  | *B. thuringiensis* | AFS087171 | plant core | NUVE01000009.1 | USA | 2014 | Bleich et al. (2017) |
|  | *B. thuringiensis* | AFS088016 | soil | NUUZ01000010.1 | USA | 2014 | Bleich et al. (2017) |
|  | *B. thuringiensis* | AFS088507 | soil | NUUT01000019.1 | USA | 2014 | Bleich et al. (2017) |
|  | *B. thuringiensis* | AFS089089 | corn | NVNL01000009.1 | USA | 2013 | Bleich et al. (2017) |
|  | *B. thuringiensis* | AFS089732 | corn | NVNT01000006.1 | USA | 2014 | Bleich et al. (2017) |
|  | *B. thuringiensis* | AFS090355 | corn | NVNH01000003.1 | USA | 2014 | Bleich et al. (2017) |
|  | *B. thuringiensis* | AFS090477 | soybean | NVNF01000068.1 | USA | 2014 | Bleich et al. (2017) |
|  | *B. thuringiensis* | AFS092108 | soybean | NVNA01000013.1 | USA | 2014 | Bleich et al. (2017) |
|  | *B. thuringiensis* | AFS093126 | soil | NVMU01000044.1 | USA | 2014 | Bleich et al. (2017) |
|  | *B. thuringiensis* | AFS093334 | soybean | NVNS01000004.1 | USA | 2014 | Bleich et al. (2017) |
|  | *B. thuringiensis* | AFS094329 | plant core | NVMK01000004.1 | USA | 2014 | Bleich et al. (2017) |
|  | *B. thuringiensis* | AFS094706 | unknown | NVMG01000022.1 | USA | 2014 | Bleich et al. (2017) |
|  | *B. thuringiensis* | AFS094730 | Soybean | NVMF01000006.1 | USA | 2014 | Bleich et al. (2017) |
|  | *B. thuringiensis* | AFS094922 | plant core | NVME01000059.1 | USA | 2014 | Bleich et al. (2017) |
|  | *B. thuringiensis* | AFS094940 | Soybean | NVMD01000006.1 | USA | 2014 | Bleich et al. (2017) |
|  | *B. thuringiensis* | AFS095482 | excrement | NVMA01000015.1 | USA | 2014 | Bleich et al. (2017) |
|  | *B. thuringiensis* | AFS095788 | Soybean | NVLU01000073.1 | USA | 2014 | Bleich et al. (2017) |
|  | *B. thuringiensis* | AFS095989 | Soybean | NVLS01000068.1 | USA | 2014 | Bleich et al. (2017) |
|  | *B. thuringiensis* | AFS096410 | corn | NVLO01000042.1 | USA | 2014 | Bleich et al. (2017) |
|  | *B. thuringiensis* | AFS096463 | Mushroom | NVLM01000002.1 | USA | 2013 | Bleich et al. (2017) |
|  | *B. thuringiensis* | AFS096719 | corn | NVNP01000011.1 | USA | 2014 | Bleich et al. (2017) |
|  | *B. thuringiensis* | AFS096960 | corn | NVLI01000002.1 | USA | 2014 | Bleich et al. (2017) |
|  | *B. thuringiensis* | AFS096978 | corn | NVLH01000018.1 | USA | 2014 | Bleich et al. (2017) |
|  | *B. thuringiensis* | AFS097325 | plant core | NVLE01000020.1 | USA | 2014 | Bleich et al. (2017) |
|  | *B. thuringiensis* | AFS097488 | corn | NVKZ01000008.1 | USA | 2014 | Bleich et al. (2017) |
|  | *B. thuringiensis* | AFS097514 | Soybean | NVKY01000168.1 | USA | 2014 | Bleich et al. (2017) |
|  | *B. thuringiensis* | AFS097856 | Soybean | NVKX01000032.1 | USA | 2014 | Bleich et al. (2017) |
|  | *B. thuringiensis* | AFS097917 | plant core | NVKW01000012.1 | USA | 2014 | Bleich et al. (2017) |
|  | *B. thuringiensis* | AFS098336 | Soybean | NVKS01000043.1 | USA | 2014 | Bleich et al. (2017) |
|  | *B. thuringiensis* | AFS098399 | Soybean | NVKR01000053.1 | USA | 2014 | Bleich et al. (2017) |
|  | *B. thuringiensis* | AFS098506 | Corn | NVKQ01000030.1 | USA | 2014 | Bleich et al. (2017) |
|  | *B. thuringiensis* | AFS099341 | Corn | NVKK01000018.1 | USA | 2014 | Bleich et al. (2017) |
|  | *B. thuringiensis* | AFS099505 | Soybean | NVKJ01000014.1 | USA | 2014 | Bleich et al. (2017) |
|  | *B. thuringiensis* | AFS099506 | Corn | NVKI01000028.1 | USA | 2014 | Bleich et al. (2017) |
|  | *B. thuringiensis* | AFS099677 | plant core | NVKG01000035.1 | USA | 2014 | Bleich et al. (2017) |
|  | *B. thuringiensis* | AGES_2_27_S | Homo sapiens | JAIVBJ010000050.1 | unknown | 2013 | Biggel (2021) |
|  | *B. thuringiensis* | AGES_6_27_S | Homo sapiens | JAIVBI010000048.1 | unknown | 2013 | Biggel (2021) |
|  | *B. thuringiensis* | AK47 | soil | JWST01000033.1 | India | 2010 | Saxena et al. (2015) |
|  | *B. thuringiensis* | Al Hakam | unknown | CP000485.1 | unknown | unknown | Challacombe et al. (2007) |
|  | *B. thuringiensis* | AM65-52 | unknown | CP013275 | France | 2014 | Bolotin et al. (2016) |
|  | *B. thuringiensis* | AmN0-4 | sediment | JAIUXV000000000.1 | China | 2021 | Yu et al. (2021) |
|  | *B. thuringiensis* | AR23 | unknown | CAAKHA010000027.1 | unknown | unknown | Patino-Navarrete and Patino Navarrete (2019) |
|  | *B. thuringiensis* | AS23 | soil | JAVIAF010000001.1 | South Korea | 2022 | Shin et al. (2023) |
|  | *B. thuringiensis* | ATCC 10792 | Tissue, animal | CP020754.1 | USA | 1946 | Kim et al. (2017) |
|  | *B. thuringiensis* | ATCC 13367 | unknown | VKQK01000046.1 | unknown | unknown | Riojas et al. (2019) |
|  | *B. thuringiensis* | ATCC 19266 | unknown | VKQL01000001.1 | unknown | unknown | Riojas et al. (2019) |
|  | *B. thuringiensis* | ATCC 33679 | unknown | VKQM01000125.1 | unknown | unknown | Riojas et al. (2019) |
|  | ***B. thuringiensis*** | **ATCC 35646** | **unknown** | **AAJM01000191.1** | **unknown** | **unknown** | **Anderson et al. (2005)** |
|  | *B. thuringiensis* | ATCC 700872 | unknown | VKQO01000037.1 | unknown | unknown | Riojas et al. (2019) |
|  | *B. thuringiensis* | B116 | soil | JABXFG010000015.1 | Brazil | 2004 | Morais De Sousa et al. (2020) |
|  | *B. thuringiensis* | B13 | moth | CP074714 | USA | 1952 | Shwed et al. (2022) |
|  | *B. thuringiensis* | B-2012 | Sediments | LZRA01000027.1 | Pacific Ocean | 2002 | Liu et al. (2016) |
|  | *B. thuringiensis* | B401 | Biopesticide B401/B402 (Certan) | CP083085 | unknown | unknown | Biggel (2021) |
|  | *B. thuringiensis* | BA04 | soil | PUWY01000094.1 | india | 2013 | Rabha et al. (2018) |
|  | *B. thuringiensis* | Bacillus sp. B140 | food | DAOIBX010000012.1 | Thailand | 2018 | Souvorov et al. (2023) |
|  | *B. thuringiensis* | BAM | soil | QUQW01000006.1 | USA | 2018 | Maine and Sevigny (2018) |
|  | *B. thuringiensis* | BC601 | unknown | CP015150 | China | 2012 | Jia et al.  (2016) |
|  | *B. thuringiensis* | BGSC 4A3 | Bombyx mori | PGDU01000044.1 | United Kingdom | 2013 | Pascoe et al. (2017) |
|  | *B. thuringiensis* | BGSC 4A4 | Ephestia elutella | NFCE01000013.1 | United Kingdom | unknown | Zheng et al. (2016) |
|  | *B. thuringiensis* | BGSC 4AA1 | soil | CP010577.1 | unknown | 1987 | Gao et al. (2015) |
|  | *B. thuringiensis* | BGSC 4AC1 | soil | NFCF01000098.1 | Mexico | 1988 | Zheng et al. (2016) |
|  | *B. thuringiensis* | BGSC 4AD1 | soil | NFCG01000092.1 | USSR | 1990 | Zheng et al. (2016) |
|  | *B. thuringiensis* | BGSC 4AE1 | Bombyx mori litter | NFCH01000117.1 | Japan | 1996 | Zheng et al. (2016) |
|  | *B. thuringiensis* | BGSC 4AG1 | grain silo | NFCJ01000105.1 | France | unknown | Zheng et al. (2016) |
|  | *B. thuringiensis* | BGSC 4AH1 | unknown | MOOF01000200.1 | South Korea | 1994 | Zheng et al. (2016) |
|  | *B. thuringiensis* | BGSC 4AJ1 | unknown | CM000752.1 | unknown | unknown | Read et al. (2009) |
|  | *B. thuringiensis* | BGSC 4AK1 | unknown | MOOG01000009.1 | South Korea | 1994 | Zheng et al. (2016) |
|  | *B. thuringiensis* | BGSC 4AL1 | unknown | NFCL01000038.1 | South Korea | 1994 | Zheng et al. (2016) |
|  | *B. thuringiensis* | BGSC 4AM1 | soil | MOOH01000168.1 | China | 1979 | Zheng et al. (2016) |
|  | *B. thuringiensis* | BGSC 4AO1 | unknown | NFCM01000089.1 | Japan | 1989 | Zheng et al. (2016) |
|  | *B. thuringiensis* | BGSC 4AP1 | unknown | NFCN01000100.1 | Japan | 1989 | Zheng et al. (2016) |
|  | *B. thuringiensis* | BGSC 4AR1 | soil | NFCP01000064.1 | China | unknown | Zheng et al. (2016) |
|  | *B. thuringiensis* | BGSC 4AS1 | black pepper power | MOOJ01000047.1 | Brazil | unknown | Zheng et al. (2016) |
|  | *B. thuringiensis* | BGSC 4AT1 | unknown | NFCQ01000065.1 | Japan | 1986 | Zheng et al. (2016) |
|  | *B. thuringiensis* | BGSC 4AU1 | Bombyx mori litter | MOOK01000077.1 | Japan | unknown | Zheng et al. (2016) |
|  | *B. thuringiensis* | BGSC 4AW1 | unknown | CM000754.1 | unknown | unknown | Read et al. (2009)‎ |
|  | *B. thuringiensis* | BGSC 4AX1 | soil | NFCT01000032.1 | USSR | unknown | Zheng et al. (2016) |
|  | *B. thuringiensis* | BGSC 4AY1 | black pepper power | NFCU01000059.1 | Brazil | unknown | Zheng et al. (2016) |
|  | *B. thuringiensis* | BGSC 4AZ1 | unknown | NFCV01000033.1 | Czechoslovakia | 1984 | Zheng et al. (2016) |
|  | *B. thuringiensis* | BGSC 4BA1 | unknown | CM000755.1 | unknown | unknown | Read et al. (2009)‎ |
|  | *B. thuringiensis* | BGSC 4BB1 | unknown | NFCY01000024.1 | South Korea | unknown | Zheng et al. (2016) |
|  | *B. thuringiensis* | BGSC 4BC1 | soil | NFCZ01000040.1 | China | unknown | Zheng et al. (2016) |
|  | *B. thuringiensis* | BGSC 4BD1 | unknown | CM000756.1 | unknown | unknown | Read et al. (2009)‎ |
|  | *B. thuringiensis* | BGSC 4BF1 | unknown | NFDA01000077.1 | Germany | unknown | Zheng et al. (2016) |
|  | *B. thuringiensis* | BGSC 4BG1 | ivy leaves | NFDB01000031.1 | Denmark | unknown | Zheng et al. (2016) |
|  | *B. thuringiensis* | BGSC 4BH1 | rice paddy | NFDC01000087.1 | Thailand | unknown | Zheng et al. (2016) |
|  | *B. thuringiensis* | BGSC 4BJ1 | soil | NFDD01000020.1 | Poland | unknown | Zheng et al. (2016) |
|  | *B. thuringiensis* | BGSC 4BL1 | unknown | NFDF01000043.1 | Argentina | unknown | Zheng et al. (2016) |
|  | *B. thuringiensis* | BGSC 4BM1 | soil | NFDG01000124.1 | Spain | unknown | Zheng et al. (2016) |
|  | *B. thuringiensis* | BGSC 4BN1 | bean | MOOL01000016.1 | China | unknown | Zheng et al. (2016) |
|  | *B. thuringiensis* | BGSC 4BP1 | sesame field | NFDH01000060.1 | South Korea | unknown | Zheng et al. (2016) |
|  | *B. thuringiensis* | BGSC 4BR1 | Lymantria monacha | NFDJ01000032.1 | Denmark | unknown | Zheng et al. (2016) |
|  | *B. thuringiensis* | BGSC 4BS1 | soil | MOOM01000014.1 | Spain | unknown | Zheng et al. (2016) |
|  | *B. thuringiensis* | BGSC 4BT1 | red soil | LILG01000037.1 | China | 1999 | Xin et al. (2015) |
|  | *B. thuringiensis* | BGSC 4BU1 | unknown | MOON01000021.1 | Spain | unknown | Zheng et al. (2016) |
|  | *B. thuringiensis* | BGSC 4BV1 | unknown | MOOO01000058.1 | Argentina | unknown | Zheng et al. (2016) |
|  | *B. thuringiensis* | BGSC 4BW1 | unknown | MOOP01000064.1 | Spain | unknown | Zheng et al. (2016) |
|  | *B. thuringiensis* | BGSC 4BX1 | Sandy soil | NFDL01000080.1 | China | unknown | Zheng et al. (2016) |
|  | *B. thuringiensis* | BGSC 4BY1 | Scotch pine | MOOQ01000004.1 | Denmark | unknown | Zheng et al. (2016) |
|  | *B. thuringiensis* | BGSC 4BZ1 | black soil | MOOR01000120.1 | China | unknown | Zheng et al. (2016) |
|  | *B. thuringiensis* | BGSC 4C1 | Bombyx mori | CP015176 | Czechoslovakia | 1987 | Wang and Sun (2016) |
|  | *B. thuringiensis* | BGSC 4C3 | Bombyx mori | PGSC01000601.1 | United Kingdom | 2013 | Pascoe et al. (2017) |
|  | *B. thuringiensis* | BGSC 4CA1 | unknown | NFDN01000070.1 | South Korea | unknown | Zheng et al. (2016) |
|  | *B. thuringiensis* | BGSC 4CB1 | soil | NFDO01000163.1 | Portugal | unknown | Zheng et al. (2016) |
|  | *B. thuringiensis* | BGSC 4CC1 | unknown | CM000757.1 | unknown | unknown | Read et al. (2009)‎ |
|  | *B. thuringiensis* | BGSC 4CD1 | soil | NFDP01000041.1 | Portugal | unknown | Zheng et al. (2016) |
|  | *B. thuringiensis* | BGSC 4CE1 | soil | NFDQ01000077.1 | Portugal | unknown | Zheng et al. (2016) |
|  | *B. thuringiensis* | BGSC 4CF1 | unknown | MOOS01000159.1 | Malaysia | unknown | Zheng et al. (2016) |
|  | *B. thuringiensis* | BGSC 4D1 | Bombyx mori | PGEG01000314.1 | United Kingdom | 2013 | Pascoe et al. (2017) |
|  | *B. thuringiensis* | BGSC 4D4 | Bombyx mori | PHSM01000001.1 | United Kingdom | 2013 | Pascoe et al. (2017) |
|  | *B. thuringiensis* | BGSC 4E3 | Bombyx mori | PGDV01000437.1 | United Kingdom | 2013 | Pascoe et al. (2017) |
|  | *B. thuringiensis* | BGSC 4G1 | Galleria mellonella | NFDR01000184.1 | USSR | 1958 | Zheng et al. (2016) |
|  | *B. thuringiensis* | BGSC 4G4 | Bombyx mori | PGDZ01000026.1 | United Kingdom | 2013 | Pascoe et al. (2017) |
|  | *B. thuringiensis* | BGSC 4H2 | unknown | NFDS01000013.1 | Canada | 1972 | Zheng et al. (2016) |
|  | *B. thuringiensis* | BGSC 4I4 | Paralipsa gularis | NFDT01000201.1 | Canada | 1958 | Zheng et al. (2016) |
|  | *B. thuringiensis* | BGSC 4J1 | Heliothis assulta | NFDU01000151.1 | Japan | unknown | Zheng et al. (2016) |
|  | *B. thuringiensis* | BGSC 4J2 | Plodia interpunctella | NFDV01000189.1 | United Kingdom | unknown | Zheng et al. (2016) |
|  | *B. thuringiensis* | BGSC 4J3 | Plodia interpunctella | NFDW01000225.1 | United Kingdom | unknown | Zheng et al. (2016) |
|  | *B. thuringiensis* | BGSC 4J4 | Heliothis assulta | NFDX01000069.1 | Japan | unknown | Zheng et al. (2016) |
|  | *B. thuringiensis* | BGSC 4J5 | Plodia interpunctella | NFDY01000129.1 | United Kingdom | unknown | Zheng et al. (2016) |
|  | *B. thuringiensis* | BGSC 4K1 | Bombyx mori | PGEC01000171.1 | United Kingdom | 2013 | Pascoe et al. (2017) |
|  | *B. thuringiensis* | BGSC 4L3 | unknown | NFDZ01000154.1 | unknown | unknown | Zheng et al. (2016) |
|  | *B. thuringiensis* | BGSC 4M3 | unknown | NFEA01000049.1 | United Kingdom | 1968 | Zheng et al. (2016) |
|  | *B. thuringiensis* | BGSC 4N1 | Galleria mellonella | NFEB01000116.1 | United Kingdom | 1956 | Zheng et al. (2016) |
|  | *B. thuringiensis* | BGSC 4O1 | unknown | NFEC01000145.1 | unknown | unknown | Zheng et al. (2016) |
|  | *B. thuringiensis* | BGSC 4Q1 | Culicidae | NFED01000025.1 | Israel | 1977 | Zheng et al. (2016) |
|  | *B. thuringiensis* | BGSC 4Q7rifR | unknown | CP051858 | Belgium | 2016 | Bolotin et al. (2020) |
|  | *B. thuringiensis* | BGSC 4R1 | unknown | MOOT01000025.1 | unknown | unknown | Zheng et al. (2016) |
|  | *B. thuringiensis* | BGSC 4T1 | soil | NFEE01000124.1 | China | 1976 | Zheng et al. (2016) |
|  | *B. thuringiensis* | BGSC 4U1 | Bombyx mori | NFEF01000062.1 | Japan | 1979 | Zheng et al. (2016) |
|  | *B. thuringiensis* | BGSC 4V1 | Bombyx mori | NFEG01000171.1 | Japan | 1981 | Zheng et al. (2016) |
|  | *B. thuringiensis* | BGSC 4W1 | Bombyx mori | NFEH01000036.1 | Japan | 1981 | Zheng et al. (2016) |
|  | *B. thuringiensis* | BGSC 4X1 | unknown | NFEI01000160.1 | unknown | 1984 | Zheng et al. (2016) |
|  | *B. thuringiensis* | BGSC 4Y1 | unknown | CM000746.1 | unknown | unknown | Read et al. (2009) |
|  | *B. thuringiensis* | BGSC 4Z1 | unknown | NFEJ01000024.1 | China | 1975 | Zheng et al. (2016) |
|  | *B. thuringiensis* | BHI1-bin.2 | Apis mellifera | JAMBIV010000003.1 | China | 2020 | Meng (2022) |
|  | *B. thuringiensis* | BI 256-82 | Tenebrio molitor | CP133362.1 | Germany | 1983 | Schaefer et al. (2023) |
|  | *B. thuringiensis* | BLB1 | unknown | CP116325.1 | Tunisia | 2008 | Fayad et al. (2023) |
|  | *B. thuringiensis* | BLB406 | soil | MRZW01000142.1 | Tunisia | 2009 | Zribi Zghal (2018) |
|  | *B. thuringiensis* | BM311.1 | soil | SSWY01000056.1 | Spain | 1998 | Dominguez et al. (2019) |
|  | *B. thuringiensis* | BMB171 | unknown | CP001903.1 | unknown | unknown | Shao et al. 2010 |
|  | *B. thuringiensis* | BMB3201 | soil | LDEU01000101.1 | China | 2015 | Xin et al. (2015) |
|  | *B. thuringiensis* | BM-BT15426 | unknown | CP020723 | China | 2015 | Liu et al. (2017) |
|  | *B. thuringiensis* | BMP144 | Biopesticide Solbac | CP083134 | unknown | 2017 | Biggel (2021) |
|  | *B. thuringiensis* | BP865 | phylloplane | MOXR01000041.1 | South Korea | 1993 | Jeong et al. (2016) |
|  | *B. thuringiensis* | BR145 | soil | PDVK01000041.1 | Brazil | 2010 | Ricietto et al. (2017) |
|  | *B. thuringiensis* | BR37 | stored grains | LOMJ01000027.1 | Brazil | 2009 | Constanski et al. (2015) |
|  | *B. thuringiensis* | BR58 | stored grain | LIIT01000065.1 | Brazil | 2000 | Zorzetti et al. (2015) |
|  | *B. thuringiensis* | Bt Gxmzu777-1 | soil | CP097257 | China | 2022 | Yang (2022) |
|  | *B. thuringiensis* | Bt18247 | Caenorhabditis elegans | CP015250 | unknown | unknown | Hollensteiner et al. (2016) |
|  | *B. thuringiensis* | BT185 | soil | CP014282 | China | 2002 | Shu (2016) |
|  | *B. thuringiensis* | Bt407 | unknown | CM000747.1 | unknown | unknown | Read et al 2009 |
|  | *B. thuringiensis* | Serovar berliner strain Bt407 | Bombyx mori | PHSK01000001.1 | United Kingdom | 2013 | Pascoe et al. (2017) |
|  | *B. thuringiensis* | BT-59 | soil | CP039721 | China | 2015 | Song and Xu et al. (2019) |
|  | *B. thuringiensis* | BT62 | soi | CP044978 | India | 2010 | Murugan et al. (2018) |
|  | *B. thuringiensis* | BTG | Rhisosphere | JASNQP010000039.1 | Bulgaria | 2002 | Petrova et al. (2023) |
|  | *B. thuringiensis* | Bt-GS57 | soil | CP043234 | China | 2011 | Zhao et al. (2019) |
|  | *B. thuringiensis* | Bti | soil | CP037890 | Brazil | 2015 | Alves et al. (2019) |
|  | *B. thuringiensis* | Bti-185 | insect | SCMJ01000027.1 | China | 2005 | Liu and Shu (2005) |
|  | *B. thuringiensis* | Bt-ICA/UNMSM10RA | Agr | PZQR01000030.1 | Peru | 2010 | Serna et al. (2018) |
|  | *B. thuringiensis* | Bto-UNVM_94 | soil | QGLX01000001.1 | Argentina | 2015 | Palma et al. (2018) |
|  | *B. thuringiensis* | Buibui | Coleoptera | PGDR01000049.1 | United Kingdom | 2013 | Pascoe et al. (2017) |
|  | *B. thuringiensis* | C15 | soil | CP021436 | China | 1970 | Wang and Sun (2017) |
|  | *B. thuringiensis* | C1R06_2_rep | ENVO:00002150 | JAUORT010000045.1 | USA | 2015 | Gralka (2023) |
|  | *B. thuringiensis* | c-21 | ob/ob mouse | RAYC01000001.1 | China | 2016 | Liu (2018) |
|  | *B. thuringiensis* | C25 | shrimp | CP022245 | South Korea | 2017 | Ryu (2017) |
|  | *B. thuringiensis* | C3R06 | ENVO:00002150 | JAUORF010000028.1 | USA | 2015 | Gralka (2023) |
|  | *B. thuringiensis* | C3R06_2 | ENVO:00002150 | JAUORE010000045.1 | USA | 2015 | Gralka (2023) |
|  | *B. thuringiensis* | CF4_25 | Cultured Food | JAHFZT010000010.1 | USA | 2018 | Barnaba et al. (2021) |
|  | *B. thuringiensis* | CH_48 | rosemary | JABERC010000005.1 | Switzerland | 2017 | Stevens et al. (2020) |
|  | *B. thuringiensis* | CH_65 | food | JAIVBL010000047.1 | unknown | 2016 | Biggel (2021) |
|  | *B. thuringiensis* | CH_684 | food | JAIVCA010000042.1 | unknown | 2020 | Biggel (2021) |
|  | *B. thuringiensis* | CH_69 | food | JAIVBQ010000043.1 | unknown | 2016 | Biggel (2021) |
|  | *B. thuringiensis* | CH_692 | food | JAIVBZ010000047.1 | unknown | 2020 | Biggel (2021) |
|  | *B. thuringiensis* | CH_696 | food | JAIVBY010000047.1 | unknown | 2020 | Biggel (2021) |
|  | *B. thuringiensis* | CH_698 | food | JAIVCB010000042.1 | unknown | 2020 | Biggel (2021) |
|  | *B. thuringiensis* | CH_709 | food | JAIVBO010000046.1 | unknown | 2020 | Biggel (2021) |
|  | *B. thuringiensis* | CH_72 | food | JAIVBK010000049.1 | unknown | 2016 | Biggel (2021) |
|  | *B. thuringiensis* | CH_722 | food | JAIVBW010000038.1 | unknown | 2020 | Biggel (2021) |
|  | *B. thuringiensis* | CH_733 | food | JAIVBX010000047.1 | unknown | 2020 | Biggel (2021) |
|  | *B. thuringiensis* | CH_746 | food | JAIVBN010000048.1 | unknown | 2020 | Biggel (2021) |
|  | *B. thuringiensis* | CH_748 | food | JAIVBM010000047.1 | unknown | 2020 | Biggel (2021) |
|  | *B. thuringiensis* | CH_81 | food | JAIVBP010000041.1 | unknown | 2016 | Biggel (2021) |
|  | *B. thuringiensis* | CIP53137 | unknown | DAOIWY010000007.1 | France | 2015 | Souvorov et al. (2018) |
|  | *B. thuringiensis* | CT-43 | unknown | CP001907.1 | unknown | unknown | Shao et al. (2010) |
|  | *B. thuringiensis* | CTC | invertebrates | CP013274 | China | 1999 | Dong et al. (2015) |
|  | *B. thuringiensis* | CVUAS2492 | food | JAIVBT010000049.1 | unknown | 2012 | Biggel (2021) |
|  | *B. thuringiensis* | CVUAS9659 | food | JAIVBS010000047.1 | unknown | 2012 | Biggel (2021) |
|  | *B. thuringiensis* | CVUAS9660 | food | JAIVBR010000049.1 | unknown | 2012 | Biggel (2021) |
|  | *B. thuringiensis* | D3A2_S7 | water | JAMBML010000015.1 | Israel | 2019 | Chemla et al. (2022) |
|  | *B. thuringiensis* | DAR 81934 | unknown | CM001804.1 | Australia | unknown | Wang et al. (2012) |
|  | *B. thuringiensis* | DB27 | unknown | HG810020.1 | unknown | unknown | Aslett (2014) |
|  | *B. thuringiensis* | DBG15 | soil | VLPM01000039.1 | Viet Nam | 2015 | Le et al. (2019) |
|  | *B. thuringiensis* | DE0030 | environmental | VEIF01000030.1 | USA | 2018 | Song (2019) |
|  | *B. thuringiensis* | DE0031 | environmental | VEIE01000004.1 | USA | 2018 | Song (2019) |
|  | *B. thuringiensis* | DE0141 | environmental | VEFL01000024.1 | USA | 2018 | Song (2019) |
|  | *B. thuringiensis* | DE0161 | environmental | VEEU01000039.1 | USA | 2018 | Song (2019) |
|  | *B. thuringiensis* | DE0163 | environmental | VEES01000004.1 | USA | 2018 | Song (2019) |
|  | *B. thuringiensis* | DE0177 | environmental | VEEG01000003.1 | USA | 2018 | Song (2019) |
|  | *B. thuringiensis* | DE0280 | environmental | VEBR01000004.1 | USA | 2018 | Song (2019) |
|  | *B. thuringiensis* | DE0298 | environmental | VEBH01000003.1 | USA | 2018 | Song (2019) |
|  | *B. thuringiensis* | DE0302 | environmental | VEBE01000038.1 | USA | 2018 | Song (2019) |
|  | *B. thuringiensis* | DE0320 | environmental | VEAP01000004.1 | USA | 2018 | Song (2019) |
|  | *B. thuringiensis* | DE0326 | environmental | VEAN01000004.1 | USA | 2018 | Song (2019) |
|  | *B. thuringiensis* | DE0332 | environmental | VEAI01000026.1 | USA | 2018 | Song (2019) |
|  | *B. thuringiensis* | DE0343 | environmental | VEAC01000011.1 | USA | 2018 | Song (2019) |
|  | *B. thuringiensis* | DE0365 | environmental | VDZK01000023.1 | USA | 2018 | Song (2019) |
|  | *B. thuringiensis* | DE0472 | environmental | VDRD01000011.1 | USA | 2018 | Song (2019) |
|  | *B. thuringiensis* | DE0502 | environmental | VDQF01000005.1 | USA | 2018 | Song (2019) |
|  | *B. thuringiensis* | DE0537 | environmental | VDPB01000002.1 | USA | 2018 | Song (2019) |
|  | *B. thuringiensis* | DE0555 | environmental | VDTR01000007.1 | USA | 2018 | Song (2019) |
|  | *B. thuringiensis* | DE0589 | environmental | VDNN01000040.1 | USA | 2018 | Song (2019) |
|  | *B. thuringiensis* | DE0604 | environmental | VDNB01000004.1 | USA | 2018 | Song (2019) |
|  | *B. thuringiensis* | DE0606 | environmental | VDNA01000029.1 | USA | 2018 | Song (2019) |
|  | *B. thuringiensis* | delfin | leaves | JAOAOX010000043.1 | Belgium | 2020 | Zervas (2022) |
|  | *B. thuringiensis* | DHC4 | termite gut | CP126474.1 | China | 2021 | Cheng and Pan (2023) |
|  | *B. thuringiensis* | dipel | leaves | JAOAOW010000041.1 | Belgium | 2020 | Zervas (2022) |
|  | *B. thuringiensis* | DNG9 | oil-contaminated slough | MSTN01000006.1 | Algeria | 2013 | Rosana et al. (2017) |
|  | *B. thuringiensis* | DPC6431 | Homo sapiens | SCLP01000005.1 | Ireland | 2008 | Arbulu et al. (2019) |
|  | *B. thuringiensis* | Et10/1 | water | JYCI01000001.1 | Chile | 2011 | Filippidou et al. (2014) |
|  | ***B. thuringiensis*** | **F14-1** | **soil** | **JZKB01000002.1** | **China** | **2002** | **Geng et al. (2015)** |
|  | *B. thuringiensis* | FC1 | food | JARAGA010000043.1 | United Kingdom | 2007 | Pheepakpraw (2023) |
|  | *B. thuringiensis* | FC2 | food | JARAFZ010000010.1 | United Kingdom | 2007 | Pheepakpraw (2023) |
|  | *B. thuringiensis* | FC7 | vomit | JARAFY010000038.1 | United Kingdom | 2007 | Pheepakpraw (2023) |
|  | *B. thuringiensis* | FC8 | vomit | JARAFX010000038.1 | United Kingdom | 2007 | Pheepakpraw (2023) |
|  | *B. thuringiensis* | FCC7 | soil | VIXF01000002.1 | Argentina | 2005 | Lazarte et al. (2019) |
|  | *B. thuringiensis* | FDA1189054-S034-001 | pumpkin baby food | DAOIBV010000005.1 | USA | 2023 | Souvorov et al. (2023) |
|  | *B. thuringiensis* | FDA1189054-S034-002 | pumpkin baby food | DAOIBU010000004.1 | USA | 2023 | Souvorov et al. (2023) |
|  | *B. thuringiensis* | FDA1196921-S012-002 | tattoo ink cocconut | DAQDDI010000003.1 | USA | 2023 | Souvorov et al. (2018) |
|  | *B. thuringiensis* | FDAARGOS_791 | unknown | CP054568 | USA | unknown | Nelson et al. (2020) |
|  | *B. thuringiensis* | FDAARGOS_792 | unknown | CP053938 | USA | unknown | Nelson et al. (2020) |
|  | *B. thuringiensis* | FDAARGOS_793 | unknown | CP053981 | USA | unknown | Nelson et al. (2020) |
|  | *B. thuringiensis* | FDAARGOS_796 | unknown | CP053972 | USA | unknown | Nelson et al. (2020) |
|  | *B. thuringiensis* | FDAARGOS_800 | unknown | JABWHT010000004.1 | USA | unknown | Nelson et al. (2020) |
|  | *B. thuringiensis* | FDAARGOS_801 | unknown | JABVAQ010000006.1 | USA | unknown | Nelson et al. (2020) |
|  | *B. thuringiensis* | FDAARGOS-794 | unknown | CP053934 | USA | unknown | Nelson et al. (2020) |
|  | *B. thuringiensis* | FDAARGOS-795 | unknown | CP053980 | USA | unknown | Nelson et al. (2020) |
|  | *B. thuringiensis* | FN31 | Sediments | LZRC01000086.1 | Pacific Ocean | 2002 | Liu et al. (2016) |
|  | *B. thuringiensis* | G03 | soil | NHNQ01000039.1 | China | 2003 | Shu (2017) |
|  | *B. thuringiensis* | G25-120 | unknown | LDIA01000047.1 | USA | 2011 | Zheng and Sun (2015) |
|  | *B. thuringiensis* | G25-121 | unknown | LDIB01000052.1 | unknown | 2011 | Zheng and Sun (2015) |
|  | *B. thuringiensis* | G25-123 | dead mosquito larvae | LDID01000037.1 | Burma | 2011 | Zheng and Sun (2015) |
|  | *B. thuringiensis* | G25-13 | unknown | LPZY01000395.1 | China | 2011 | Zheng and Sun (2015) |
|  | *B. thuringiensis* | G25-18 | soil | LPZZ01000234.1 | China | 2011 | Zheng and Sun (2015) |
|  | *B. thuringiensis* | G25-39 | maybe soil | LDFI01000020.1 | China | 2011 | Zheng and Sun (2015) |
|  | *B. thuringiensis* | G25-41 | maybe soil | LDFJ01000168.1 | USA | 2011 | Zheng and Sun (2015) |
|  | *B. thuringiensis* | G25-42 | soil | LDER01000197.1 | USA | 2012 | Zheng and Sun (2018) |
|  | *B. thuringiensis* | G25-43 | maybe soil | LDFL01000464.1 | USA | 2011 | Zheng and Sun (2015) |
|  | *B. thuringiensis* | G25-44 | soil | LDEQ01000666.1 | USA | 2012 | Zheng and Sun (2015) |
|  | *B. thuringiensis* | G25-45 | maybe soil | LDFM01000136.1 | USA | 2011 | Zheng and Sun (2015) |
|  | *B. thuringiensis* | G25-47 | maybe soil | LDFO01000030.1 | USA | 2011 | Zheng and Sun (2015) |
|  | *B. thuringiensis* | G25-48 | maybe soil | LDFP01000051.1 | USA | 2011 | Zheng and Sun (2015) |
|  | *B. thuringiensis* | G25-49 | maybe soil | LDFQ01000177.1 | USA | 2011 | Zheng and Sun (2015) |
|  | *B. thuringiensis* | G25-5 | soil | LDJQ01000041.1 | USA | 2011 | Zheng and Sun (2015) |
|  | *B. thuringiensis* | G25-50 | maybe soil | LDFR01000105.1 | USA | 2011 | Zheng and Sun (2015) |
|  | *B. thuringiensis* | G25-51 | maybe soil | LDFS01000556.1 | USA | 2011 | Zheng and Sun (2015) |
|  | *B. thuringiensis* | G25-52 | maybe soil | LDFT01000357 | USA | 2011 | Zheng and Sun (2015) |
|  | *B. thuringiensis* | G25-53 | maybe soil | LDFU01002192 | USA | 2011 | Zheng and Sun (2015) |
|  | *B. thuringiensis* | G25-6 | unknown | LPZW01000120.1 | China | 2011 | Zheng and Sun (2015) |
|  | *B. thuringiensis* | G25-76 | soil | LDGN01000041.1 | Germany | 2011 | Zheng and Sun (2015) |
|  | *B. thuringiensis* | G25-78 | soil | LDGP01000199.1 | Germany | 2011 | Zheng and Sun (2015) |
|  | *B. thuringiensis* | G25-85 | horse dung | LDGU01000059.1 | Germany | 2011 | Zheng and Sun (2015) |
|  | *B. thuringiensis* | G25-87 | horse dung | LDGW01000191.1 | Germany | 2011 | Zheng and Sun (2015) |
|  | *B. thuringiensis* | G25-9 | soil | LPZX01000139.1 | China | 2011 | Zheng and Sun (2015) |
|  | *B. thuringiensis* | G25-91 | dung bettles | LDJT01000107.1 | Germany | 2011 | Zheng and Sun (2015) |
|  | *B. thuringiensis* | G25-93 | soil | LDHB01000188.1 | USA | 2011 | Zheng and Sun (2015) |
|  | *B. thuringiensis* | G25-94 | soil | LDHC01000019.1 | USA | 2011 | Zheng and Sun (2015) |
|  | *B. thuringiensis* | G25-95 | soil | LDHD01000005.1 | USA | 2011 | Zheng and Sun (2015) |
|  | *B. thuringiensis* | G25-96 | soil | LDHE01000022.1 | USA | 2011 | Zheng and Sun (2015) |
|  | *B. thuringiensis* | G25-97 | soil | LDHF01000169.1 | USA | 2011 | Zheng and Sun (2015) |
|  | *B. thuringiensis* | G25-98 | soil | LDHG01000029.1 | Spain | 2011 | Zheng and Sun (2015) |
|  | *B. thuringiensis* | GA-A07 | zebrafish tank detritus | CP042270 | Taiwan | 2018 | Chang et al. (2019) |
|  | *B. thuringiensis* | GBAC46 | grass roots | PVRM01000031.1 | China | ‎2005‎ | Borriss et al. (2018)‎ |
|  | *B. thuringiensis* | Strain GC_91 isolate 18-617 | pesticide | DAOIWX010000033.1 | France | 2018 | Souvorov et al. (2018) |
|  | *B. thuringiensis* | GC-91 | Biopesticide Agree WP | CP083144 | unknown | 2017 | Biggel (2021) |
|  | *B. thuringiensis* | gfu2-1 | soil | DAOJIF010000019.1 | Japan | 2011 | Souvorov et al. (2018) |
|  | *B. thuringiensis* | GOE1 | tomato rhizosphere | LXLF01000019.1 | Germay | 2014 | Liesegang (2016) |
|  | *B. thuringiensis* | GOE2 | tomato rhizosphere | LXLG01000028.1 | Germay | 2014 | Liesegang (2016) |
|  | *B. thuringiensis* | GOE3 | tomato rhizosphere | LXLH01000043.1 | Germay | 2014 | Liesegang (2016) |
|  | *B. thuringiensis* | GOE4 | tomato rhizosphere | LXLI01000038.1 | Germay | 2014 | Liesegang (2016) |
|  | *B. thuringiensis* | GOE5 | tomato rhizosphere | LXLJ01000001.1 | Germany | 2014 | Liesegang (2016) |
|  | *B. thuringiensis* | GOE7 | tomato rhizosphere | LXLL01000001.1 | Germany | 2014 | Liesegang (2016)‎ |
|  | *B. thuringiensis* | GPL-O901 | unknown | ML762307.1 | unknown | unknown | Geng et al. (2019) |
|  | *B. thuringiensis* | GR007 | farmland | CP076539 | Mexico | 2007 | Pacheco et al. (2021) |
|  | *B. thuringiensis* | h16 | unknown | MOOU01000116.1 | unknown | unknown | Zheng et al. (2016) |
|  | *B. thuringiensis* | H3 | soil | CP052061.1 | Lebanon | 2010 | Fayad et al. (2020) |
|  | *B. thuringiensis* | HBF-18 | soil | NHNS01000050.1 | China | 2003 | Shu (2017) |
|  | *B. thuringiensis* | HD 1 | Insect larvae | CP010005.1 | unknown | 2000 | Johnson et al. (2015) |
|  | *B. thuringiensis* | HD 600 | silkworm | JTHH01000008.1 | Japan | 2007 | Bishop-Lilly et al. (2014) |
|  | *B. thuringiensis* | HD-1 | Pectinophora gossypiella | JMHW01000166.1 | USA | 1970 | Dyer et al. (2014) |
|  | *B. thuringiensis* | HD1002 | Sewage | CP009351.1 | Israel | unknown | Johnson et al. (2015) |
|  | *B. thuringiensis* | HD1011 | unknown | CP009335.1 | India | 1915 | Bishop-Lilly et al. (2014) |
|  | *B. thuringiensis* | HD12 | soil | CP014847 | USA | 2012 | Shu (2016) |
|  | *B. thuringiensis* | HD133 | Plodia interpunctella | NHZG01000048.1 | United Kingdom | 1993 | Fu et al. (2017) |
|  | *B. thuringiensis* | serovar aizawai strain HD-133 | Plodia interpunctella | MOXQ01000047.1 | unknown | unknown | Jeong et al. (2016) |
|  | *B. thuringiensis* | HD-29 | *Dendrolimus sibiricus* | CP010089.1 | Czechoslovakia | 1970 | Zhu et al. (2014) |
|  | *B. thuringiensis* | HD5 | unknown | NFEM01000095.1 | unknown | unknown | Zheng et al. (2016) |
|  | *B. thuringiensis* | HD521 | soil | CP010106.1 | USA | 2009 | Li et al. (2015) |
|  | *B. thuringiensis* | HD571 | unknown | CP009600.1 | unknown | unknown | Bishop-Lilly et al. (2014) |
|  | *B. thuringiensis* | HD682 | unknown | CP009720.1 | unknown | unknown | Bishop-Lilly et al. (2014) |
|  | *B. thuringiensis* | HD73 | unknown | CP004069.1 | unknown | unknown | Liu et al. ( 2013) |
|  | *B. thuringiensis* | HD-771 | unknown | CP003752.1 | unknown | unknown | Doggett et al. (2012) |
|  | *B. thuringiensis* | HD-789 | unknown | CP003763.1 | unknown | unknown | Doggett et al. (2012) |
|  | *B. thuringiensis* | HER1410 | unknown | CP050183.1 | Canada | 1973 | Lood and Lechuga (2020) |
|  | *B. thuringiensis* | HM-311 | soil | CP040782 | China | 2018 | Zuo et al. (2019) |
|  | *B. thuringiensis* | HS18-1 | soil | CP012099 | China | 2009 | Zheng (2015) |
|  | *B. thuringiensis* | HSY204 | soil | JAHXRX010000052.1 | China | 2016 | Zhang (2021) |
|  | *B. thuringiensis* | Hu4-2 | dust sample from maize grain silo | AMXT00000000 | Spain | unknown | Palma and Caballero (2013) |
|  | *B. thuringiensis* | I13 | unknown | NFEN01000198.1 | unknown | unknown | Zheng et al. (2016) |
|  | *B. thuringiensis* | IBL 200 | unknown | CM000758.1 | unknown | unknown | Read et al. (2009)‎ |
|  | *B. thuringiensis* | IBL 4222 | unknown | CM000759.1 | unknown | unknown | Read et al. (2009)‎ |
|  | *B. thuringiensis* | IBL00055 | soil | JAMXJQ010000001.1 | USA | 1983 | Blackburn et al. (2022) |
|  | *B. thuringiensis* | IBL00090 | soil | JAMXJR010000001.1 | USA | 1983 | Blackburn et al. (2022) |
|  | *B. thuringiensis* | IBL00144 | soil | JAMXJS010000003.1 | USA | 1983 | Blackburn et al. (2022) |
|  | *B. thuringiensis* | IBL00171 | soil | JAMXJT010000001.1 | USA | 1983 | Blackburn et al. (2022) |
|  | *B. thuringiensis* | IBL00197 | soil | JAMXJU010000016.1 | USA | 1986 | Blackburn et al. (2022) |
|  | *B. thuringiensis* | IBL00210 | soil | JAMXJV010000001.1 | USA | 1986 | Blackburn et al. (2022) |
|  | *B. thuringiensis* | IBL00427 | soil | JAMXJW010000001.1 | Norway | 1986 | Blackburn et al. (2022) |
|  | *B. thuringiensis* | IBL00503 | soil | JAMXJX010000002.1 | USA | 1988 | Blackburn et al. (2022) |
|  | *B. thuringiensis* | IBL01259 | soil | JAMXJZ010000001.1 | Argentina | 1988 | Blackburn et al. (2022) |
|  | *B. thuringiensis* | IBL01313 | soil | JAMXKA010000001.1 | USA | 1989 | Blackburn et al. (2022) |
|  | *B. thuringiensis* | IBL01677 | soil | JAMXKB010000002.1 | USA | 1983 | Blackburn et al. (2022) |
|  | *B. thuringiensis* | IBL02897 | soil | JAMXKC010000001.1 | Viet Nam | 1988 | Blackburn et al. (2022) |
|  | *B. thuringiensis* | IBL03111 | soil | JAMXKD020000003.1 | USA | 1983 | Blackburn et al. (2022) |
|  | *B. thuringiensis* | IEBC_T61001 | unknown | FMBI01000058.1 | unknown | unknown | Loux and Rue (2016) |
|  | *B. thuringiensis* | IIF1SG-B5 | unknown | JABVDE010000003.1 | USA | 2015 | Daudu et al. (2020) |
|  | *B. thuringiensis* | IIF2SG-B1 | unknown | JABVDL010000003.1 | USA | 2015 | Daudu et al. (2020) |
|  | *B. thuringiensis* | IIF3SG-B3 | unknown | JABVDF010000004.1 | USA | 2015 | Daudu et al. (2020) |
|  | *B. thuringiensis* | IIF4SW-B1 | unknown | JABVDJ010000003.1 | USA | 2015 | Daudu et al. (2020) |
|  | *B. thuringiensis* | IIF4SW-B2 | unknown | JABVDG010000003.1 | USA | 2015 | Daudu et al. (2020) |
|  | *B. thuringiensis* | IIF4SW-B3 | unknown | JABVDK010000003.1 | USA | 2015 | Daudu et al. (2020) |
|  | *B. thuringiensis* | IIF4SW-P2 | unknown | JABVDN010000004.1 | USA | 2015 | Daudu et al. (2020) |
|  | *B. thuringiensis* | IIF4SW-P3 | unknown | JABVDI010000003.1 | USA | 2015 | Daudu et al. (2020) |
|  | *B. thuringiensis* | IIF4SW-P4 | unknown | JABVDH010000003.1 | USA | 2015 | Daudu et al. (2020) |
|  | *B. thuringiensis* | IIF4SW-P5 | unknown | JABVDM010000004.1 | USA | 2015 | Daudu et al. (2020) |
|  | *B. thuringiensis* | IIF7SG-B4 | unknown | JABVDO010000003.1 | USA | 2015 | Daudu et al. (2020) |
|  | *B. thuringiensis* | IMBL-B9 | unknown | CP072691.1 | South Korea | 2018 | Park et al. (2022) |
|  | *B. thuringiensis* | INTA Fr7-4 | soil | MSFC01000001.1 | Argentina | unknown | Navas et al. (2016) |
|  | *B. thuringiensis* | INTA Mo4-4 | Stored product dust | RIBV01000009.1 | Argentina | 1998 | Sauka et al. (2018) |
|  | *B. thuringiensis* | IPPBIOTSUC1012 | soil | NTGL01000005.1 | China | 2011 | Liu et al. (2017) |
|  | *B. thuringiensis* | IPPBIOTSUC-C9F1 | soil | QKVU01000033.1 | China | 2014 | Wang et al. (2018) |
|  | *B. thuringiensis* | IS5056 | unknown | CP004123.1 | unknown | unknown | Murawska et al. (2013) |
|  | *B. thuringiensis* | isolate 07_29 | food | DAOIYM010000044.1 | France | 2007 | Souvorov et al. (2018) |
|  | *B. thuringiensis* | isolate 07_30 | food | DAOIYK010000039.1 | France | 2008 | Souvorov et al. (2018) |
|  | *B. thuringiensis* | isolate 07_31 | food | DAOIYI010000039.1 | France | 2007 | Souvorov et al. (2018) |
|  | *B. thuringiensis* | isolate 07_32 | food | DAOIYJ010000042.1 | France | 2007 | Souvorov et al. (2018) |
|  | *B. thuringiensis* | isolate 08_037 | food | DAOJAU010000043.1 | France | 2008 | Souvorov et al. (2018) |
|  | *B. thuringiensis* | isolate 08_121 | food | DAOIYG010000041.1 | France | 2008 | Souvorov et al. (2018) |
|  | *B. thuringiensis* | isolate 08_122 | food | DAOIYH010000049.1 | France | 2008 | Souvorov et al. (2018) |
|  | *B. thuringiensis* | isolate 08_123 | food | DAOIYD010000044.1 | France | 2008 | Souvorov et al. (2018) |
|  | *B. thuringiensis* | isolate 08_124 | food | DAOIYF010000047.1 | France | 2008 | Souvorov et al. (2018) |
|  | *B. thuringiensis* | isolate 08_126 | food | DAOIYN010000044.1 | France | 2008 | Souvorov et al. (2018) |
|  | *B. thuringiensis* | isolate 08_127 | food | DAOJAY010000050.1 | France | 2008 | Souvorov et al. (2018) |
|  | *B. thuringiensis* | isolate 08_128 | food | DAOIWC010000001.1 | France | 2008 | Souvorov et al. (2018) |
|  | *B. thuringiensis* | isolate 08_135 | food | DAOJBA010000049.1 | France | 2008 | Souvorov et al. (2018) |
|  | *B. thuringiensis* | isolate 08_138 | food | DAOJAZ010000044.1 | France | 2008 | Souvorov et al. (2018) |
|  | *B. thuringiensis* | isolate 08_38 | food | DAOJAV010000041.1 | France | 2008 | Souvorov et al. (2018) |
|  | *B. thuringiensis* | isolate 08_74 | food | DAOJAW010000049.1 | France | 2008 | Souvorov et al. (2018) |
|  | *B. thuringiensis* | isolate 08_89 | food | DAOJAS010000044.1 | France | 2008 | Souvorov et al. (2018) |
|  | *B. thuringiensis* | isolate 09_68 | food | DAOIYT010000042.1 | France | 2009 | Souvorov et al. (2018) |
|  | *B. thuringiensis* | isolate 09_69 | food | DAOIYV010000041.1 | France | 2009 | Souvorov et al. (2018) |
|  | *B. thuringiensis* | isolate 10_01 | food | DAOIYU010000043.1 | France | 2010 | Souvorov et al. (2018) |
|  | *B. thuringiensis* | isolate 10_02 | food | DAOIYP010000044.1 | France | 2010 | Souvorov et al. (2018) |
|  | *B. thuringiensis* | isolate 10_03 | food | DAOIYO010000047.1 | France | 2010 | Souvorov et al. (2018) |
|  | *B. thuringiensis* | isolate 10_04 | food | DAOIYR010000051.1 | France | 2010 | Souvorov et al. (2018) |
|  | *B. thuringiensis* | isolate 10_05 | food | DAOIYQ010000099.1 | France | 2010 | Souvorov et al. (2018) |
|  | *B. thuringiensis* | isolate 10_46 | food | DAOIYW010000049.1 | France | 2010 | Souvorov et al. (2018) |
|  | *B. thuringiensis* | isolate 10_47 | food | DAOIZM010000045.1 | France | 2010 | Souvorov et al. (2018) |
|  | *B. thuringiensis* | isolate 10_48 | food | DAOIZP010000046.1 | France | 2010 | Souvorov et al. (2018) |
|  | *B. thuringiensis* | isolate 10_49 | food | DAOIZN010000043.1 | France | 2010 | Souvorov et al. (2018) |
|  | *B. thuringiensis* | isolate 10_50 | food | DAOIZO010000044.1 | France | 2010 | Souvorov et al. (2018) |
|  | *B. thuringiensis* | isolate 10_51 | food | DAOIZK010000044.1 | France | 2010 | Souvorov et al. (2018) |
|  | *B. thuringiensis* | isolate 10CEB11 | Canteen control | DAOIJC010000041.1 | France | 2010 | Souvorov et al. (2018) |
|  | *B. thuringiensis* | isolate 10CEB12 | Canteen control | DAOIJD010000041.1 | France | 2010 | Souvorov et al. (2018) |
|  | *B. thuringiensis* | isolate 11_48 | food | DAOIZL010000047.1 | France | 2011 | Souvorov et al. (2018) |
|  | *B. thuringiensis* | isolate 12_17 | food | DAOIZI010000045.1 | France | 2012 | Souvorov et al. (2018) |
|  | *B. thuringiensis* | isolate 14_08 | food | DAOIZJ010000048.1 | France | 2011 | Souvorov et al. (2018) |
|  | *B. thuringiensis* | isolate 14_16 | food | DAOIZQ010000045.1 | France | 2011 | Souvorov et al. (2018) |
|  | *B. thuringiensis* | isolate 14_176 | food | DAOIXU010000043.1 | France | 2012 | Souvorov et al. (2018) |
|  | *B. thuringiensis* | isolate 14_177 | food | DAOIXR010000048.1 | France | 2012 | Souvorov et al. (2018) |
|  | *B. thuringiensis* | isolate 14_178 | food | DAOIXP010000048.1 | France | 2012 | Souvorov et al. (2018) |
|  | *B. thuringiensis* | isolate 14_179 | food | DAOIXO010000045.1 | France | 2012 | Souvorov et al. (2018) |
|  | *B. thuringiensis* | isolate 14_18 | food | DAOIXN010000049.1 | France | 2011 | Souvorov et al. (2018) |
|  | *B. thuringiensis* | isolate 14_20 | food | DAOIXM010000047.1 | France | 2011 | Souvorov et al. (2018) |
|  | *B. thuringiensis* | isolate 14_22 | food | DAOIXL010000047.1 | France | 2011 | Souvorov et al. (2018) |
|  | *B. thuringiensis* | isolate 14_262 | food | DAOIXT010000050.1 | France | 2012 | Souvorov et al. (2018) |
|  | *B. thuringiensis* | isolate 14_263 | food | DAOIXW010000048.1 | France | 2012 | Souvorov et al. (2018) |
|  | *B. thuringiensis* | isolate 14_264 | food | DAOIXS010000049.1 | France | 2012 | Souvorov et al. (2018) |
|  | *B. thuringiensis* | isolate 14_265 | food | DAOIXX010000044.1 | France | 2012 | Souvorov et al. (2018) |
|  | *B. thuringiensis* | isolate 14_309 | food | DAOIXV010000043.1 | France | 2013 | Souvorov et al. (2018) |
|  | *B. thuringiensis* | isolate 14_310 | food | DAOIXY010000049.1 | France | 2013 | Souvorov et al. (2018) |
|  | *B. thuringiensis* | isolate 14_311 | food | DAOIXZ010000046.1 | France | 2013 | Souvorov et al. (2018) |
|  | *B. thuringiensis* | isolate 14_312 | food | DAOIYB010000045.1 | France | 2013 | Souvorov et al. (2018) |
|  | *B. thuringiensis* | isolate 14_313 | food | DAOIYC010000048.1 | France | 2013 | Souvorov et al. (2018) |
|  | *B. thuringiensis* | isolate 14_361 | food | DAOIYE010000041.1 | France | 2013 | Souvorov et al. (2018) |
|  | *B. thuringiensis* | isolate 14_362 | food | DAOIWQ010000041.1 | France | 2013 | Souvorov et al. (2018) |
|  | *B. thuringiensis* | isolate 14_364 | food | DAOIWP010000047.1 | France | 2013 | Souvorov et al. (2018) |
|  | *B. thuringiensis* | isolate 14_370 | food | DAOIWS010000048.1 | France | 2013 | Souvorov et al. (2018) |
|  | *B. thuringiensis* | isolate 14_371 | food | DAOIWR010000048.1 | France | 2013 | Souvorov et al. (2018) |
|  | *B. thuringiensis* | isolate 14_372 | food | DAOIWU010000042.1 | France | 2013 | Souvorov et al. (2018) |
|  | *B. thuringiensis* | isolate 14_373 | food | DAOIWT010000046.1 | France | 2013 | Souvorov et al. (2018) |
|  | *B. thuringiensis* | isolate 14_374 | food | DAOIWW010000050.1 | France | 2013 | Souvorov et al. (2018) |
|  | *B. thuringiensis* | isolate 14_388 | food | DAOIWV010000042.1 | France | 2013 | Souvorov et al. (2018) |
|  | *B. thuringiensis* | isolate 15_1007 | food | DAOIWO010000041.1 | France | 2015 | Souvorov et al. (2018) |
|  | *B. thuringiensis* | isolate 15_1008 | food | DAOIWM010000046.1 | France | 2015 | Souvorov et al. (2018) |
|  | *B. thuringiensis* | isolate 15_1009 | food | DAOIXD010000042.1 | France | 2015 | Souvorov et al. (2018) |
|  | *B. thuringiensis* | isolate 15_1010 | food | DAOIXE010000043.1 | France | 2015 | Souvorov et al. (2018) |
|  | *B. thuringiensis* | isolate 15_1011 | food | DAOIXC010000041.1 | France | 2015 | Souvorov et al. (2018) |
|  | *B. thuringiensis* | isolate 15_1331 | food | DAOIXH010000041.1 | France | 2015 | Souvorov et al. (2018) |
|  | *B. thuringiensis* | isolate 15_482 | food | DAOIXI010000044.1 | France | 2015 | Souvorov et al. (2018) |
|  | *B. thuringiensis* | isolate 15_598 | food | DAOIXF010000045.1 | France | 2015 | Souvorov et al. (2018) |
|  | *B. thuringiensis* | isolate 15_599 | food | DAOIXG010000047.1 | France | 2015 | Souvorov et al. (2018) |
|  | *B. thuringiensis* | isolate 15_600 | food | DAOIWZ010000048.1 | France | 2015 | Souvorov et al. (2018) |
|  | *B. thuringiensis* | isolate 15_601 | food | DAOIXA010000045.1 | France | 2015 | Souvorov et al. (2018) |
|  | *B. thuringiensis* | isolate 15_602 | food | DAOJAG010000045.1 | France | 2015 | Souvorov et al. (2018) |
|  | *B. thuringiensis* | isolate 15_603 | food | DAOJAD010000047.1 | France | 2015 | Souvorov et al. (2018) |
|  | *B. thuringiensis* | isolate 15_605 | food | DAOJAE010000046.1 | France | 2015 | Souvorov et al. (2018) |
|  | *B. thuringiensis* | isolate 15_606 | food | DAOJAB010000045.1 | France | 2015 | Souvorov et al. (2018) |
|  | *B. thuringiensis* | isolate 15_607 | food | DAOJAJ010000044.1 | France | 2015 | Souvorov et al. (2018) |
|  | *B. thuringiensis* | isolate 15_915 | food | DAOJAF010000047.1 | France | 2015 | Souvorov et al. (2018) |
|  | *B. thuringiensis* | isolate 15_93 | food | DAOJAC010000041.1 | France | 2015 | Souvorov et al. (2018) |
|  | *B. thuringiensis* | isolate 16_1122 | food | DAOJAA010000038.1 | France | 2016 | Souvorov et al. (2018) |
|  | *B. thuringiensis* | isolate 16_1310 | food | DAOJBF010000043.1 | France | 2016 | Souvorov et al. (2018) |
|  | *B. thuringiensis* | isolate 16_1549 | food | DAOJBG010000040.1 | France | 2016 | Souvorov et al. (2018) |
|  | *B. thuringiensis* | isolate 16_1643 | food | DAOJBK010000039.1 | France | 2016 | Souvorov et al. (2018) |
|  | *B. thuringiensis* | isolate 16_350 | food | DAOJBB010000043.1 | France | 2016 | Souvorov et al. (2018) |
|  | *B. thuringiensis* | isolate 16_372 | food | DAOJBC010000045.1 | France | 2016 | Souvorov et al. (2018) |
|  | *B. thuringiensis* | isolate 16_379 | food | DAOJBD010000040.1 | France | 2016 | Souvorov et al. (2018) |
|  | *B. thuringiensis* | isolate 16_380 | food | DAOJBE010000041.1 | France | 2016 | Souvorov et al. (2018) |
|  | *B. thuringiensis* | isolate 16_381 | food | DAOJBJ010000046.1 | France | 2016 | Souvorov et al. (2018) |
|  | *B. thuringiensis* | isolate 16_417 | food | DAOIZX010000041.1 | France | 2016 | Souvorov et al. (2018) |
|  | *B. thuringiensis* | isolate 16_418 | food | DAOIZV010000043.1 | France | 2016 | Souvorov et al. (2018) |
|  | *B. thuringiensis* | isolate 16_440 | food | DAOIZZ010000042.1 | France | 2016 | Souvorov et al. (2018) |
|  | *B. thuringiensis* | isolate 16_670 | food | DAOIZY010000043.1 | France | 2016 | Souvorov et al. (2018) |
|  | *B. thuringiensis* | isolate 16_898 | food | DAOIZT010000043.1 | France | 2016 | Souvorov et al. (2018) |
|  | *B. thuringiensis* | isolate 16SBCL1559 | food | DAOIJO010000042.1 | France | 2016 | Souvorov et al. (2018) |
|  | *B. thuringiensis* | isolate 16SBCL1560 | food | DAOIJP010000041.1 | France | 2016 | Souvorov et al. (2018) |
|  | *B. thuringiensis* | isolate 16SBCL437 | food | DAOIJR010000044.1 | France | 2016 | Souvorov et al. (2018) |
|  | *B. thuringiensis* | isolate 16SBCL438 | food | DAOIJQ010000040.1 | France | 2016 | Souvorov et al. (2018) |
|  | *B. thuringiensis* | isolate 16SBCL439 | food | DAOIJS010000044.1 | France | 2016 | Souvorov et al. (2018) |
|  | *B. thuringiensis* | isolate 17_1202 | food | DAOIZW010000045.1 | France | 2017 | Souvorov et al. (2018) |
|  | *B. thuringiensis* | isolate 17_263 | food | DAOIZS010000038.1 | France | 2017 | Souvorov et al. (2018) |
|  | *B. thuringiensis* | isolate 17_264 | food | DAOIZR010000041.1 | France | 2017 | Souvorov et al. (2018) |
|  | *B. thuringiensis* | isolate 17_265 | food | DAOIZE010000039.1 | France | 2017 | Souvorov et al. (2018) |
|  | *B. thuringiensis* | isolate 17_266 | food | DAOIZH010000038.1 | France | 2017 | Souvorov et al. (2018) |
|  | *B. thuringiensis* | isolate 17_267 | food | DAOIZF010000040.1 | France | 2017 | Souvorov et al. (2018) |
|  | *B. thuringiensis* | isolate 17_268 | food | DAOIZG010000033.1 | France | 2017 | Souvorov et al. (2018) |
|  | *B. thuringiensis* | isolate 17_334 | food | DAOIZC010000043.1 | France | 2017 | Souvorov et al. (2018) |
|  | *B. thuringiensis* | isolate 17_429 | food | DAOIZD010000101.1 | France | 2017 | Souvorov et al. (2018) |
|  | *B. thuringiensis* | isolate 17_430 | food | DAOIZB010000042.1 | France | 2017 | Souvorov et al. (2018) |
|  | *B. thuringiensis* | isolate 17_527 | food | DAOIYZ010000038.1 | France | 2017 | Souvorov et al. (2018) |
|  | *B. thuringiensis* | isolate 17_528 | food | DAOIYY010000042.1 | France | 2017 | Souvorov et al. (2018) |
|  | *B. thuringiensis* | isolate 17_529 | food | DAOIZA010000038.1 | France | 2017 | Souvorov et al. (2018) |
|  | *B. thuringiensis* | isolate 17_530 | food | DAOIWK010000037.1 | France | 2017 | Souvorov et al. (2018) |
|  | *B. thuringiensis* | isolate 17_531 | food | DAOIWJ010000040.1 | France | 2017 | Souvorov et al. (2018) |
|  | *B. thuringiensis* | isolate 17_619 | food | DAOIWI010000040.1 | France | 2017 | Souvorov et al. (2018) |
|  | *B. thuringiensis* | isolate 17_620 | food | DAOIWH010000035.1 | France | 2017 | Souvorov et al. (2018) |
|  | *B. thuringiensis* | isolate 17_621 | food | DAOIWG010000043.1 | France | 2017 | Souvorov et al. (2018) |
|  | *B. thuringiensis* | isolate 17_622 | food | DAOIWE010000041.1 | France | 2017 | Souvorov et al. (2018) |
|  | *B. thuringiensis* | isolate 17_623 | food | DAOIWF010000043.1 | France | 2017 | Souvorov et al. (2018) |
|  | *B. thuringiensis* | isolate 17_885 | food | DAOIWD010000041.1 | France | 2017 | Souvorov et al. (2018) |
|  | *B. thuringiensis* | isolate 17_967 | food | DAOIWN010000041.1 | France | 2017 | Souvorov et al. (2018) |
|  | *B. thuringiensis* | isolate 17_968 | food | DAOJAK010000038.1 | France | 2017 | Souvorov et al. (2018) |
|  | *B. thuringiensis* | isolate 17_969 | food | DAOJAM010000042.1 | France | 2017 | Souvorov et al. (2018) |
|  | *B. thuringiensis* | isolate 17_970 | food | DAOJAL010000035.1 | France | 2017 | Souvorov et al. (2018) |
|  | *B. thuringiensis* | isolate 17_971 | food | DAOJAO010000036.1 | France | 2017 | Souvorov et al. (2018) |
|  | *B. thuringiensis* | isolate CH048 | unknown | DAOJEQ010000026.1 | unknown | unknown | Souvorov et al. (2018) |
|  | *B. thuringiensis* | isolate CH060 | unknown | DAOJEX010000002.1 | unknown | unknown | Souvorov et al. (2018) |
|  | *B. thuringiensis* | isolate CH078 | unknown | DAOJFC010000001.1 | unknown | unknown | Souvorov et al. (2018) |
|  | *B. thuringiensis* | isolate CH187 | unknown | DAOICT010000006.1 | unknown | unknown | Souvorov et al. (2018) |
|  | *B. thuringiensis* | isolate CH188 | unknown | DAOICU010000033.1 | unknown | unknown | Souvorov et al. (2018) |
|  | *B. thuringiensis* | isolate CH442 | unknown | DAOJDR010000001.1 | unknown | unknown | Souvorov et al. (2018) |
|  | *B. thuringiensis* | isolate ISI 2824 | Water kefir | CAKJXA010000017.1 | unknown | unknown | Metto 2021 |
|  | *B. thuringiensis* | isolate MicBio3 | soil | DAOIPC010000067.1 | spain | 2014 | Souvorov et al. (2018) |
|  | *B. thuringiensis* | isolate MicBio39 | soil | DAOIPA010000042.1 | Spain | 2014 | Souvorov et al. (2018) |
|  | *B. thuringiensis* | isolate MicBio56 | soil | DAOIPB010000032.1 | Spain | 2014 | Souvorov et al. (2018) |
|  | *B. thuringiensis* | isolate pediatric patient SAMN36761899-rid18975253 | human blood culture | DAPQOA010000050.1 | USA | 2006 | Souvorov et al. (2018) |
|  | *B. thuringiensis* | isolate pediatric patient SAMN36761911-rid18976983 | human blood culture | DAPQOC010000046.1 | USA | 2007 | Souvorov et al. (2018) |
|  | *B. thuringiensis* | isolate pediatric patient SAMN36761918-rid18975223 | human blood culture | DAPQOD010000049.1 | USA | 2008 | Souvorov et al. (2018) |
|  | *B. thuringiensis* | Patient-SAMN36761919 | human blood culture | DAPQOF010000041 | USA | 2008 | Souvorov et al. (2018) |
|  | *B. thuringiensis* | isolate pediatric patient SAMN36761922-rid18975193 | human blood culture | DAPQOH010000005.1 | USA | 2008 | Souvorov et al. (2018) |
|  | *B. thuringiensis* | isolate pediatric patient SAMN36761928-rid18975183 | human blood culture | DAPQOJ010000012.1 | USA | 2009 | Souvorov et al. (2018) |
|  | *B. thuringiensis* | isolate pediatric patient SAMN36761931-rid18976803 | human blood culture | DAPQOX010000028.1 | USA | 2010 | Souvorov et al. (2018) |
|  | *B. thuringiensis* | isolate pediatric patient SAMN36761936-rid18975073 | human blood culture | DAPQPD010000033.1 | USA | 2010 | Souvorov et al. (2018) |
|  | *B. thuringiensis* | isolate pediatric patient SAMN36761952-rid18976933 | human blood culture | DAPQOL010000004.1 | USA | 2007 | Souvorov et al. (2018) |
|  | *B. thuringiensis* | isolate pediatric patient SAMN36761955-rid18975163 | human blood culture | DAPQOM010000057.1 | USA | 2008 | Souvorov et al. (2018) |
|  | *B. thuringiensis* | isolate pediatric patient SAMN36761963-rid18976873 | human blood culture | DAPQOT010000031.1 | USA | 2010 | Souvorov et al. (2018) |
|  | *B. thuringiensis* | isolate pediatric patient SAMN36761975-rid18976733 | human blood culture | DAPQPK010000012.1 | USA | 2012 | Souvorov et al. (2018) |
|  | *B. thuringiensis* | isolate pediatric patient SAMN36761987-rid18975013 | human cerebrospinal fluid culture | DAPQPL010000036.1 | USA | unknown | Souvorov et al. (2018) |
|  | *B. thuringiensis* | isolate pediatric patient SAMN36761992-rid18976683 | human cerebrospinal fluid culture | DAPQPM010000005.1 | USA | unknown | Souvorov et al. (2018) |
|  | *B. thuringiensis* | isolate UBA3967 | wood | DGCX01000012.1 | USA | unknown | Park et al. (2017) |
|  | *B. thuringiensis* | IZSPB_BC56B | Ice-cream | JAJESR010000023.1 | Italy | 2018 | Parisi et al. (2021) |
|  | *B. thuringiensis* | JM-Mgvxx-63 | mangrove sediment | AYSM01000033.1 | Brazil | 2013 | Marcon et al. (2014) |
|  | *B. thuringiensis* | JRO03 | soil | JAHTLE010000004.1 | USA | 2021 | Breton et al. (2021) |
|  | *B. thuringiensis* | JW-1 | soil | CP045030 | China | 2015 | Ma and Wang (2019) |
|  | *B. thuringiensis* | K2-sn1398 | wastwater | JARTVI010000002.1 | Denmark | 2017 | Roder and Maccario (2023) |
|  | *B. thuringiensis* | KF1 | soil | CP085409.1 | China | 2016 | Sun (2021) |
|  | *B. thuringiensis* | kn11 | soil | QNUR01000044.1 | China | 2017 | Shu et al. (2018) |
| 1. - | *B. thuringiensis* | KNU_07 | Ginseng | CP016588 | South Korea | 2015 | Park et al. (2016) |
|  | *B. thuringiensis* | KNU-25 | soil | JABXXL010000003.1 | South Korea | 2020 | Jung et al. (2020) |
|  | *B. thuringiensis* | KNU-26 | soil | JABXXM010000008.1 | South Korea | 2020 | Jung et al. (2020) |
|  | *B. thuringiensis* | L1 | Coprinus comatus | CP132200.1 | China | 2019 | Chen (2023) |
|  | *B. thuringiensis* | L-7601 | unknown | CP020002 | China | 2015 | Cai et al. (2017) |
|  | *B. thuringiensis* | Leapi01 | Mythimna loreyi | NZ_AMXS00000000 | Spain | unknown | Palma and Caballero (2013) |
|  | *B. thuringiensis* | Lip | soil | CP116313.1 | Lebanon | 2013 | Fayad et al. (2023) |
|  | *B. thuringiensis* | LM1212 | cadaver of an Oryctes gigas larva | AYPV01000027.1 | Madagascar | unknown | Song et al. (2013) |
|  | *B. thuringiensis* | LP_1_YM | unknown | SMDF01000037.1 | USA | unknown | Frank et al. (2019) |
|  | *B. thuringiensis* | LP_2_YM | unknown | SMDG01000036.1 | USA | unknown | Frank et al. (2019) |
|  | *B. thuringiensis* | Lr3/2 | water | JYCH01000001.1 | Chile | 2011 | Filippidou et al. (2014) |
|  | *B. thuringiensis* | Lr7/2 | water | JYCL01000035.1 | Chile | 2011 | Filippidou et al. (2014) |
|  | ***B. thuringiensis*** | **LTD162** | **Technical Material of Biopesticide** | **JAJMUD010000015.1** | **China** | **2019** | **Yue (2021)** |
|  | *B. thuringiensis* | LX43 | soil | Cp094624 | China | 2021 | Xin and Liu, 2022 |
|  | *B. thuringiensis* | m401 | honey | PYAP02000027.1 | Argentina | 2001 | Lamelza et al. (2020) |
|  | *B. thuringiensis* | MC28 | unknown | CP003687.1 | unknown | unknown | Zheng et al. (2012) |
|  | *B. thuringiensis* | MGYG-HGUT-01712 | human gut | CABLCE010000001.1 | unknown | unknown | EMBL-EBI Metagenomics Team (2019) |
|  | *B. thuringiensis* | Monterrey_S4 | soil | JAVIVV010000003.1 | Mexico | 2013 | Song et al. (2023) |
|  | *B. thuringiensis* | MORW_BS1.1 | root-soil zone | JAASGY010000092.1 | South Africa | 2015 | Adeniji (2020) |
|  | *B. thuringiensis* | MS12 | Mixed salad | SJQC01000035.1 | Germany | 2015 | Fiedler et al. (2019) |
|  | *B. thuringiensis* | MS532a | Mixed salad | SJQG01000010.1 | Germany | 2015 | Fiedler et al. (2019) |
|  | *B. thuringiensis* | MW | freshwater stream | SUPP01000004.1 | USA | 2019 | Williams and Maclea (2019) |
|  | *B. thuringiensis* | MYBT18246 | Caenorhabditis elegans | Cp015350 | unknown | unknown | Hollensteiner et al. (2016) |
|  | *B. thuringiensis* | NA205-3 | dust from maize silo | AYXQ01000075.1 | Spain | unknown | Palma et al. (2014) |
|  | *B. thuringiensis* | NB_176 isolate 18_485 | pesticide | DAOIXJ010000004.1 | France | 2018 | Souvorov et al. (2018) |
|  | *B. thuringiensis* | NB125 | "Bacillus thuringiensis ssp. Tenebrionis (Btt)" | CP114392 | Germany | 2021 | Schafer et al. (2022) |
|  | *B. thuringiensis* | NB-176 | Biopesticide Novodor 3FC | CP083129 | unknown | 2017 | Biggel (2021) |
|  | *B. thuringiensis* | NB176-1 | "Bacillus thuringiensis ssp. Tenebrionis (Btt)" | CP114399.1 | Germany | 2021 | Schaefer et al. (2022) |
|  | *B. thuringiensis* | NBIN-866 | soil | KK088378.1 | China | unknown | Liu et al. (2014) |
|  | *B. thuringiensis* | NMTD81 | soil | PVRL01000004.1 | China | 2005 | Borriss et al. (2018) |
|  | *B. thuringiensis* | NR-28583 | unknown | VLZB01000002.1 | unknown | unknown | Riojas et al. (‎2019)‎ |
|  | *B. thuringiensis* | NR-610 | unknown | VLYZ01000087.1 | unknown | unknown | Riojas et al. (2019) |
|  | *B. thuringiensis* | NRRL 23135 | Culicidae | PGDT01000038.1 | United Kingdom | 2013 | Pascoe et al. (2017) |
|  | *B. thuringiensis* | NRRL B-23139 | soil | CP035727 | Russia | 2017 | Andreeva and Grigoreva (2020) |
|  | *B. thuringiensis* | NRRL B-23150 | Culicidae | PGSA01000142.1 | United Kingdom | 2013 | Pascoe et al. (2017) |
|  | *B. thuringiensis* | NRRL B23152 | Isoptera | PGDW01000144.1 | United Kingdom | 2013 | Pascoe et al. (2017) |
|  | *B. thuringiensis* | NT06 | soil | VLPN01000038.1 | Viet Nam | 2015 | Le et al. (2019) |
|  | *B. thuringiensis* | P01_1 | food | JAIVBV010000046.1 | unknown | 2012 | Biggel (2021) |
|  | *B. thuringiensis* | P01_3 | food | JAIVBU010000047.1 | unknown | unknown | Biggel (2021) |
|  | *B. thuringiensis* | Pasteur institute standard strain | unknown | AP014864 | unknown | unknown | Kanda et al. (2015) |
|  | *B. thuringiensis* | PS3 | rhizhosphere soil | CP130743.1 | China | 2019 | Geng et al. (2023) |
|  | *B. thuringiensis* | PT02 | soil | VLPO01000036.1 | Viet Nam | 2015 | Le et al. (2019) |
|  | *B. thuringiensis* | PT18 | soil | VLPP01000048.1 | Viet Nam | 2015 | Le et al. (2019) |
|  | *B. thuringiensis* | QB15 | soil | VLPQ01000038.1 | Viet Nam | 2015 | Le et al. (2019) |
|  | *B. thuringiensis* | QZL38 | soil | CP032608 | China | 2018 | Song (2018) |
|  | ***B. thuringiensis*** | **RC340** | **unknown** | **JARXOY010000002.1** | **USA** | **2020** | **Sullivan et al. (2023)** |
|  | *B. thuringiensis* | rc6 | Black rice | QGGE01000023.1 | India | 2015 | Potshangbam et al. (2018) |
|  | *B. thuringiensis* | RCT09 | soil | JAHTKZ010000005.1 | USA | 2021 | Howard et al. (2021) |
|  | *B. thuringiensis* | RM11(2019) | grass soil | WBOO01000005.1 | USA | 2018 | Mendoza et al. (2019) |
|  | *B. thuringiensis* | S1287 | soil | VLJD01000022.1 | Brazil | 1996 | Monnerat et al. (2019) |
|  | *B. thuringiensis* | S1307 | soil | VLJE01000004.1 | Brazil | 1996 | Monnerat et al. (2019) |
|  | ***B. thuringiensis*** | **s1783** | **Clay soil** | **JACYOE010000018.1** | **Brazil** | **2015** | **Monnerat et al. (2020)** |
|  | *B. thuringiensis* | S1905 | soil | VKNY01000133.1 | Brazil | 2002 | Monnerat et al. (2019) |
|  | *B. thuringiensis* | s1930 | Clay soil | JACYOF010000011.1 | Brazil | 2002 | Monnerat et al. (2020) |
|  | *B. thuringiensis* | S2122 | soil | VJWI01000235.1 | Brazil | 2004 | Monnerat et al. (2019) |
|  | *B. thuringiensis* | S2124 | soil | VJWJ01000032.1 | Brazil | 2004 | Monnerat et al. (2019) |
|  | *B. thuringiensis* | S2160-1 | soil | NSKZ01000007.1 | China | 2007 | Fang (2017) |
|  | *B. thuringiensis* | S2728 | Clay soil | JACYOC010000003.1 | Brazil | 2017 | Monnerat et al. (2020) |
|  | *B. thuringiensis* | s2744 | Clay soil | JACYOH010000027.1 |  | 2017 | Monnerat et al. (2020) |
|  | *B. thuringiensis* | S38 | mangrove soil | JAMXMK010000003.1 | Philippines | 2017 | Creencia (2022) |
|  | *B. thuringiensis* | S601 | soil | VFHY01000052.1 | Brazil | 1990 | Monnerat et al. (2019) |
|  | *B. thuringiensis* | S906 | soil | VIGY01000046.1 | Brazil | 1994 | Monnerat et al. (2019) |
|  | *B. thuringiensis* | S907 | soil | VIQK01000007.1 | Brazil | 1994 | Monnerat et al. (2019) |
|  | *B. thuringiensis* | SA11 | Biopesticide Delfin | CP083116 | unknown | unknown | Biggel (2021) |
|  | *B. thuringiensis* | SaN0-19 | sediment | JAIVKP010000006.1 | China | 2021 | Yu et al. (2021) |
|  | *B. thuringiensis* | Sbt003 | soil | KN849185.1 | China | 2010 | Liu et al. (2012) |
|  | *B. thuringiensis* | SCG04-02 | soil | CP017577.1 | China | 1992 | Fu and Gao (2016) |
|  | *B. thuringiensis* | 97-27 | unknown | AE017355.1 | unknown | unknown | Brettin et al. (2004) |
|  | *B. thuringiensis* | SID4914 | Fungus Growing Ant Dump | WWJQ01000019.1 | USA | 2015 | Chevrette and Currie (2019) |
|  | *B. thuringiensis* | SRCM116092 | soil | JAOTPD010000001.1 | South Korea | 2020 | Yang et al. (2022) |
|  | *B. thuringiensis* | SRCM116511 | soil | JAOTPC010000001.1 | South Korea | 2020 | Yang et al. (2022) |
|  | *B. thuringiensis* | SRCM125129 | soil | JAQQBL010000001.1 | South Korea | 2022 | Yang et al. (2023) |
|  | ***B. thuringiensis*** | **SRR5713945-bin** | **human gut** | **CAJKZJ010000573.1** | **unknown** | **unknown** | **EMBL-EBI Metagenomics Team (2019)** |
|  | *B. thuringiensis* | SS2 | soil | JAOWLZ010000020.1 | Nigeria | 2017 | Saibu et al. (2022) |
|  | *B. thuringiensis* | ST7 | unknown | CP016194 | unknown | unknown | Zhu (2016) |
|  | *B. thuringiensis* | strain 62 | Coleoptera | PGEF01000099.1 | United Kingdom | 2013 | Pascoe et al. (2017) |
|  | *B. thuringiensis* | SY49-1 | soil | JAHKEZ010000223.1 | Turkey | 2008 | Yilmaz et al. (2021) |
|  | *B. thuringiensis* | T01001 | unknown | CM000748.1 | unknown | unknown | Read et al (2009) |
|  | *B. thuringiensis* | T0131 | soil | CP035735 | Brazil | 2015 | Alves et al. (2019) |
|  | *B. thuringiensis* | T01-328 | soil sample | ARXZ02000005.1 | brazil | 1994 | Varani et al. (2013) |
|  | *B. thuringiensis* | t0137 | soil | CP035736 | Brazil | 2015 | Alves et al. (2019) |
|  | *B. thuringiensis* | T0139 | soil | CP035737 | Brazil | 2015 | Alves et al. (2019) |
|  | *B. thuringiensis* | T03a001 | unknown | CM000751.1 | unknown | unknown | Read et al. (2009)‎ |
|  | *B. thuringiensis* | T04001 | unknown | CM000749.1 | unknown | unknown | Read et al. (2009) |
|  | *B. thuringiensis* | T07005 | unknown | NFEO01000211.1 | unknown | unknown | Zheng et al. (2016) |
|  | *B. thuringiensis* | T07019 | unknown | NFEP01000171.1 | unknown | unknown | Zheng et al. (2016) |
|  | *B. thuringiensis* | T07030 | unknown | NFEQ01000253.1 | unknown | unknown | Zheng et al. (2016) |
|  | *B. thuringiensis* | T07128 | unknown | NFER01000144.1 | unknown | unknown | Zheng et al. (2016) |
|  | *B. thuringiensis* | T07148 | unknown | NFES01000212.1 | unknown | unknown | Zheng et al. (2016) |
|  | *B. thuringiensis* | T07151 | unknown | NFET01000041.1 | unknown | unknown | Zheng et al. (2016) |
|  | *B. thuringiensis* | T07153 | unknown | NFEU01000200.1 | unknown | unknown | Zheng et al. (2016) |
|  | *B. thuringiensis* | T07183 | unknown | NFEV01000200.1 | unknown | unknown | Zheng et al. (2016) |
|  | *B. thuringiensis* | T13001 | unknown | CM000750.1 | unknown | unknown | Read et al. (2009)‎ |
|  | *B. thuringiensis* | T26 | soil | RBKQ01000016.1 | India | 2005 | Reyaz et al. (2018) |
|  | *B. thuringiensis* | T30001 | unknown | MOOV01000141.1 | unknown | unknown | Zheng et al. (2016) |
|  | *B. thuringiensis* | T405 | soil | JAIVKF010000039.1 | India | 2015 | Sathyan et al. (2021) |
|  | *B. thuringiensis* | T414 | soil | RBVK01000040.1 | India | 2005 | Reyaz et al. (‎)2018‎ |
|  | *B. thuringiensis* | T541 | soil | RAQV01000006.1 | India | 2006 | Reyaz et al. (2018) |
|  | *B. thuringiensis* | T63001 | unknown | NFEW01000023.1 | Bolivia | unknown | Zheng et al. (2016) |
|  | *B. thuringiensis* | T69001 | unknown | NFEY01000029.1 | Malaysia | unknown | Zheng et al. (2016) |
|  | *B. thuringiensis* | TAND672 | unknown | CP071743 | unknown | unknown | Geng and Hu (2021) |
|  | *B. thuringiensis* | TB08 | soil | VLPR01000037.1 | Viet Nam | 2015 | Le et al. (2019) |
|  | *B. thuringiensis* | tcg1-2 | soil | DAOJID010000007.1 | Japan | 2011 | Souvorov et al. (2018) |
|  | *B. thuringiensis* | ten BI 256-82 | *Tenebrio molitor* | CP133369.1 | Germany | 1992 | Schaefer et al. (2023) |
|  | *B. thuringiensis* | ten BI 256-82/CM3 | *Tenebrio molitor* | CP133375.1 | Germany | 1990 | Schaefer et al. (2023) |
|  | *B. thuringiensis* | TG-5 | Plant rhizosphere soil | CP110109 | China | 2021 | Du (2022) |
|  | *B. thuringiensis* | TH19 | soil | SZVT01000036.1 | Viet Nam | 2015 | Le et al. (2019) |
|  | *B. thuringiensis* | tky2-1 | soil | DAOJIE010000007.1 | Japan | 2011 | Souvorov et al. (2018) |
|  | *B. thuringiensis* | TOL651 | soil | JANVFA010000020.1 | Brazil | 2017 | Alves (2022) |
|  | *B. thuringiensis* | turex | leaves | JAOAOY010000395.1 | Belgium | 2020 | Zervas (2022) |
|  | *B. thuringiensis* | UFT038 | soil | CP094407 | Brazil | 2018 | Aguiar et al. (2022) |
|  | *B. thuringiensis* | WBt-2 | soil | NHNR01000041.1 | China | 2003 | Shu (2017) |
|  | *B. thuringiensis* | X023 | soil | CP045585 | China | 2018 | Ding et al. (2019) |
|  | *B. thuringiensis* | X023PN | unknown | JAKCLF010000041.1 | China | 2019 | Zhu (2022) |
|  | *B. thuringiensis* | Xentari | Leaves | JAOAOV010000037.1 | Belgium | 2020 | Zervas (2022) |
|  | *B. thuringiensis* | YBT032 | soil | NFEK01000127.1 | China | unknown | Zheng et al. (2016) |
|  | *B. thuringiensis* | YBT-020 | Unknown | CP002508.1 | unknown | unknown | Zhu et al. (2011) |
|  | *B. thuringiensis* | YBT-1518 | unknown | CP005935.1 | unknown | unknown | Wang et al. (2013) |
|  | *B. thuringiensis* | YBT-1520 | unknown | CP004858.1 | China | 1990 | Zhu and Sun (2013) |
|  | *B. thuringiensis* | YC-10 | roots | CP011349 | China | 2010 | Cheng (2015) |
|  | *B. thuringiensis* | YGd22-03 | soil | CP019230.1 | China | 1992 | Wu et al. (2017) |
|  | *B. thuringiensis* | YWC2-8 | soil | CP013055 | China | 2007 | Zhu (2015) |
|  | *B. thuringiensis* | ZZQ-130 | salt lake water | CP089521.1 | China | 2020 | Zheng, 2021 |
|  | *B. thuringiensis* | KB1 | *Arabidopsis thaliana* | LSNJ01000001.1 | South Korea | 2012 | Jeon et al. (2016) |
|  | ***B. thuringiensis*** | **XL6** | **soil** | **CP013000.1** | **China** | **2006** | **Shu et al. (2015)** |

**The strains with low-quality genomes are highlighted in yellow.**
