# Supplementary material for: Taxonomic refinement of Bacillus thuringiensis
Source: Front Microbiol. 2025 Feb 7;16:1518307. doi: 10.3389/fmicb.2025.1518307 (PMC11843730; doi:10.3389/fmicb.2025.1518307)
Supplement: SUPPLEMENTARY TABLE S2 — List of type strains of all validly described Bacillus species used in the study according to the LPSN database https://lpsn.dsmz.de/. [file Table_2.docx]

**Table S2:** **The type strains of all the validly described *Bacillus* species used in The Study**

|  | *B. albus*-N35-10-2 |
| --- | --- |
|  | *B. alkalicellulosilyticus*-FJAT-44921 |
|  | *B. alkalicola*-JCM-17908 |
|  | *B. altitudinis*-41KF2b |
|  | *B. alveayuensis*-24KAM51 |
|  | *B. amyloliquefaciens*-DSM7 |
|  | *B. anthracis*-Ames-Ancestor |
|  | *B. arachidis*-SY8 |
|  | *B. atrophaeus*-BSS |
|  | *B. australimaris*-NH7I_1 |
|  | *B. badius*-NBPM-293 |
|  | *B. benzoevorans*-DSM-5391 |
|  | *B. cabrialesii*-TE3 |
|  | *B. canaveralius*-M4. 6 |
|  | *B. capparidis*-DSM-103394 |
|  | *B. carboniphilus*-SaN35-3 |
|  | *B. cereus*-ATCC-14579 |
|  | *B. changyiensis*-CLL-3-40 |
|  | *B. chungangensis*-DSM-23837 |
|  | *B. coahuilensis*-m4-4 |
|  | *B. cytotoxicus*-NVH-391-98 |
|  | *B. dafuensis*-FJAT-25496-, |
|  | *B. dicomae*-MHSD28 |
|  | *B. ectoiniformans*-DSM-28970 |
|  | *B. fonticola*-CS13 |
|  | *B. fungorum*-17-SMS-01 |
|  | *B. gaemokensis*-KCTC-13318 |
|  | *B. glycinifermentans*-SRCM103574 |
|  | *B. gobiensis*-FJAT-4402 |
|  | *B. hominis*-BML-BC059 |
|  | *B. horti*-DSM-12751 |
|  | *B. inaquosorum*-KCTC-13429 |
|  | *B. infantis*-AcN21-9 |
|  | *B. kexueae*-KCTC-33881 |
|  | *B. licheniformis*-ATCC-14580 |
|  | *B. litorisediminis*-FJAT-47801 |
|  | *B. luti*-TD41 |
|  | *B. manliponensis*-JCM-15802, |
|  | *B. marinisedimentorum*-NC2-31 |
|  | *B. massiliigorillae*-G2 |
|  | *B. mesophilus*-DSM-101000 |
|  | *B. methanolicus*-PB1 |
|  | *B. mexicanus*-FSQ1 |
|  | *B. mobilis*-0711P9-1 |
|  | *B. mojavensis*-ATCC-51516 |
|  | *B. mycoides*-ATCC-6462T |
|  | *B. nakamurai*-NRRL-B-41091 |
|  | *B. ndiopicus*-FF3 |
|  | *B. nitratireducens*-4049 |
|  | *B. aquiflavi*-3H-10 |
|  | *B. haynesii*-NRRL-B-41327 |
|  | *B. oleivorans*-JC228 |
|  | *B. vallismortis*-DSM-11031 |
|  | *B. pacificus*-EB422 |
|  | *B. pakistanensis*-DSM-24834 |
|  | *B. paralicheniformis*-KJ-16 |
|  | *B. paramobilis*-BML-BC017 |
|  | *B. paramycoides*-NH24A2 |
|  | *B. paranthracis*-Mn5 |
|  | *B. pinisoli*-GXH0341 |
|  | *B. piscicola*-FBU1786 |
|  | *B. proteolyticus*-TD42 |
|  | *B. pseudomycoides*-DSM-12442 |
|  | *B. pumilus*-ATCC-7061 |
|  | *B. rhizoplanae*-CIP-111899 |
|  | *B. safensis*.sub.osmophilus-BC09 |
|  | *B. salacetis*-SKP7-4 |
|  | *B. sanguinis*-BML-BC004 |
|  | *B. shivajii*-JCM-32183 |
|  | *B. siamensis*-KCTC-13613 |
|  | *B. sinesaloumensis*-Marseille-P3516 |
|  | *B. smithii*-DSM-4216 |
|  | *B. solimangrovi*-GH2-4 |
|  | *B. sonorensis*-NBRC-101234 |
|  | *B. spizizenii*-NBRC-101239 |
|  | *B. stercoris*-D7XPN1-28 |
|  | *B. suaedae*-YZJH907 |
|  | *B. suaedaesalsae*-RD4P76 |
|  | *B. subtilis*-ATCC-6051 |
|  | *B. swezeyi*-NRRL-B-41294 |
|  | *B. taeanensis*-BH030017 |
|  | *B. tequilensis*-ATCC-BAA-819 |
|  | *B. thermotolerans*-SGZ-8 |
|  | *B. thuringiensis*-ATCC-10792 |
|  | *B. tianshenii*-DSM-25879 |
|  | *B. toyonensis*-BCT-7112 |
|  | *B. tropicus*-N24 |
|  | *B. velezensis*-JS25R |
|  | *B. wiedmannii*-FSL-W8-0169 |
|  | *B. wudalianchiensis*-FJAT-27215 |
|  | *B. xiamenensis*-HYC-10 |
|  | *B. xiapuensis*-FJAT-46582 |
|  | *B. zhangzhouensis*-DW5-4 |
|  | *B. salinus*- HMF5848 |
|  | *B. halotolerans*- ATCC 25096 |
|  | *B. enclensis*-SGD-1123 |
|  | *B. massiliigabonensis*- Marseille-P2639 |
