# Supplementary material for: Taxonomic refinement of Bacillus thuringiensis
Source: Front Microbiol. 2025 Feb 7;16:1518307. doi: 10.3389/fmicb.2025.1518307 (PMC11843730; doi:10.3389/fmicb.2025.1518307)
Supplement: SUPPLEMENTARY TABLE S3 — Digital DNA–DNA hybridization (dDDH; upper diagonal) and average nucleotide identity (ANIm; lower diagonal) values generated from the DNA sequence similarity comparisons among different mislabeled Bacillus thuringiensis strains and the Bacillus anthracis type strain. [file Table_3.docx]

|  | Strain | Taxon | 1 | 2 | 3 | 4 | 5 | 6 | 7 | 8 | 9 | 10 | 11 | 12 | 13 | 14 | 15 | 16 | 17 | 18 | 19 | 20 | 21 | 22 | 23 | 24 | 25 | 26 | 27 | 28 | 29 | 30 | 31 | 32 | 33 | 34 | 35 | 36 | 37 |
| --- | --- | --- | --- | --- | --- | --- | --- | --- | --- | --- | --- | --- | --- | --- | --- | --- | --- | --- | --- | --- | --- | --- | --- | --- | --- | --- | --- | --- | --- | --- | --- | --- | --- | --- | --- | --- | --- | --- | --- |
|  | BtEt101 | *B. thuringiensis* |  | 59.40 | 59.40 | 58.80 | 59.40 | 59.40 | 59.40 | 59.00 | 59.00 | 58.60 | 59.30 | 59.20 | 58.90 | 59.10 | 58.90 | 59.40 | 58.90 | 59.10 | 59.20 | 59.20 | 59.40 | 58.60 | 59.60 | 59.40 | 58.90 | 59.20 | 59.90 | 59.20 | 59.10 | 60.10 | 60.10 | 60.10 | 60.70 | 60.10 | 61.50 | 43.60 | 59.10 |
|  | BtFDAARGOS792 | *B. thuringiensis* | 95.14 |  | 100.00 | 80.50 | 87.60 | 83.60 | 83.60 | 83.50 | 83.50 | 82.30 | 81.10 | 81.80 | 83.20 | 83.70 | 82.70 | 81.50 | 83.60 | 81.90 | 82.20 | 82.10 | 79.80 | 78.30 | 80.20 | 79.70 | 76.30 | 76.70 | 76.50 | 76.30 | 77.20 | 77.00 | 76.90 | 77.00 | 77.50 | 76.10 | 59.60 | 44.40 | 81.10 |
|  | Bt9727 | *B. thuringiensis* | 95.14 | 99.99 |  | 80.50 | 87.60 | 83.60 | 83.60 | 83.50 | 83.50 | 82.30 | 81.10 | 81.80 | 83.20 | 83.70 | 82.70 | 81.50 | 83.60 | 81.90 | 82.20 | 82.10 | 79.80 | 78.30 | 80.20 | 79.70 | 76.30 | 76.80 | 76.50 | 76.30 | 77.20 | 77.00 | 76.90 | 77.00 | 77.50 | 76.10 | 59.60 | 44.40 | 81.10 |
|  | BtBMBT15426 | *B. thuringiensis* | 95.01 | 98.00 | 98.00 |  | 81.80 | 84.10 | 84.10 | 84.20 | 84.20 | 83.20 | 80.90 | 82.50 | 88.60 | 84.20 | 83.60 | 81.40 | 89.00 | 82.50 | 82.60 | 82.60 | 81.60 | 77.70 | 79.80 | 79.50 | 76.50 | 77.30 | 76.70 | 77.20 | 76.00 | 77.00 | 77.00 | 77.00 | 77.30 | 76.50 | 59.40 | 44.90 | 82.40 |
|  | Bt45L | *B. thuringiensis* | 95.13 | 98.73 | 98.37 | 98.08 |  | 83.60 | 83.60 | 84.10 | 84.00 | 82.60 | 80.90 | 82.60 | 83.90 | 83.40 | 82.90 | 81.30 | 84.20 | 82.60 | 82.90 | 82.70 | 80.30 | 78.50 | 80.40 | 80.00 | 76.40 | 77.30 | 76.70 | 77.30 | 77.10 | 77.10 | 77.10 | 77.10 | 77.70 | 76.40 | 58.80 | 44.00 | 81.70 |
|  | BtHD682 | *B. thuringiensis* | 95.08 | 98.32 | 98.31 | 98.34 | 98.33 |  | 100.00 | 92.40 | 92.40 | 91.00 | 85.50 | 85.70 | 87.00 | 92.60 | 91.30 | 85.70 | 87.40 | 85.80 | 86.10 | 85.90 | 80.80 | 80.20 | 83.20 | 82.90 | 78.20 | 78.80 | 78.80 | 78.50 | 78.30 | 79.00 | 79.00 | 79.00 | 79.70 | 78.20 | 60.00 | 44.30 | 87.80 |
|  | BtFDAARGOS794 | *B. thuringiensis* | 95.08 | 98.32 | 98.32 | 98.34 | 98.33 | 99.99 |  | 92.40 | 92.50 | 91.00 | 85.80 | 85.70 | 87.00 | 92.60 | 91.30 | 85.70 | 87.40 | 85.80 | 86.10 | 86.00 | 80.80 | 80.30 | 83.20 | 82.90 | 78.20 | 78.80 | 78.80 | 78.50 | 78.30 | 79.00 | 79.00 | 79.00 | 79.70 | 78.20 | 60.00 | 44.20 | 87.80 |
|  | BtHD1011 | *B. thuringiensis* | 95.04 | 98.33 | 98.33 | 98.38 | 98.36 | 99.17 | 99.17 |  | 100.00 | 90.60 | 85.90 | 85.40 | 86.20 | 99.99 | 90.80 | 86.20 | 86.50 | 85.40 | 85.60 | 85.60 | 81.20 | 80.40 | 83.00 | 82.70 | 77.90 | 78.60 | 78.50 | 78.30 | 78.80 | 78.80 | 78.80 | 78.80 | 79.60 | 78.30 | 59.90 | 44.40 | 88.10 |
|  | BtFDAARGOS795 | *B. thuringiensis* | 95.04 | 98.33 | 98.33 | 98.38 | 98.35 | 99.16 | 99.16 | 99.99 |  | 90.50 | 85.80 | 85.40 | 86.10 | 99.99 | 90.80 | 86.20 | 86.50 | 85.40 | 85.60 | 85.60 | 81.10 | 80.30 | 83.00 | 82.70 | 77.90 | 78.60 | 78.50 | 78.30 | 78.70 | 78.80 | 78.70 | 78.80 | 79.50 | 78.30 | 59.90 | 44.40 | 88.00 |
|  | BtBGSC4AC1 | *B. thuringiensis* | 94.97 | 98.26 | 98.25 | 98.33 | 98.29 | 99.08 | 99.08 | 99.04 | 99.04 |  | 84.40 | 84.20 | 85.30 | 89.30 | 99.90 | 84.90 | 85.40 | 84.30 | 84.70 | 84.60 | 80.00 | 77.60 | 82.50 | 81.80 | 75.60 | 76.30 | 77.10 | 76.20 | 77.30 | 78.10 | 78.10 | 78.10 | 78.70 | 77.50 | 59.40 | 43.80 | 87.60 |
|  | BtBGSC4CE1 | *B. thuringiensis* | 95.10 | 98.11 | 98.12 | 98.08 | 98.09 | 98.49 | 98.49 | 98.53 | 98.52 | 98.50 |  | 82.40 | 82.60 | 85.20 | 84.60 | 99.90 | 82.90 | 82.50 | 82.80 | 82.70 | 80.40 | 78.80 | 81.80 | 81.20 | 77.20 | 77,60 | 76.50 | 77.60 | 77.80 | 78.40 | 78.40 | 78.40 | 79.10 | 77.90 | 60.10 | 43.70 | 87.30 |
|  | BtBGSC4AY1 | *B. thuringiensis* | 95.06 | 98.18 | 98.18 | 98.20 | 98.27 | 98.53 | 98.53 | 98.51 | 98.51 | 98.50 | 98.20 |  | 82.90 | 84.90 | 84.70 | 83.00 | 83.10 | 99.90 | 99.90 | 99.90 | 81.50 | 78.40 | 83.80 | 82.70 | 77.90 | 78.70 | 77.00 | 79.40 | 78.20 | 79.30 | 79.30 | 79.30 | 79.80 | 79.00 | 59.00 | 43.30 | 84.70 |
|  | BtBGSC4AS1 | *B. thuringiensis* | 95.05 | 98.27 | 98.27 | 98.80 | 98.36 | 98.63 | 98.63 | 98.57 | 98.56 | 98.53 | 98.24 | 98.26 |  | 86.20 | 86.00 | 82.90 | 99.90 | 82.90 | 83.00 | 83.00 | 82.10 | 78.20 | 81.30 | 80.90 | 76.60 | 77.50 | 77.90 | 77.80 | 76.90 | 77.70 | 77.70 | 77.70 | 78.20 | 77.30 | 59.50 | 43.90 | 84.50 |
|  | BtBGSC4BA1 | *B. thuringiensis* | 95.04 | 98.33 | 98.33 | 98.38 | 98.28 | 99.53 | 99.18 | 99.99 | 99.99 | 98.99 | 98.49 | 98.49 | 98.85 |  | 89.70 | 85.30 | 86.20 | 85.00 | 85.10 | 85.20 | 80.90 | 79.70 | 83.00 | 81.90 | 77.70 | 78.40 | 78.50 | 78.20 | 78.50 | 78.90 | 78.90 | 78.80 | 79.70 | 78.30 | 59.40 | 44.20 | 88.10 |
|  | BtBGSC4AJ1 | *B. thuringiensis* | 94.99 | 98.28 | 98.28 | 98.36 | 98.31 | 99.10 | 99.10 | 99.07 | 99.07 | 99.99 | 98.50 | 98.50 | 98.57 | 99.01 |  | 85.10 | 85.90 | 84.90 | 85.10 | 85.10 | 80.50 | 78.50 | 82.70 | 82.20 | 76.60 | 77.60 | 77.40 | 77.30 | 77.50 | 78.50 | 78.50 | 78.50 | 79.00 | 77.90 | 59.50 | 43.60 | 88.00 |
|  | BtBGSC4CC1 | *B. thuringiensis* | 95.12 | 98.14 | 98.14 | 98.10 | 98.10 | 98.53 | 98.53 | 98.55 | 98.55 | 98.53 | 99.98 | 98.23 | 98.28 | 98.51 | 98.54 |  | 83.10 | 83.00 | 83.10 | 83.20 | 80.90 | 79.00 | 82.00 | 81.60 | 77.40 | 77.80 | 77.10 | 77.90 | 78.30 | 78.80 | 78.80 | 78.80 | 79.50 | 78.20 | 60.20 | 43.80 | 87.50 |
|  | BtBGSC4AW1 | *B. thuringiensis* | 95.06 | 98.31 | 98.30 | 98,82 | 98.36 | 98.65 | 98.65 | 98.59 | 98.65 | 98.54 | 98.27 | 98.29 | 99.99 | 98.58 | 98.58 | 98.29 |  | 83.00 | 83.10 | 83.20 | 82.30 | 78.80 | 81.50 | 80.70 | 76.80 | 77.80 | 78.10 | 78.10 | 77.00 | 78.20 | 78.20 | 78.20 | 78.80 | 77.60 | 66.00 | 44.20 | 84.70 |
|  | Bt4XX3 | *B. thuringiensis* | 95.04 | 98.18 | 98.18 | 98.20 | 98.28 | 98.53 | 98.53 | 98.52 | 98.52 | 98.47 | 98.21 | 99.98 | 98.27 | 98.50 | 98.50 | 98.24 | 98.29 |  | 99.90 | 99.90 | 81.50 | 78.50 | 83.80 | 82.70 | 77.80 | 78.50 | 77.90 | 79.20 | 78.20 | 79.40 | 79.40 | 79.40 | 79.90 | 79.00 | 59.00 | 43.90 | 84.60 |
|  | Bt4XX2 | *B. thuringiensis* | 95.06 | 98.19 | 98.19 | 98.20 | 98.28 | 98.54 | 98.54 | 98.53 | 98.53 | 98.49 | 98.22 | 99.98 | 98.28 | 98.50 | 98.52 | 98.24 | 98.29 | 99.98 |  | 100.00 | 81.70 | 78.80 | 83.90 | 82.80 | 78.10 | 78.80 | 78.10 | 79.50 | 78.40 | 79.60 | 79.50 | 79.50 | 80.10 | 79.20 | 59.00 | 43.50 | 84.80 |
|  | Bt4XX1 | *B. thuringiensis* | 95.06 | 98.20 | 98.20 | 98.20 | 98.28 | 98.53 | 98.53 | 98.52 | 98.53 | 98.49 | 98.22 | 99.98 | 98.27 | 98.50 | 98.50 | 98.24 | 98.29 | 99.98 | 99.98 |  | 81.60 | 78.80 | 83.90 | 82.90 | 78.00 | 78.70 | 78.00 | 79.50 | 78.40 | 79.50 | 79.50 | 79.50 | 80.00 | 79.10 | 59.10 | 43.40 | 84.80 |
|  | Bt4W2 | *B. thuringiensis* | 95.04 | 97.89 | 97.88 | 98.09 | 97.97 | 98.01 | 98.10 | 98.07 | 98.06 | 97.98 | 97.99 | 98.10 | 98.13 | 98.05 | 98.01 | 98.02 | 98.14 | 98.10 | 98.10 | 98.11 |  | 81.30 | 81.30 | 81.00 | 77.40 | 77.70 | 78.90 | 77.50 | 78.40 | 78.70 | 78.70 | 78.80 | 79.10 | 78.20 | 59.20 | 44.40 | 80.90 |
|  | BtBGSC4BX1 | *B. thuringiensis* | 94.98 | 97.77 | 97.77 | 97.71 | 97.79 | 97.97 | 97.97 | 97.97 | 97.97 | 97.83 | 97.84 | 97.85 | 97.76 | 97.92 | 97.86 | 97.85 | 97.78 | 97.87 | 97.87 | 97.88 | 98.08 |  | 80.60 | 80.10 | 76.70 | 77.70 | 77.00 | 77.80 | 80.80 | 78.80 | 78,80 | 78.80 | 79.30 | 78.30 | 58.40 | 43.40 | 79.60 |
|  | patient-AMN36761919 | *B. thuringiensis* | 95.15 | 97.49 | 97,94 | 97.95 | 97.79 | 98.26 | 98.26 | 98.26 | 98.26 | 98.25 | 98.15 | 98.28 | 98.09 | 98.27 | 98.27 | 98.17 | 98.10 | 98.28 | 98.28 | 98.29 | 98.08 | 98.02 |  | 99.70 | 78.60 | 80.50 | 79.60 | 80.50 | 78.90 | 79.30 | 79.30 | 79.30 | 79.70 | 78.90 | 58.80 | 43.90 | 81.70 |
|  | BtDPC6431 | *B. thuringiensis* | 95.12 | 97.93 | 97.93 | 97.92 | 97.98 | 98.21 | 98.21 | 98.26 | 98.25 | 98.20 | 97.12 | 98.22 | 98.07 | 98.19 | 98.22 | 98.16 | 98.07 | 98.22 | 98.20 | 98.23 | 98.06 | 97.99 | 99,96 |  | 78.10 | 79.60 | 79.20 | 79.50 | 78.50 | 79.10 | 79.00 | 79.00 | 79.40 | 78.70 | 58.70 | 43.60 | 81.50 |
|  | BtG2553 | *B. thuringiensis* | 95.34 | 97.65 | 97.65 | 97.63 | 97.67 | 97.86 | 97.85 | 97.83 | 97.82 | 97.72 | 97.74 | 97.83 | 97.69 | 97.81 | 97.78 | 97.75 | 97.71 | 97.82 | 97.84 | 97.84 | 97.74 | 97.71 | 97.84 | 97.84 |  | 90.10 | 84.80 | 90.00 | 76.20 | 86.20 | 86.20 | 86.20 | 86.40 | 85.90 | 57.90 | 45.40 | 77.30 |
|  | BtG2552 | *B. thuringiensis* | 95.11 | 97.67 | 97.67 | 97,65 | 97.68 | 97.89 | 97.88 | 97.87 | 97.86 | 97.75 | 97.77 | 97.87 | 97.72 | 97.85 | 97.79 | 97.77 | 97.75 | 97.86 | 97.87 | 97.88 | 97.77 | 97.77 | 97.94 | 97.93 | 99.10 |  | 86.00 | 98.20 | 76.90 | 86.70 | 86.70 | 86.70 | 87.10 | 86.50 | 57.60 | 43.60 | 78.30 |
|  | BtDE0163 | *B. thuringiensis* | 95.12 | 97.56 | 97.56 | 97.56 | 97.64 | 97.79 | 97.79 | 97.79 | 97.79 | 97.70 | 97.57 | 97.72 | 97.67 | 97.90 | 97.72 | 97.61 | 97.70 | 97.74 | 97.74 | 97.74 | 97.79 | 97.64 | 97.85 | 97.83 | 98.55 | 98.59 |  | 85.50 | 76.50 | 91.10 | 91.10 | 91.10 | 91.50 | 90.80 | 58.50 | 43.90 | 78.00 |
|  | BtG2542 | *B. thuringiensis* | 95.11 | 95.68 | 97.68 | 97.65 | 97.71 | 97.90 | 97.89 | 97.88 | 97.87 | 97.75 | 97.77 | 97.90 | 97.73 | 97.86 | 97.80 | 97.79 | 97.76 | 97.89 | 97.91 | 97.91 | 97.75 | 97.79 | 97.95 | 97.96 | 99.08 | 99.85 | 98.59 |  | 76.90 | 86.70 | 86.60 | 86.70 | 87.10 | 86.50 | 57.80 | 43.70 | 78.50 |
|  | BtDE0537 | *B. thuringiensis* | 95.07 | 97.63 | 97.63 | 97.52 | 97.66 | 97.75 | 97.75 | 97.77 | 97.77 | 97.73 | 97.72 | 97.77 | 97.63 | 97.76 | 97.74 | 97.75 | 97.63 | 97.78 | 97.78 | 97.78 | 97.80 | 98.05 | 97.80 | 97.79 | 97.60 | 97.63 | 97.52 | 97.64 |  | 77.30 | 77.30 | 77.30 | 77.70 | 76.80 | 58.50 | 43.90 | 77.60 |
|  | BtFDAARGOS793 | *B. thuringiensis* | 95.15 | 97.60 | 97.60 | 97.60 | 97.65 | 97.81 | 97.81 | 97.80 | 97.79 | 97.77 | 97.74 | 97.82 | 97.67 | 97.78 | 97.79 | 97.77 | 97.69 | 97.83 | 97.83 | 97.83 | 97.75 | 97.78 | 97.81 | 97.80 | 98.62 | 98.60 | 99.03 | 98.59 | 97.56 |  | 100.00 | 100.00 | 100.00 | 94.10 | 58.80 | 44.30 | 78.30 |
|  | BtAlHakam | *B. thuringiensis* | 95.14 | 97.60 | 97.60 | 97.60 | 97.65 | 97.80 | 97.80 | 97.80 | 97.79 | 97.76 | 97.74 | 97.82 | 97.67 | 97.78 | 97.79 | 97.77 | 97.69 | 97.83 | 97.83 | 97.83 | 97.75 | 97.78 | 97.81 | 97.81 | 98.62 | 98.60 | 99.03 | 98.59 | 97.57 | 99.99 |  | 100.00 | 99.90 | 94.10 | 58.80 | 44.30 | 78.30 |
|  | BtHD571 | *B. thuringiensis* | 95.15 | 97.60 | 97.60 | 97.60 | 97.35 | 97.81 | 97.81 | 97.80 | 97.79 | 97.77 | 97.74 | 97.82 | 97.67 | 97.78 | 97.78 | 97.77 | 97.69 | 97.83 | 97.83 | 97.83 | 97.75 | 97.78 | 97.81 | 97.80 | 98.62 | 98.60 | 99.03 | 98.60 | 97.56 | 99.99 | 99.99 |  | 100.00 | 94.10 | 58.80 | 44.30 | 78.30 |
|  | BtFDAARGOS791 | *B. thuringiensis* | 95.25 | 97.66 | 97.66 | 97.65 | 97.73 | 97.91 | 97.91 | 97.89 | 97.88 | 97.85 | 97.82 | 97.88 | 97.74 | 97.87 | 97.86 | 97.85 | 97.75 | 97.89 | 97.90 | 97.89 | 97.80 | 97.84 | 97.86 | 97.84 | 98.66 | 98.64 | 99.09 | 98.65 | 97.61 | 99.99 | 99.99 | 99.99 |  | 94.10 | 59.80 | 45.30 | 78.90 |
|  | Bt4B3 | *B. thuringiensis* | 95.13 | 97.52 | 97.52 | 97.54 | 97.60 | 97.73 | 97.74 | 97.74 | 97.74 | 97.70 | 97.69 | 97.77 | 97.62 | 97.75 | 97.72 | 97.70 | 97.65 | 97.77 | 97.78 | 97.77 | 97.70 | 97.72 | 97.79 | 97.79 | 98.60 | 98.58 | 98.98 | 98.57 | 97.55 | 99.32 | 99.32 | 99.32 | 99.32 |  | 58.60 | 44.10 | 78.00 |
|  | BtXL6 | *B. thuringiensis* | 95.45 | 95.05 | 95.05 | 94.98 | 94.95 | 95.07 | 95.07 | 95.03 | 95.06 | 95.04 | 91.13 | 94.94 | 94.98 | 95.03 | 95.04 | 95.14 | 95.00 | 94.96 | 94.96 | 94.96 | 94.92 | 94.89 | 94.91 | 94.90 | 94.83 | 94.79 | 94.83 | 94.82 | 94.94 | 94.89 | 94.89 | 94.89 | 95.06 | 94.85 |  | 44.90 | 59.60 |
|  | ATCC-10792^T^ | *B. thuringiensis* | 91.60 | 91.73 | 91.73 | 91.87 | 91.60 | 91.69 | 91.68 | 91.72 | 91.71 | 91.60 | 91.64 | 91.53 | 91.63 | 91.66 | 91.57 | 91.63 | 91.65 | 91.57 | 91.53 | 91.53 | 91.66 | 91.60 | 91.60 | 91.59 | 91.72 | 91.59 | 91.53 | 91.65 | 91.61 | 91.69 | 91.69 | 91.69 | 91.92 | 91.61 | 91.90 |  | 44.30 |
|  | Ames-Ancestor^T^ | *B. Anthracis* | 95.02 | 98.12 | 98.12 | 98.24 | 98.17 | 98.77 | 98.77 | 98.80 | 98.80 | 98.80 | 98.68 | 98.41 | 98.13 | 98.80 | 98.82 | 98.67 | 98.47 | 98.41 | 98.41 | 98.41 | 98.00 | 97.88 | 98.13 | 98,14 | 97.73 | 97.79 | 97.67 | 97.81 | 97.69 | 97.73 | 97.73 | 97.73 | 97.80 | 97.67 | 95.02 | 91.69 |  |
