# Supplementary material for: Taxonomic refinement of Bacillus thuringiensis
Source: Front Microbiol. 2025 Feb 7;16:1518307. doi: 10.3389/fmicb.2025.1518307 (PMC11843730; doi:10.3389/fmicb.2025.1518307)
Supplement: SUPPLEMENTARY FIGURE S1 — MLSA-based phylogenetic tree using the sequences of five housekeeping genes gyrB, atpD, dnaK, rpoB, and rpoD for all Bacillus thuringiensis strains retrieved from the NCBI database in this study. [file Data_Sheet_1.pdf]

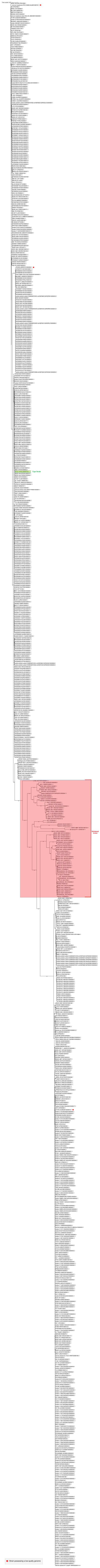

Tree scale: 0.01
Type Strain
Mislabeled strains
Strain possessing a low-quality genome
